# DNA‐Based Hydrogels for Musculoskeletal Reconstruction: Harnessing Dynamic Programmability and Multimodal Therapeutic Integration

**DOI:** 10.1002/advs.202511099

**Published:** 2025-09-08

**Authors:** Ruijianghan Shi, Huilu Zhan, Shan Jiang, Kaili Lin, Changyong Yuan

**Affiliations:** ^1^ Department of Oral and Craniomaxillofacial Surgery Shanghai Ninth People's Hospital Shanghai Jiao Tong University School of Medicine College of Stomatology Shanghai Jiao Tong University National Center for Stomatology National Clinical Research Center for Oral Diseases Shanghai Key Laboratory of Stomatology Research Unit of Oral and Maxillofacial Regenerative Medicine Chinese Academy of Medical Sciences Shanghai 200125 China; ^2^ Department of Stomatology Shanghai General Hospital Shanghai Jiao Tong University School of Medicine Shanghai 201620 China; ^3^ Shenzhen Stomatology Hospital (Pingshan) of Southern Medical University Shenzhen Clinical College of Stomatology School of Stomatology Southern Medical University Shenzhen Guangdong 510515 China; ^4^ School of Stomatology Xuzhou Medical University Affiliated Stomatological Hospital of Xuzhou Medical University Xuzhou 221004 China

**Keywords:** DNA‐based hydrogel, dynamic crosslinking, musculoskeletal regeneration, therapeutic delivery, tissue engineering

## Abstract

Musculoskeletal disorders, including bone fractures, osteoarthritis, and muscle injuries, represent a leading cause of global disability, revealing the urgency for advanced therapeutic solutions. However, current therapies face limitations including donor‐site morbidity, immune rejection, and inadequate mimicry of dynamic tissue repair processes. DNA‐based hydrogels emerge as transformative platforms for musculoskeletal reconstruction, with their sequence programmability, dynamic adaptability, and biocompatibility to balance structural support and biological functions. These hydrogels are classified into two categories: 1) DNA hydrogels, where DNA serves as the structural backbone; 2) DNA component‐loaded hydrogels, integrating functional DNA elements like aptamers and therapeutic genes into non‐DNA matrices. Through dynamic crosslinking strategies, primarily Watson‐Crick base pairing, DNA networks achieve shear‐thinning injectability and self‐healing behaviors while providing binding sites for bioactive DNA components. Hybrid systems further enhance functionality by incorporating diverse materials to improve mechanical strength, drug delivery, and cellular guidance. This review systematically examines molecular design principles, classification frameworks, and preclinical applications of DNA‐based hydrogels, aiming to bridge gaps between material innovation and clinical translation. Finally, current challenges are highlighted, and future directions to advance these intelligent biomaterials toward next‐generation musculoskeletal therapies are proposed.

## Graphical abstract

DNA‐based hydrogels serve dual roles as structural scaffolds and therapeutic platforms, offering innovative solutions for musculoskeletal regeneration with multifunctional abilities.

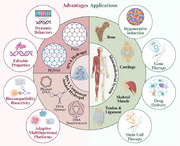

## 1 Introduction

Musculoskeletal disorders such as bone fractures, osteoarthritis, cartilage degeneration, and muscle injuries, represent a leading cause of global disability.^[^ ^1^ ^]^ According to the data from the Global Burden of Disease (GBD) Study 2017, there were 1.3 billion prevalent cases, 121.3 thousand deaths, and 138.7 million disability‐adjusted life years (DALYs) due to musculoskeletal disorders globally.^[^ ^2^ ^]^ These issues impose immense socioeconomic burdens and diminish the quality of life for billions. Current clinical treatment methods have limitations that damage their long‐term efficacy, including autografts, allografts, and synthetic implants.^[^ ^3^, ^4^ ^]^ Autografts, while considered the gold standard for bone repair, suffer from donor‐site morbidity, limited availability, and surgical complications.^[^ ^5^ ^]^ Allografts carry risks of immune rejection, pathogen transmission, and incomplete integration with host tissues.^[^ ^6^ ^]^ Synthetic materials often fail to replicate the dynamic biological processes necessary for tissue regeneration, resulting in adverse treatment outcomes.^[^ ^7^ ^]^ These challenges highlight the urgent need for biomaterial platforms that not only replace lost tissue but also actively balance the complex interplay of musculoskeletal healing.

Hydrogels are three‐dimensional (3D) hydrophilic networks capable of retaining large amounts of water. They have emerged as a heating topic in tissue engineering due to their structural similarity to native extracellular matrix (ECM).^[^ ^8^ ^]^ However, traditional hydrogels fall short of dynamically adapting to evolving biological demands while providing structural support, biochemical signaling, and spatiotemporal control over therapeutic delivery. The advancement of deoxyribonucleic acid (DNA) nanotechnology provides new ideas for the design of biomaterials.^[^ ^9^, ^10^ ^]^ DNA, beyond its biological role as a genetic material, exhibits unique physicochemical properties that make it an ideal building block for next‐generation hydrogels, including sequence programmability, molecular recognition, and dynamic reconfigurability^[^ ^11^, ^12^ ^]^ DNA‐based hydrogels transcend the limitations of conventional systems by integrating structural versatility with inherent bio‐functionality.

To provide a more comprehensive overview of the application prospects of these hydrogels, this review employs the umbrella term “DNA‐based hydrogel” to encompass all hydrogel matrices containing DNA materials. Within this broad category, we present a detailed classification. DNA‐based hydrogels can be divided into two fundamental categories based on how the DNA material participates in the hydrogel composition: 1) “DNA hydrogels,” where DNA serves as the structural backbone; 2) “DNA component‐loaded hydrogels” within non‐DNA matrices. The former utilizes DNA's programmable assembly to create dynamically responsive networks. The dynamic structure enables mechanical adaptation, such as shear‐thinning for injectable delivery^[^ ^13^ ^]^ followed by rapid self‐healing^[^ ^14^ ^]^ at the target site, particularly advantageous for filling irregular defects. The latter harnesses DNA's inherent bioactivities and molecular recognition capabilities, holding promises for targeted therapy.^[^ ^15^ ^]^ These approaches address critical challenges in musculoskeletal repair, such as reconciling mechanical strength with bioactivity, enabling controlled release of therapeutic agents, and providing microenvironmental cues to guide stem cell differentiation and other kinds of biological effects.

**Figure 1:**
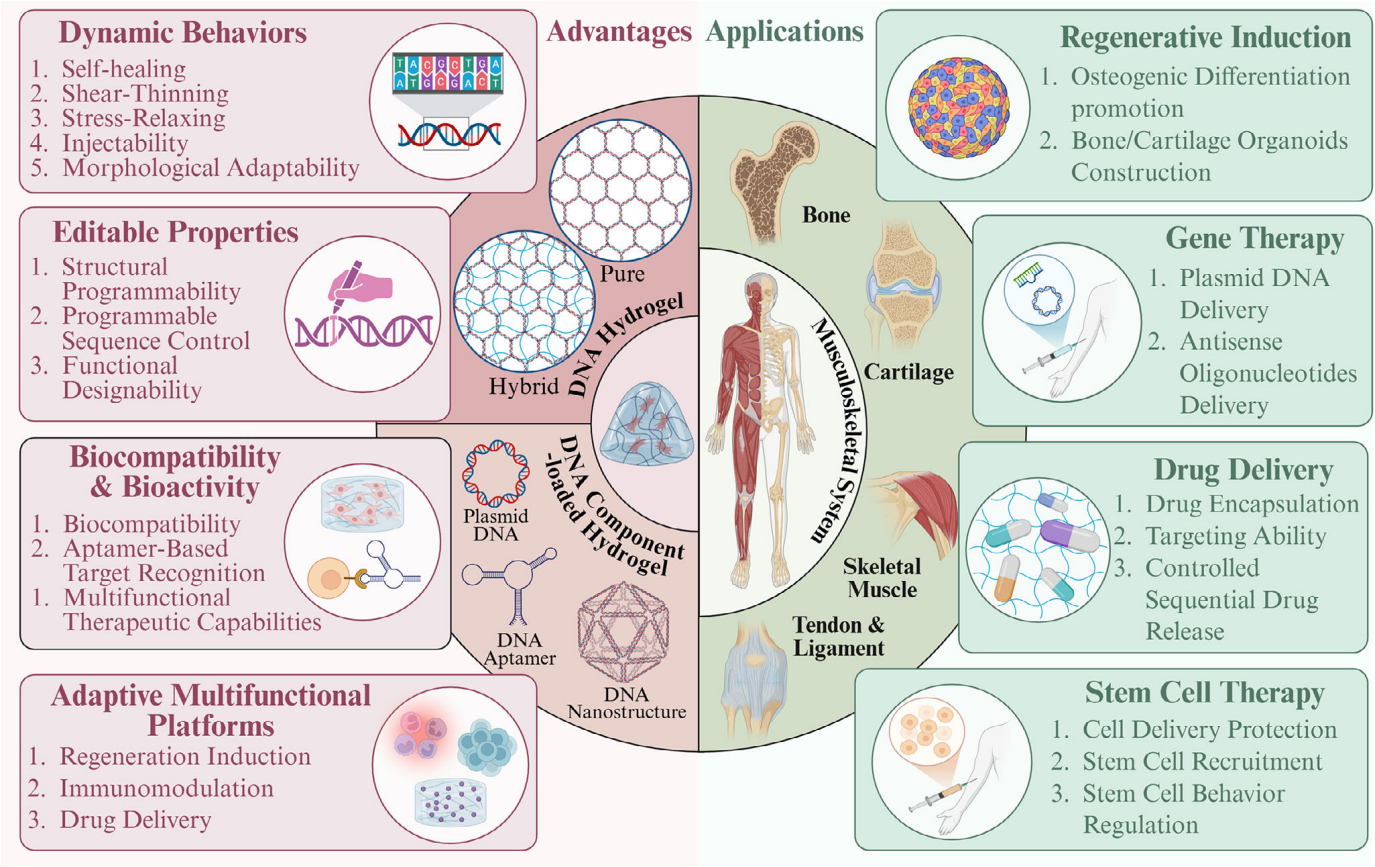
Advantages and application strategies of DNA‐based hydrogels for musculoskeletal reconstruction. Created with https://BioRender.com/.

Existing reviews comprehensively address DNA‐based hydrogel synthesis and physicochemical properties, providing foundational insights into physicochemical properties such as shear‐thinning behavior and dynamic crosslinking mechanisms.^[^ ^16^, ^17^, ^18^, ^19^ ^]^ However, they inadequately explore design strategies for complex tissue regeneration, especially for musculoskeletal regeneration, which is experiencing accelerated translational demand. To systematically address this research deficiency, this review explores the evolution of DNA hydrogels from conceptual frameworks to preclinical applications in musculoskeletal regeneration (**Figure** **1**). We analyze their molecular design principles and evaluate how these architectures align with the requirements of musculoskeletal healing. To our knowledge, this is the first comprehensive analysis positioning DNA‐based hydrogels as potential regulators for musculoskeletal regeneration, including microenvironmental responsiveness under pathological conditions, precise modulation of regenerative processes, and three‐dimensional organoid fabrication. By reviewing recent advances, we aim to overcome current limitations and accelerate clinical translation.

## 2 DNA‐Based Hydrogels: Synthesis and Classification

DNA‐based hydrogels represent a class of intelligent biomaterials that utilize DNA as the core functional component. Based on the functional role of DNA within the hydrogel system, they can be categorized into DNA hydrogels and DNA component‐loaded hydrogels.

**Figure 2:**
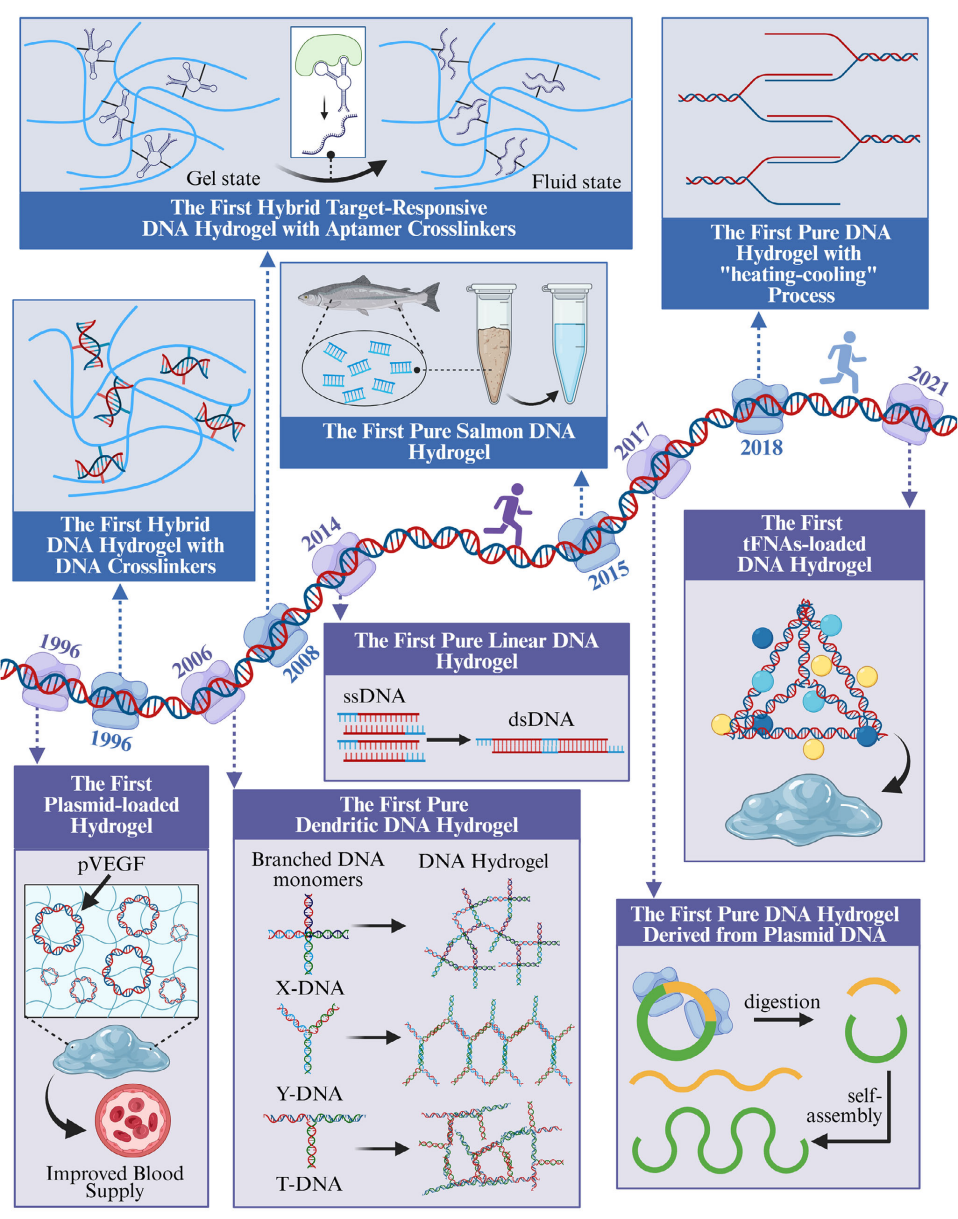
Historical timeline of major developments in DNA‐based hydrogel for regenerative medicine. Created with https://BioRender.com/.

As an emerging technology, DNA‐based hydrogels have a development history spanning less than three decades (**Figure** **2**). In 1996, Nagahara and Matsuda^[^ ^20^ ^]^ pioneered the use of DNA as a crosslinker for polyacrylamide chains, fabricating the first DNA hydrogel in history. That same year, Inser et al.^[^ ^21^ ^]^ successfully improved blood supply to ischemic limbs by loading plasmid DNA onto the hydrogel polymer coating of an angioplasty balloon, marking the first attempt in the field of DNA‐component‐loaded hydrogels. Entering the 21st century, DNA‐based hydrogel technology has experienced vigorous development. For instance, in 2006, Luo et al.^[^ ^22^ ^]^ first developed a pure DNA hydrogel constructed from DNA‐branched monomers. Although their hydrogel required enzymatic ligation, this architectural paradigm provided an important reference model for self‐assembled dendritic DNA hydrogels. Since then, DNA hydrogel synthesis methodologies have undergone significant innovation. Researchers have successively developed rolling circle amplification (RCA)‐based DNA hydrogels,^[^ ^23^ ^]^ sticky‐end‐mediated based hydrogels,^[^ ^24^ ^]^ and salmon DNA‐based self‐assembled hydrogels.^[^ ^25^ ^]^ With the discovery and application of novel DNA components, hydrogels incorporating functional elements such as aptamers^[^ ^26^ ^]^ and tetrahedral framework nucleic acids (tFNAs)^[^ ^27^ ^]^ have also been implemented in biological applications.

This section systematically elaborates on the designing strategies and classification frameworks of these two classes of DNA‐based hydrogels, with a focus on the central role of DNA components. The former emphasizes the construction logic of DNA as a structural network backbone, while the latter highlights the biomedical application potential of DNA as functional modules.

### 2.1 DNA Hydrogel

DNA hydrogels harness the unique molecular recognition properties and programmability of DNA chains to construct dynamic, 3D networks. The following sections delve into the classification of DNA hydrogels based on crosslinking strategies and material components, providing a foundation for understanding their diverse applications and future potential.

#### 2.1.1 Classification Based on Crosslinking Mechanisms

**Figure 3:**
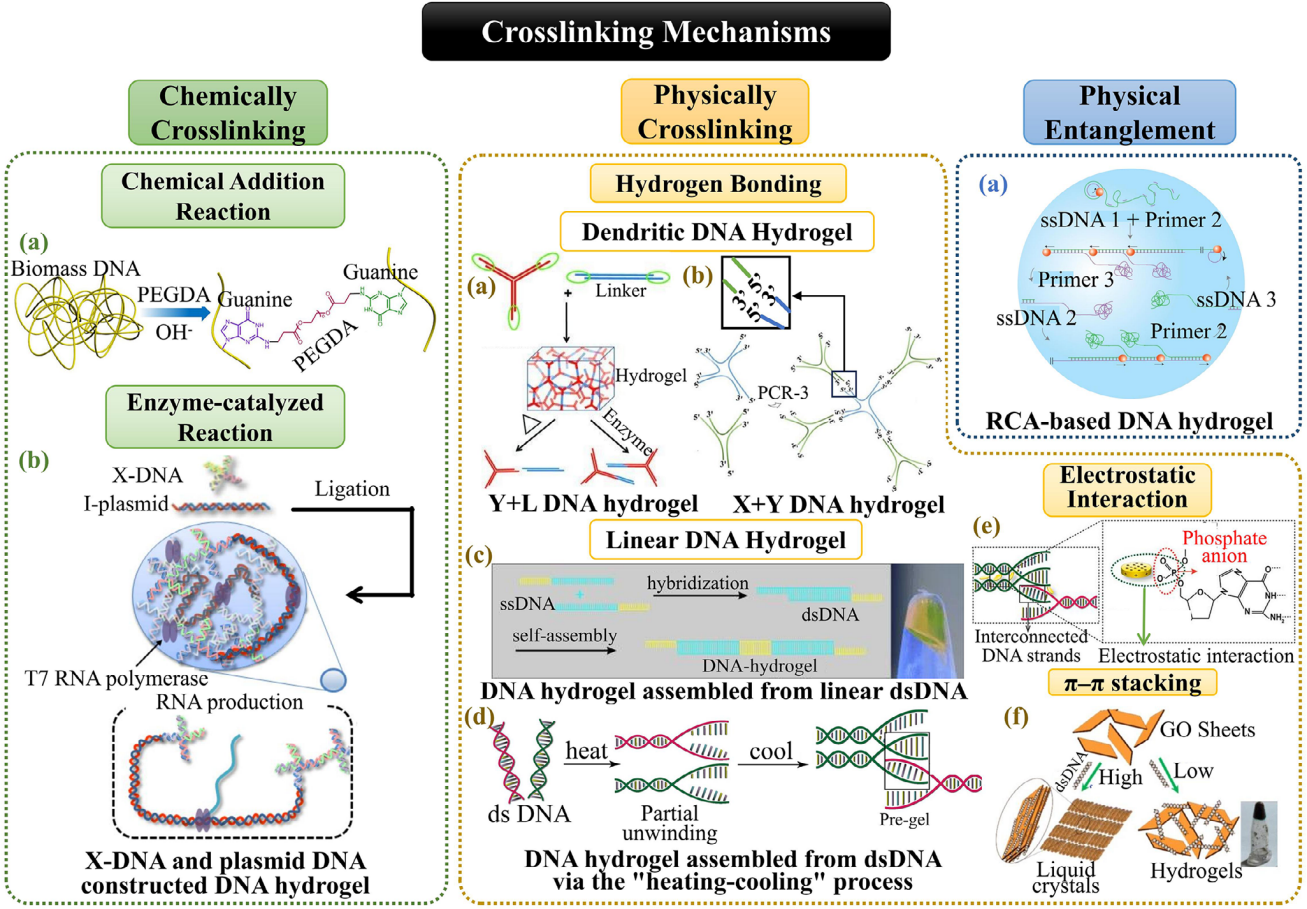
The classification of DNA hydrogel based on crosslinking mechanisms. Chemically Crosslinking: a) DNA crosslinked‐PEGDA hydrogel. Reproduced with permission.^[^ ^29^ ^]^ Copyright 2020, ACS. b) DNA hydrogel constructed with X‐DNA and plasmids using T4 DNA ligase for RNA interference. Adapted under the terms of the CC BY‐NC‐ND license 4.0 from Ref. [31] Copyright 2018, Springer Nature. Physically Crosslinking: a) Y+L DNA hydrogel. Reproduced with permission.^[^ ^33^ ^]^ Copyright 2011, Wiley. B) X+Y DNA hydrogel. Reproduced with permission.^[^ ^40^ ^]^ Copyright 2021, Wiley. c) DNA hydrogel assembled from linear DNA. Reproduced with permission.^[^ ^24^ ^]^ Copyright 2014, Wiley. d) DNA hydrogel assembled via the “heating‐cooling” process. **e** Hybrid DNA hydrogel with electrostatic interaction. Reproduced with permission.^[^ ^34^ ^]^ Copyright 2018, ACS. **f** Hybrid DNA hydrogel with π–π stacking effects. Reproduced with permission.^[^ ^36^ ^]^ Copyright 2016, Springer Nature. Physical Entanglement: a) RCA‐based DNA hydrogel. Reproduced with permission.^[^ ^23^ ^]^ Copyright 2012, Springer Nature.

The functional and mechanical properties of DNA hydrogels are intrinsically linked to their crosslinking strategies, which govern network formation and stability. DNA hydrogels can be broadly categorized into chemically crosslinked, physically crosslinked, and physical entanglement systems, each defined by the nature of interactions between DNA strands (**Figure** **3**).

##### Chemically Crosslinked DNA Hydrogels

Chemically crosslinked DNA hydrogels typically utilize DNA chains, such as salmon sperm DNA, as the principal structural component, establishing stable 3D networks via covalent bonding.^[^ ^28^ ^]^ The mechanical properties of these hydrogels can be precisely controlled through the modulation of crosslinking density.

One approach to forming chemical crosslinking is to employ conventional chemical addition methods by introducing chemical crosslinking agents to form polymers. For instance, Yang et al.^[^ ^29^ ^]^ demonstrated the use of polyethylene glycol diacrylate (PEGDA) to crosslink amino groups on DNA via a Michael addition reaction, yielding hydrogels with tensile moduli in the Mega Pascal (MPa) range, making them promising candidates for applications such as bone defect repair, where mechanical support is crucial.

Another approach of chemical crosslinking involves enzymatic crosslinking, where enzymes such as T4 DNA ligase catalyze the ligation of sticky ends on branched DNA structures.^[^ ^22^ ^]^ This enzymatic approach not only facilitates the formation of large‐scale 3D DNA hydrogels from branched DNA monomers but also enables the incorporation of functionalized nucleic acids, such as plasmid DNA,^[^ ^30^ ^]^ messenger RNA (mRNA),^[^ ^31^ ^]^ small interfering RNA (siRNA),^[^ ^31^ ^]^ and specific nucleotide motifs.^[^ ^32^ ^]^

However, chemically crosslinked DNA hydrogels exhibit limitations. The irreversible covalent bonds between DNA chains result in a permanent crosslinked structure, which lacks shear‐thinning behavior and thus precludes their use in minimally invasive injection applications. Additionally, the absence of self‐healing properties in these hydrogels limits their ability to adapt to irregular defects and makes them more vulnerable to mechanical stress.

##### Physically Crosslinked DNA Hydrogels

In contrast, physically crosslinked DNA hydrogels are formed through non‐covalent interactions between DNA strands, assembled via self‐assembly processes. The high degree of tunability of DNA hydrogels enables a diverse range of self‐assembly strategies.

The primary formation mechanism of pure DNA hydrogels involves crosslinking complementary base sequences to adjacent DNA strands via hydrogen bonding. Two main types of these physically crosslinked pure DNA hydrogels are currently employed in regenerative medicine: dendritic DNA hydrogels and linear DNA hydrogels.^[^ ^18^ ^]^

Dendritic DNA hydrogels are typically constructed from branched DNA monomers, which consist of single‐stranded (ssDNA) chains with sticky ends, crosslinked into networks through the base‐pairing principle. A well‐established design is the Y+L DNA hydrogel, which utilizes Y‐shaped scaffolds and L‐shaped linkers as the basic building units, with self‐gelation occurring upon mixing at specific ratios.^[^ ^33^ ^]^ The mechanical strength of these hydrogels can be modulated by adjusting the monomer ratio and concentration, and is further influenced by the length of terminal groups and the mismatch ratio.

Linear DNA hydrogels are characterized by long dsDNA as the core structural element, and can be formed through several distinct strategies. One approach involves using short linear dsDNA with sticky ends as building blocks, which self‐assemble into long‐chain structures, thereby forming a linear DNA hydrogel.^[^ ^24^ ^]^ Another method is called a “heating‐cooling” process. This method involves denaturing dsDNA by heating to 95 °C, which can be extracted from salmon sperm, to induce strand separation, followed by annealing to facilitate re‐hybridization of the DNA backbone, forming a crosslinked network through hydrogen bonds.^[^ ^34^ ^]^

Building on this foundation, hybrid hydrogels with tunable properties can be developed by introducing reversible cross‐linking strategies, such as electrostatic interactions^[^ ^35^ ^]^ and π–π stacking effects,^[^ ^36^ ^]^ to incorporate novel materials and structures into DNA networks.^[^ ^16^ ^]^

Despite the diversity of design strategies, physically crosslinked DNA hydrogels share common features arising from the reversible interactions. These hydrogels exhibit dynamic responsiveness and self‐healing capabilities, attributed to non‐covalent interactions, such as hydrogen bonds. Consequently, these abilities make them suitable for minimally invasive injection and adapting to a wide range of defect areas in regenerative applications. Furthermore, they can maintain mechanical functionality over extended periods.

##### DNA Hydrogels Based on Physical Entanglement

Additionally, a unique approach to constructing DNA hydrogels relies on physical entanglement, which arises from the inherent flexibility of long DNA chains, enabling them to intertwine and form specific 3D architectures. RCA provides a distinctive mechanism for generating such pure hydrogels.^[^ ^37^ ^]^ RCA extends primer DNA by repetitively replicating a circular DNA template while continuously displacing the newly synthesized strand, thereby producing ultra‐long DNA chains through iterative amplification. These DNA strands become entangled and woven into a 3D hydrogel network. Studies by Lee et al.^[^ ^23^ ^]^ demonstrated that such DNA hydrogels can undergo rapid, reversible transitions between liquid and solid states in response to external environmental stimuli, such as H_2_O molecules, making them ideal for controlled drug release. Furthermore, by integrating additional cross‐linking mechanisms, these hydrogels can incorporate functional molecules like hemoglobin^[^ ^38^ ^]^ or silver ions,^[^ ^39^ ^]^ significantly broadening their biomedical applications and enhancing biological efficacy.

In summary, chemically crosslinked hydrogels rely on covalent bonds for structural integrity, enabling precise control over mechanical strength but often sacrificing dynamic adaptability. In contrast, physically crosslinked hydrogels exploit non‐covalent interactions to achieve self‐healing and shear‐thinning behavior.

#### 2.1.2 Classification Based on Material Composition

**Figure 4:**
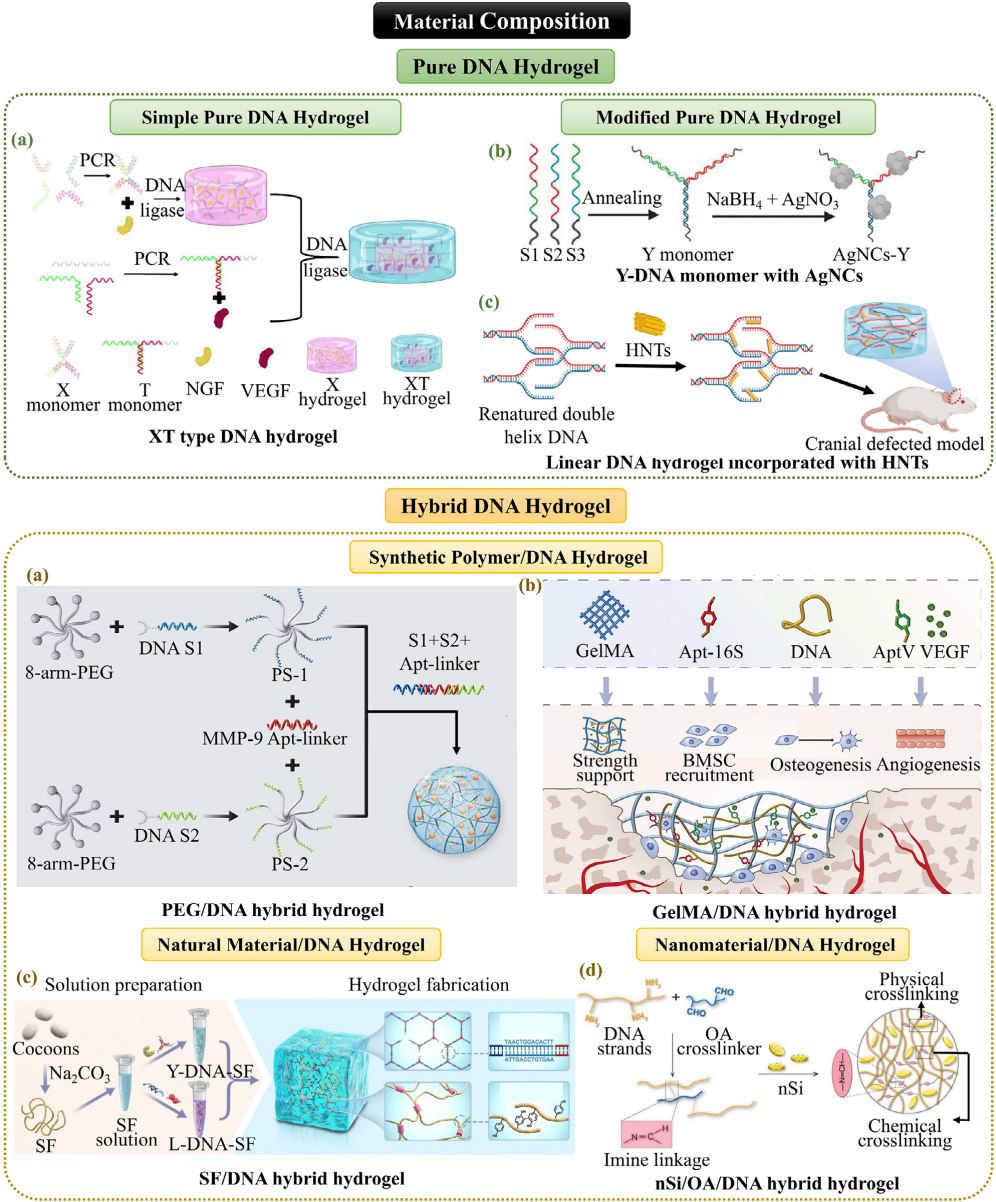
The classification of DNA hydrogel based on material composition. Pure DNA Hydrogel: a) XT‐type DNA hydrogel. Reproduced with permission.^[^ ^41^ ^]^ Copyright 2021, RSC. b) Y‐DNA monomer with AgNCs. Reproduced with permission.^[^ ^45^ ^]^ Copyright 2024, Wiley. c) Linear DNA hydrogel incorporated with HNTs. Reproduced with permission.^[^ ^42^ ^]^ Copyright 2024, Wiley. Hybrid DNA Hydrogel: a) PEG/DNA hybrid hydrogel. Reproduced with permission.^[^ ^47^ ^]^ Copyright 2022, RSC. b) GelMA/DNA hybrid hydrogel. Adapted under the terms of the CC BY‐NC‐ND license 4.0 from Ref. [49] Copyright 2024, KeAi Publishing. c) SF/DNA hybrid hydrogel. Reproduced with permission.^[^ ^51^ ^]^ Copyright 2024, RSC. d) nSi/OA/DNA hybrid hydrogel. Reproduced with permission.^[^ ^52^ ^]^ Copyright 2024, Elsevier.

DNA hydrogels can also be classified by their material composition, which directly influences their biological functionality and mechanical performance. This section divides DNA hydrogel into two categories, pure and hybrid DNA hydrogels, to discuss their molecular design principles according to the different musculoskeletal application scenarios (**Figure** **4**).

##### Pure DNA Hydrogels: Construction and Functionalization Strategies

Pure DNA hydrogels are composed exclusively of DNA strands without the incorporation of other substances. Their fabrication relies on the self‐assembly properties of DNA molecules, facilitated by physical crosslinking and the precise design of nucleotide sequences.

The construction design imparts distinct advantages to pure DNA hydrogels. As previously noted, Y‐type scaffolds (Y‐scaffolds) combined with linear linker chains (L‐linkers) rapidly self‐assemble into a 3D network under physiological conditions. This Y+L‐type DNA hydrogel not only exhibits shear‐thinning properties but also allows precise modulation of mechanical strength. Similarly, the XT‐type DNA hydrogel utilizes phosphate‐linked X‐type monomers to form a rigid network, while T‐type monomers provide flexible crosslinking sites.^[^ ^41^ ^]^ The differential degradation rates of the two DNA monomers facilitate the time‐controlled release of bioactive factors and effectively mimic tissue repair processes. There are also various construction methods of linear DNA hydrogels. Some researchers have utilized salmon sperm DNA to fabricate internally cross‐linked linear DNA hydrogels through a thermal denaturation‐annealing process.^[^ ^42^ ^]^ Alternatively, other investigators have employed fermentation processes to mass‐produce plasmid DNA, then obtained linear DNA building blocks via enzymatic digestion of the plasmid DNA.^[^ ^43^ ^]^ These methods use large‐scale production to replace the costly preparation of customized DNA sequences, thus achieving significant cost reduction in manufacturing.

To address the mechanical and functional limitations of pure DNA hydrogels, various modification strategies have been explored. Incorporation of nanomaterials such as black phosphorus nanosheets (BPNSs)^[^ ^44^ ^]^ and halloysite nanotubes (HNTs)^[^ ^42^ ^]^ enhances the crosslinking with the DNA network via π–π stacking and hydrogen bonding interactions, effectively improving the mechanical strength. Furthermore, through molecular loading and surface modifications, pure DNA hydrogels can be endowed with multifunctional therapeutic properties. For example, in situ synthesis of silver nanoclusters (AgNCs) within the cytosine rings of Y‐type DNA imparts antibacterial properties to the hydrogel,^[^ ^45^ ^]^ while the chemical covalent attachment of neuroadhesive peptides (IKVAV) to the DNA chain enhances neuronal cell attachment and differentiation on the hydrogel.^[^ ^46^ ^]^ These strategies highlight the potential for fine‐tuning the mechanical properties of pure DNA hydrogels, as well as expanding their therapeutic versatility.

##### Hybrid DNA Hydrogels: Dual‐Network Systems

Hybrid DNA hydrogels integrate the programmability and biocompatibility of DNA with other inorganic or organic materials, offering enhanced mechanical adaptability and multiple functions. The construction of hybrid DNA hydrogels typically involves the creation of a “material‐DNA” dual‐network system, which can be categorized based on the type of materials incorporated, including polymer composites, natural material composites, and nanocomposites (**Table** **1**).

**Table 1:** Hybrid DNA hydrogels with dual‐network system in musculoskeletal tissue regeneration.

Dual‐network systems	Composition	Crosslinking mechanism			Dual‐network properties			Applications	Reference
		DNA network	Hybrid network	Dual network	DNA network	Hybrid network	Dual network		
Polymer composites	PEG/DNA	DNA base pairing	‐	The thiol‐ene click addition	Viscoelastic Self‐healing Injectability	Good biocompatibility and ability to load exosomes	MMP‐9 pathological clue‐triggered SCAP‐Exo controlled release	Diabetic bone regeneration	[47]
	GelMA/DNA		PhotopolymerizationChemical crosslinking	Electrostatic interactions	Rapid stress relaxation	Improved mechanical property and stability	Biomimetic natural bone ECM functions and mechanical properties	Bone defect repair	[49]
					Enhanced the emulsification efficiency and emulsion stability shape recovery ability	A highly stable emulsion template for the preparation of macroporous functional hydrogels	Macroporous hydrogel with excellent mechanical strength and effective energy dissipation facilitates tissue ingrowth	Bone defect repair	[50]
Natural material composites	SF/DNA		HRP‐mediated enzymatic crosslinking	DNA supramolecular network induce SF molecules to transition into β‐sheet conformations	Concentration‐dependent induction of β‐sheet formation in SF network	‐	Controllable surface rigidity	Articular cartilage repair	[51]
	OA/DNA		‐	Dynamic imine covalent bonds	Hydrophobic drug delivery Shear‐thinning	Tunable mechanical properties via OA oxidation degree and concentration	Shear‐thinning Injectability	Small molecule drug delivery	[52]
**Nanocomposites**	nSi/DNA		‐	Electrostatic interactions	Shear‐thinning Self‐healing	Significantly enhanced mechanical properties	DNA hydrogel with enhanced mechanical properties	Bone defect repair	[34]
	AF/Clay/DNA Dual‐Nanosystem		‐	Electrostatic interactions	Shear‐thinning Self‐healing	AF: ECM‐like fibrous structure Clay nanosheets: Osteoinductive properties	3D‐printed complex bone scaffolds	Bone defect repair	[53]

In synthetic polymer/DNA composite systems, the properties of the synthetic polymer play a crucial role in determining the suitability of the hydrogel. For instance, the PEG/DNA system utilizes click chemistry to conjugate thiolated DNA strands with PEG functionalized with 8‐arm vinyl sulfone,^[^ ^46^ ^]^ forming a 3D network via DNA base pairing.^[^ ^47^ ^]^ This approach is simple and rapid, leveraging the superior biocompatibility of PEG as the primary scaffold component for exosome loading. Additionally, the inclusion of PEG significantly enhances the stability of the hybrid DNA hydrogel, increasing its storage modulus.^[^ ^48^ ^]^ The GelMA/DNA system combines the chemically crosslinked rigidity of GelMA with the reversibly crosslinked flexibility of DNA through electrostatic interactions.^[^ ^49^, ^50^ ^]^ This approach breaks the functional limitations of individual DNA materials, imparting both mechanical strength and adaptable morphology to the hydrogel, enabling effective support and defect repair in irregular bone tissues under rigid conditions.

Natural materials incorporated into hybrid DNA hydrogels emphasize the enhancement of biological activity and the interaction between cells and the hydrogel matrix. For example, the silk fibroin (SF)/DNA system induces SF molecules to form β‐sheet structures via DNA supramolecular networks, with the surface stiffness of the hydrogel being modulated by the DNA content.^[^ ^51^ ^]^ The core design strategy in this system is to provide customizable mechanical cues that guide cellular behaviors such as proliferation and differentiation. Similarly, the oxidized alginate (OA)/DNA system utilizes the aldehyde groups of OA to form dynamic imine bonds with the amino groups on DNA, thereby constructing a dual‐network system.^[^ ^52^ ^]^ The reversible imine and hydrogen bonds endow this hydrogel with remarkable shear‐thinning characteristics, making it ideal for injection into irregular bone defects. Additionally, the hydrophobic base‐pairing regions within the DNA network offer binding sites for hydrophobic drugs, overcoming the delivery limitations typically associated with unmodified organic crosslinking materials.

The incorporation of nanomaterials further enhances the mechanical properties and functional integration of DNA hydrogels. For instance, the silicate nanodisks (nSi)/DNA system exploits the electrostatic interactions between the positively charged edges of nanosilicate sheets and the negatively charged DNA phosphate backbone, forming a physically crosslinked network.^[^ ^52^ ^]^ The inclusion of nSi increases the yield stress of the hydrogel by ≈2.5 times (at 0.5% nSi) while maintaining its elasticity and thermal stability. The introduction of amyloid fibrils (AF)/clay nanoplate double‐nanocomposites enhances the DNA network through electrostatic interactions.^[^ ^53^ ^]^ This dual‐nanocomposite system (DAC‐QK hydrogel) enables the hydrogel to exhibit ECM‐like fibrous structures and to release Si⁴^+^/Mg^2^^+^ ions in a sustained manner, promoting osteogenesis. The combination of DNA network‐induced shear‐thinning and self‐healing properties makes this system particularly suitable for 3D printing of complex bone scaffolds.

In summary, pure DNA hydrogels leverage sequence‐specific self‐assembly to form networks with inherent biocompatibility and programmability. Hybrid DNA hydrogels, however, integrate dual‐network systems that balance mechanical strength and biological activity, synergizing the strengths of multiple components. The molecular precision inherent in DNA‐based design offers a novel paradigm for personalized tissue repair, positioning DNA hydrogels as a promising platform for advanced biomedical applications.

### 2.2 DNA Component‐Loaded Hydrogel

**Figure 5:**
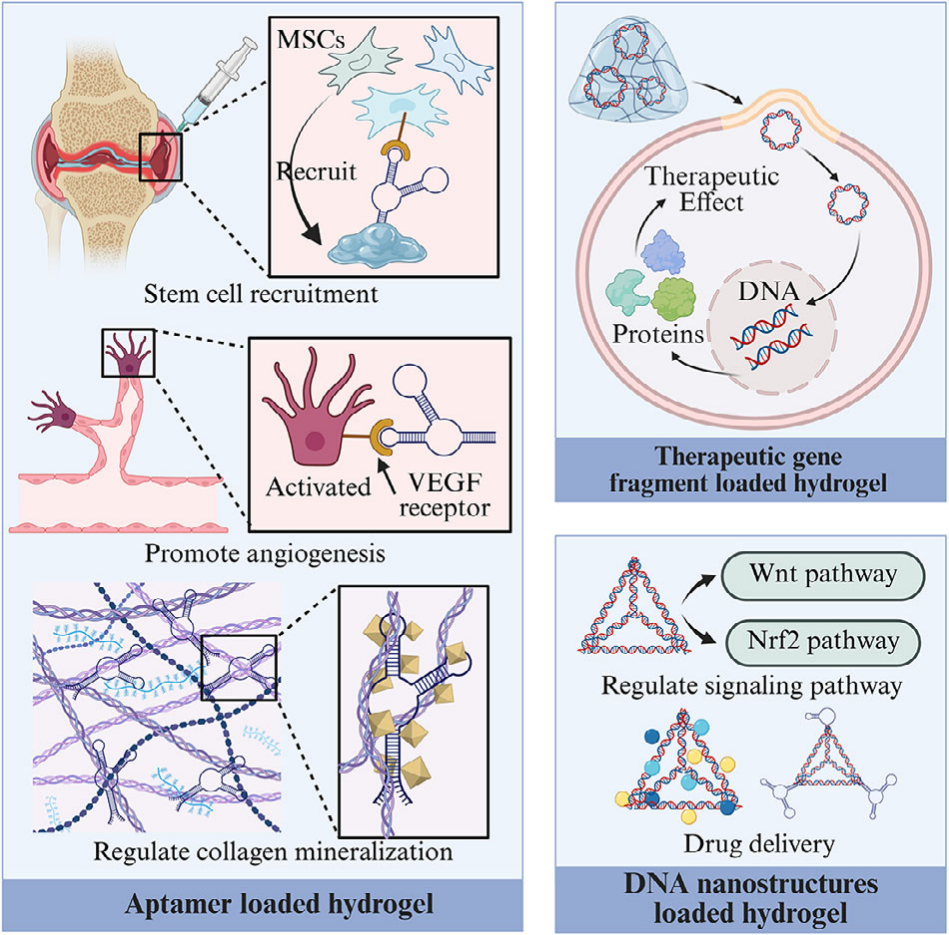
DNA component‐loaded hydrogel in musculoskeletal regeneration. Created with https://BioRender.com/.

The DNA component‐loaded hydrogel systems embed DNA functional units into non‐DNA matrices such as synthetic polymers or natural biomaterials, enabling targeted therapies through DNA‐mediated molecular recognition or gene regulation. DNA‐based components, characterized by their programmability, target specificity, and dynamic regulatory potential, represent a promising approach for skeletal muscle tissue regeneration. These components can be broadly categorized into aptamers, therapeutic DNA fragments, and DNA nanostructures, each offering distinct mechanisms of biological modulation when integrated with hydrogel matrices (**Figure** **5**).

#### 2.2.1 Aptamers Loaded Hydrogel

Aptamers are single‐stranded nucleic acid molecules, which can selectively bind to target molecules to modulate cellular behaviors and microenvironments. In skeletal muscle repair, aptamers are frequently designed to facilitate stem cell recruitment by targeting surface markers on bone marrow mesenchymal stem cells, guiding their migration to bone injury sites.^[^ ^50^ ^]^ Moreover, aptamers can mimic the action of growth factors, such as by activating vascular endothelial growth factor (VEGF) receptors to promote angiogenesis,^[^ ^54^ ^]^ or binding to collagen to regulate mineralization, thus enhancing the mechanical properties of the bone matrix.^[^ ^55^ ^]^ Aptamers can also play a role in inflammation modulation, with specific sequences capable of inhibiting neutrophil extracellular trap formation, offering novel therapeutic strategies for inflammatory bone disorders, such as rheumatoid arthritis.^[^ ^56^ ^]^

#### 2.2.2 Therapeutic DNA Fragments Loaded Hydrogel

Therapeutic DNA fragments include plasmid DNA and antisense oligonucleotides (ASOs) with the ability of gene editing. Plasmid DNA can be delivered via hydrogel‐based controlled‐release systems, enabling the localized and sustained expression of bone‐related genes, such as bone morphogenetic protein‐2 (BMP‐2), and avoiding the toxic effects associated with high doses of recombinant proteins.^[^ ^57^ ^]^ ASOs, in contrast, are primarily employed for gene silencing or exon‐skipping therapies, such as the restoration of muscle‐protective protein expression in muscular dystrophies.^[^ ^58^ ^]^ Through chemical modifications and vector optimization,^[^ ^59^ ^]^ the stability and cellular uptake of these fragments are significantly improved.

#### 2.2.3 DNA Nanostructures Loaded Hydrogel

DNA nanostructures are engineered into 3D frameworks through precise self‐assembly, such as tFNAs. These nanostructures integrate both drug delivery and signaling functionalities. tFNAs can be modified with aptamers or functional peptides to achieve multi‐target synergistic effects, such as promoting both angiogenesis and osteogenesis.^[^ ^54^ ^]^ Additionally, dynamic DNA structures, such as those based on strand displacement mechanisms, facilitate the controlled release of bioactive molecules from hydrogels.^[^ ^60^ ^]^ The small size of these structures enhances cellular uptake, while their biodegradability ensures biological safety.

In conclusion, DNA‐based components, through their molecular recognition, gene regulation, and nanotechnology‐driven approaches, impart hydrogels with enhanced intelligence and functional versatility. The direct adhesion of the hydrogel matrix to the target tissue provides enhanced targeting precision, ensuring efficient delivery to the intended site of action. Future advancements are expected to focus on the development of multifunctional DNA composites, dynamically responsive gene delivery systems, and the integration of gene editing tools such as CRISPR,^[^ ^61^ ^]^ ultimately shifting skeletal muscle regeneration from static repair mechanisms to dynamic, regulated therapeutic strategies.

## 3 Requirements for Ideal DNA‐based Hydrogels for Musculoskeletal Applications

As a versatile biomaterial for musculoskeletal tissue regeneration, DNA‐based hydrogels must exhibit tailored physicochemical and biological properties to address the dynamic demands of bone, cartilage, skeletal muscle, and tendon/ligament repair. These demands include structural support, microenvironmental regulation, and biofunctional integration, necessitating a holistic evaluation of the hydrogel's design principles. This section systematically characterizes the critical attributes of DNA‐based hydrogels that collectively define their efficacy in musculoskeletal applications, including surface characteristics, mechanical properties, biocompatibility, and bioactivity (**Table** **2**). By aligning these properties with the physiological and biomechanical requirements of target tissues, DNA‐based hydrogels can achieve precise spatiotemporal control over cellular behavior, drug delivery, and tissue remodeling, reinforcing their role as next‐generation scaffolds in this field.

**Table 2:** Requirements for Ideal DNA‐based Hydrogels for Musculoskeletal Applications.

Characteristic		Key features	Tunability	Application	Refs.
Structural Characteristics	Porous Structure	Facilitates material exchange Supports cell migration and attachment Enables drug encapsulation and sustained release	Precise control via DNA concentration, synthesis ratio and crosslinking strategy	Enabling tissue integration and microenvironment regulation Mimicking natural tissue scaffolds Facilitating controlled drug delivery	[50, 51, 62]
	Hydrophilicity and Surface Affinity	Intrinsic hydrophilicity mediated by phosphate‐derived negative charge and hydrogen‐bonded hydration networks	‐ (DNA inherent property)	Promoting bio‐interface interactions for drug delivery and tissue engineering in dynamic environments	[54, 65]
	Surface Modification	Aptamer‐based targeting for high specificity Enables stem cell recruitment and signal switching	Dynamic surface modifications via chemical modification/sequence design	Allowing adaptive responses to pathological conditions Supporting personalized therapies	[50, 56, 60]
Mechanical Properties	Dynamic Properties	Self‐healing: enables structural recovery Shear‐thinning: enhances injectability Stress relaxation: mimics ECM dynamics	Primarily attributable to the intrinsic properties of DNA strands, with limited tunability achievable through crosslinking strategies	Crucial for minimally invasive delivery, irregular defect repair and maintaining stability under load	[49, 74, 76]
	Structure Stability	Swelling: Rapid water absorption fills defects and promotes hemostasis Degradation: Sustains spatial maintenance at the defect site while synchronizing with tissue ingrowth kinetics	Controllable swelling ratio vital via double‐network structures Enhanced degradation stability via chiral engineering or disulfide crosslinking	Balancing hydrogel volume changes with the timing of drug tissue regeneration Preventing secondary damage from material failure	[49, 51, 90, 91]
	Stiffness	Influences cell differentiation through mechanical cues Provides defined mechanical support	Tunable stiffness via molecular design, crosslink density and hybrid ratio.	Adapting to tissue‐specific stiffness requirements Enhancing regeneration outcomes	[51, 94, 96]
Biocompatibility and Bioactivity	Innate Biocompatibility	Minimal inflammatory response in vivo Supports cell viability and distribution	‐ (DNA inherent property)	Reducing rejection risks in musculoskeletal implants.	[64, 76]
Bioactivity	DNA Components	Aptamers: High‐affinity targeting ability tFNAs: Modulates multiple pathways and ROS‐scavenging PDRN: Promotes angiogenesis	‐ (DNA inherent property)	Expanding therapeutic functions	[66, 100, 101, 102, 103, 104]
		DNA Hydrogels	Intrinsic antioxidant properties Supports cell adhesion Degradation metabolites aid immune modulation	Providing a multifunctional scaffold for combined drug delivery and tissue remodeling.	[105, 106, 107, 108]

### 3.1 Structural Characteristics

As tissue‐engineering implants, DNA‐based hydrogels rely on effective interactions with tissue‐contacting surfaces to initiate biological functions. DNA hydrogels, characterized by unique non‐covalent bonding between DNA strands and the intrinsic properties of the DNA material, exhibit exceptional surface characteristics. Furthermore, surface modifications of hybrid DNA hydrogels can further enhance their tissue interaction capabilities.

#### 3.1.1 Porous Structure

**Figure 6:**
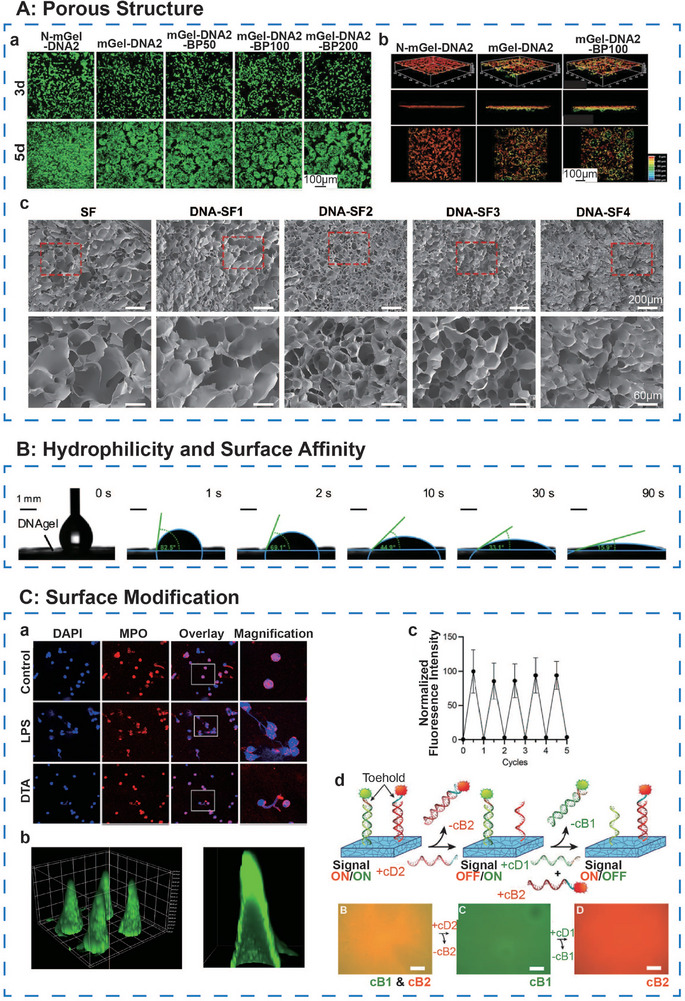
The ideal surface characteristics of DNA‐based hydrogel. A, a) Live‐dead fluorescence and b) calcein‐AM staining CLSM image of BMSCs distributed in hydrogels in different groups. Adapted under the terms of the CC BY‐NC‐ND license 4.0 from Ref. [50] Copyright 2023, Wiley‐VCH GmbH. c) SEM images of SF and dual‐network DNA‐SF hydrogels in different synthesis ratios. Reproduced with permission.^[^ ^51^ ^]^ Copyright 2024, RSC. B) Images of a water droplet and contact angles in the absorbing period by DNAgel. Adapted under the terms of the CC BY‐NC‐ND license 4.0 from Ref. [65] Copyright 2024, Springer Nature. C, a) CLSM image of neutrophils treated with LPS and DTA aptamers (red‐myeloperoxidase, blue‐DTA aptamer) (scale bar = 20 µm). b) Reconstructed spatial and cross‐section images of DTA@hMN soluble microneedles (green‐DTA aptamer). Reproduced with permission.^[^ ^56^ ^]^ Copyright 2021, ACS. c) Fluorescence intensity repeated for five cycles as different DNA handles were added to the hydrogel to turn the signal ON and OFF. d) The schematic and corresponding fluorescence images show that DNA handles allow controlled attachment and detachment in hydrogels of multiple biomolecules at customizable time points (green‐CB1, red‐CB2, and orange‐CB1&CB2). Reproduced with permission.^[^ ^60^ ^]^ Copyright 2023 Elsevier.

In DNA hydrogels, DNA chains interlink to establish a porous 3D matrix, a feature crucial for supporting various biological functions. The porous structure facilitates the transport of essential nutrients and the removal of metabolic waste, thus fostering an optimal microenvironment for tissue regeneration. Moreover, it serves as a conduit for cell migration and provides a supportive scaffold for cellular attachment. Zhou et al.^[^ ^51^ ^]^ demonstrated that the pore size in DNA‐silk fibroin hydrogels influences cellular migration; overly large pores compromise mechanical integrity, while excessively small pores obstruct normal cell passage. Furthermore, the porous configuration has been shown to exert specific biological effects. Miao et al.^[^ ^50^ ^]^ developed a double‐network macroporous hydrogel (mGel‐DNA) composed of DNA and gelatin, wherein bone marrow‐derived mesenchymal stem cells (BMSCs) infiltrated the porous structure, adhered to the pore surfaces, and promoted mineralized matrix deposition as well as osteogenic gene expression, thereby enhancing bone tissue formation (**Figure** **6**A(a,b)). Additionally, the porous architecture of these hydrogels is highly effective in drug encapsulation and controlled release. Zhang et al.^[^ ^62^ ^]^ reported a DSH‐based metformin delivery system for osteoarthritis treatment, where the 3D porous structure of DSH not only ensures efficient drug encapsulation but also prolongs MET retention in the joint cavity for up to 14 days. This extended retention prevents the rapid clearance of the drug,^[^ ^63^ ^]^ thereby reducing the frequency of injections. However, it is noteworthy that standard metrics in drug delivery, including quantitative encapsulation efficiency (EE%) and loading capacity (wt%), are rarely reported for DNA hydrogels. Future studies should prioritize including these parameters to enable cross‐platform comparisons and clinical translation potential assessment.

Achieving an optimal pore structure in DNA hydrogels necessitates precise control during their synthesis. Miao et al.^[^ ^50^ ^]^ observed an inverse relationship between pore size and DNA concentration in the initial material, indicating that higher DNA concentrations increase system viscosity, leading to heterogeneous material distribution and diminished pore connectivity (Figure 6A(c)). Moreover, varying synthesis approaches yield distinct pore sizes. For instance, DNA hydrogels produced via “heating‐cooling” processes typically form macroporous structures with pore sizes in the range of 100–200 µm, whereas those assembled from Y+L DNA monomers exhibit pore sizes determined by the center‐to‐center distance of Y‐type crosslinkers.^[^ ^64^ ^]^ Hybrid DNA hydrogels, however, require careful consideration of how the inclusion of hybrid materials influences pore size.^[^ ^42^ ^]^

Therefore, tailoring the pore structure of DNA hydrogels through precise synthesis strategies is vital to balance the biological functions and therapeutic efficacy for diverse biomedical applications.

#### 3.1.2 Hydrophilicity and Surface Affinity

Studies have demonstrated that DNA hydrogels exhibit a contact angle of ≈15–35°, indicating favorable wettability^[^ ^54^, ^65^ ^]^ (Figure 6B). This characteristic can be attributed to the inherent material properties of the DNA network, which confer high hydrophilicity and surface affinity. The negative surface charge of the DNA molecules, imparted by the phosphate groups, facilitates the adsorption of water molecules and modulates interactions with positively charged entities such as drugs or proteins. Additionally, DNA molecules can establish hydrogen bonds with water molecules, further enhancing hydrophilicity and stabilizing the hydrogel's hydrated state. These interactions promote effective engagement with cells and other biomolecules within biological environments, closely resembling the moist regenerative microenvironment found in vivo. Moreover, DNA‐based hydrogel networks form stable tissue adhesion through immediate intermolecular interactions with tissue surfaces, facilitated by hydrogen bonding and electrostatic interactions.^[^ ^65^ ^]^

Thus, the hydrophilicity and dynamic surface charge in DNA hydrogels enhance bio‐interface interactions, enabling adaptable integration with biomimetic microenvironments for advanced drug delivery and tissue engineering applications.

#### 3.1.3 Surface Modification

The internal environment during tissue reconstruction is complex, particularly at sites of tissue defects, which are often associated with intricate pathophysiological conditions. As such, biomaterials must not only possess surface properties that facilitate tissue regeneration but also interact with disease‐related molecules to enhance the local microenvironment. Moreover, these biomaterials should be compatible with other clinical therapeutic strategies to achieve optimal synergistic effects. Therefore, surface modification of DNA‐based hydrogels is a widely employed therapeutic strategy.

Nucleic acid aptamers are known for their high specificity and affinity. By modulating structures, aptamers can selectively bind to target molecules, such as cells or proteins.^[^ ^66^, ^67^ ^]^ The surface modification of DNA‐based hydrogels with aptamers allows for precise targeting of specific molecules or cells, thereby improving the specificity and effectiveness of the therapeutic approach.^[^ ^68^ ^]^

Aptamers can target disease‐related molecular biomarkers to exert therapeutic effects. Cao et al.^[^ ^56^ ^]^ developed an aptamer‐modified hyaluronic acid‐based soluble microneedle (DTA@hMN). The incorporated aptamer (DTA) can be administered via intra‐articular injection to bind with high affinity to the DEK gene,^[^ ^69^ ^]^ thereby alleviating joint inflammation.^[^ ^70^ ^]^ Researchers enhanced DTA's resistance to nucleases by modifying it with methoxy groups and idT, maintaining structural integrity for up to 72 h in 90% of mouse serum, and extending its half‐life by introducing cholesterol at the 5′ end. In vivo studies showed that DTA@hMN treatment exhibited superior efficacy to intravenous injection, highlighting its significant potential for treating rheumatoid arthritis (Figure 6C(a,b)).

Aptamers also exhibit a remarkable ability to bind with high affinity to stem cells, thereby facilitating their recruitment.^[^ ^71^ ^]^ For instance, Miao et al.^[^ ^50^ ^]^ utilized chemical anchoring of the aptamer Apt19S to modify the surface of a gelatin‐DNA double‐network hydrogel. In vitro experiments revealed a binding efficiency of 99.8% between Apt19S and BMSCs, while in vivo studies demonstrated a significant increase in BMSC accumulation and enhanced endogenous migration at the bone defect site. Importantly, the nanoscale size of the aptamer ensured that its modification of the hydrogel surface did not alter the macroscopic appearance or mechanical properties of the material. These results suggest that Apt19S facilitates inward cell migration and promotes bone tissue formation by recruiting stem cells, thereby contributing to the hydrogel's regenerative efficacy.

In addition to using aptamers for surface modification of hydrogels, Fumasi et al.^[^ ^60^ ^]^ introduced reversible DNA handles to modify hyaluronic acid hydrogels. These single‐stranded DNA handles were covalently attached to the HA hydrogel surface via thiol‐ene coupling and subsequently hybridized with the peptide‐DNA conjugate through complementary base pairing. To achieve selective addition and removal of multiple biomolecular signals over several cycles, the authors incorporated a single‐stranded DNA “toehold” within the peptide‐DNA conjugate.^[^ ^72^ ^]^ By introducing a fully complementary strand, the toehold was bound, triggering branch migration and replacing the original hybridized strand, thereby facilitating the switching and replacement of biological signals. The system demonstrated stable signal cycling for at least five rounds without attenuation and enabled the orthogonal addition and removal of up to three distinct signals concurrently (Figure 6C(c,d)). The researchers applied this approach to sequentially present the cell adhesion peptide RGD and the osteogenic growth peptide (OGP), observing modulated cellular behavior over a 14‐day in vitro culture period. This method offers a robust platform for optimizing the spatial and temporal presentation of bioactive signals.

These advancements highlight the importance of molecular precision in DNA hydrogels, which enables multifunctional platforms to address complex tissue repair challenges and personalized regenerative therapies.

Current researches demonstrate that the ideal structural characteristics of DNA‐based hydrogels confer three core advantages in tissue repair:

1. Controllable porous structures facilitate cell migration and optimize drug sustained release by providing cellular migration channels and drug reservoirs.
2. Exceptional surface affinity enhances cell attachment and stabilizes drug diffusion through maintaining a hydrated microenvironment and adsorbing positively charged biomolecules via negative surface charge.
3. Precision surface modifications dynamically regulate local biological signals through molecular recognition capabilities, such as aptamer‐target binding.

Ideal DNA‐based hydrogels ought to intelligently adjust their surface physicochemical properties, like pore size and charge distribution, in response to microenvironmental cues such as inflammatory signals and mechanical loads. Such innovations could bridge the gap between static material design and dynamic healing demands, ultimately advancing personalized regenerative medicine.

### 3.2 Mechanical Properties

The mechanical behavior of DNA‐based hydrogels is pivotal in determining their suitability for musculoskeletal tissue regeneration, where dynamic biomechanical forces and tissue‐specific stiffness requirements must be harmonized. DNA hydrogels, leveraging their programmable molecular architecture and reversible non‐covalent interactions, exhibit unique mechanical adaptability. This section explores how the molecular design and mechanical performance of DNA hydrogels address the physiological and clinical challenges of musculoskeletal regeneration.

#### 3.2.1 Dynamic Properties—Self‐Healing, Shear‐Thinning, and Stress‐Relaxing Behavior

**Figure 7:**
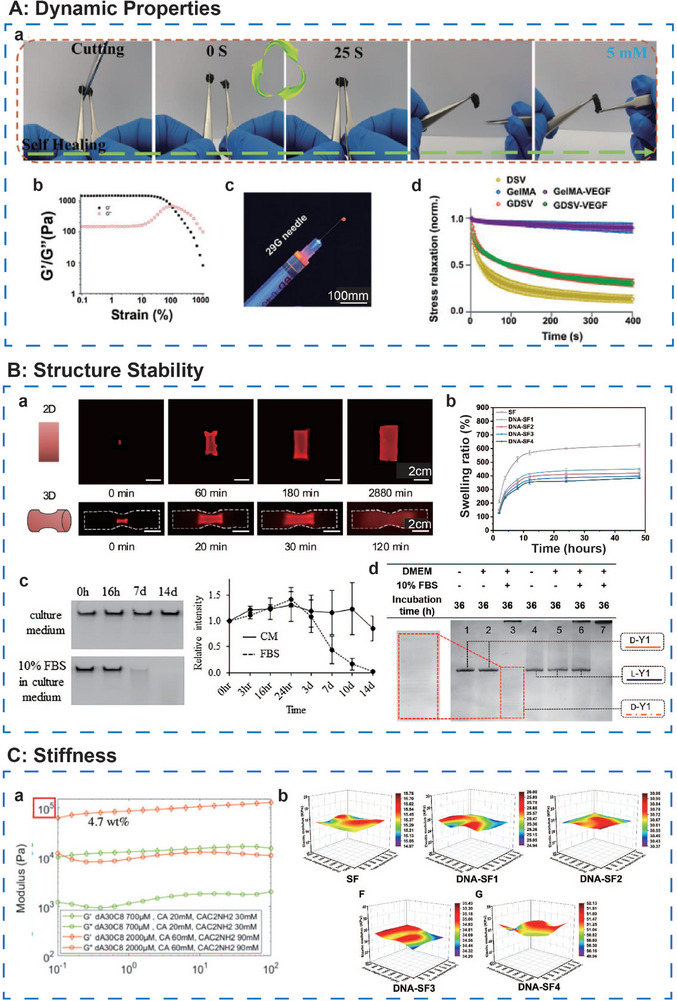
Ideal mechanical properties of DNA‐based hydrogels. A, a) Self‐healing properties of the X+Y DNA hydrogel. Reproduced with permission.^[^ ^40^ ^]^ Copyright 2021, Wiley. b) Frequency sweep test and c) injectability test of BMSCs‐laden DNA supramolecular hydrogel. Reproduced with permission.^[^ ^76^ ^]^ Copyright 2021, Wiley. d) Normalized stress relaxation in different hydrogel groups. Adapted under the terms of the CC BY‐NC‐ND license 4.0 from Ref. [49] Copyright 2024, KeAi Publishing. B, a) Images of DNA gel in 2D and 3D shape during swelling in different time periods. Adapted under the terms of the CC BY‐NC‐ND license 4.0 from Ref. [65] Copyright 2024, Springer Nature. b) The swelling ratio of SF and dual‐network DNA‐SF hydrogels in different synthesis ratios in PBS at 37 °C. Reproduced with permission.^[^ ^51^ ^]^ Copyright 2024, RSC. c) Images of denaturing gel electrophoresis (left) and degradation kinetics of DNA hydrogel incubated in different medium. Adapted under the terms of the CC BY‐NC‐ND license 4.0 from Ref. [64] Copyright 2023, PNAS. d) 20% denatured PAGE characterization of D‐DNA and L‐DNA incubated in different medium. Reproduced with permission.^[^ ^90^ ^]^ Copyright 2022, Wiley. C, a) The sidechain of the cyanuric acid effectively tunes the mechanical properties of dA/CA hydrogels. Adapted under the terms of the CC BY‐NC‐ND license 4.0 from Ref. [96] Copyright 2024, Wiley. b) 3D distribution map of the elastic modulus and rheological curves of SF and dual‐network DNA‐SF hydrogels in different synthesis ratios. Reproduced with permission.^[^ ^51^ ^]^ Copyright 2024, RSC.

DNA hydrogels are primarily composed of ssDNA chains, which are interconnected through base pairings via hydrogen bonds, resulting in a self‐assembled structure.^[^ ^17^ ^]^ This intrinsic design enables DNA hydrogels to undergo rapid and spontaneous gelation without the need for external stimuli (**Figure** **7**A(a)). The non‐covalent interactions among the hydrogen bonds confer reversible dynamic mechanical properties to the hydrogel. When subjected to larger external forces, these hydrogen bonds may break; however, the DNA chains can spontaneously reassemble and reconnect through base pairing, thereby restoring the original structure and functionality.^[^ ^73^ ^]^ This dynamic self‐healing capability enables DNA hydrogels to adapt to irregular tissue defects in regenerative medicine, ensuring stable biological function even under prolonged load or mechanical stress.^[^ ^74^ ^]^ Moreover, in drug delivery systems, self‐healing DNA hydrogels can efficiently encapsulate therapeutic agents and control their release, addressing challenges associated with drug leakage or diminished efficacy due to mechanical damage.^[^ ^75^ ^]^

The self‐healing capability of DNA hydrogels also imparts notable shear‐thinning behavior. Yan et al.^[^ ^76^ ^]^ synthesized Y+L‐DNA supramolecular hydrogels and characterized their rheological properties. Their findings revealed that, over a frequency range of 0.1–100 Hz in time sweep mode, the shear storage modulus (G′) consistently exceeded the shear loss modulus (G″), indicating that the hydrogel predominantly exhibits elastic rather than viscous behavior. This elasticity enables the hydrogel to maintain its structure under external stress while demonstrating excellent shape‐memory properties and enhanced load‐bearing capacity. Upon increasing shear stress, both G′ and G″ remained constant within a low shear strain range. However, at higher shear strains, G″ began to increase while G′ decreased, with the two moduli intersecting at a shear strain of 105%. This behavior suggests that, under specific shear stresses, the DNA supramolecular hydrogel transitions to a more fluid‐like state, exhibiting shear‐thinning behavior and enhanced injectability (Figure 7A(b,c)). A similar pattern was observed in DNA strand‐reassembled hydrogel formed via a “heating‐cooling” process,^[^ ^54^ ^]^ suggesting that shear‐thinning is a universal feature of DNA hydrogels, regardless of the fabrication method. These biomechanical properties underscore the potential of DNA hydrogels for efficient and stable delivery within biological systems, facilitating the development of more precise, minimally invasive drug delivery strategies.

Dynamic chemistry studies have demonstrated that non‐covalent interactions enable hydrogels to achieve rapid stress relaxation.^[^ ^77^ ^]^ DNA hydrogels, with their abundant hydrogen bonds, represent an ideal candidate for the design of stress‐relaxing materials.^[^ ^78^ ^]^ Despite this potential, research on the stress relaxation properties of DNA hydrogels remains sparse. Li et al.^[^ ^49^ ^]^ reported that DNA hydrogels achieved stress relaxation within 29 s, whereas GelMA‐DNA dual‐crosslinked hydrogels required 100 seconds for the same process (Figure 7A(d)). The authors suggested that the relaxation behavior is closely linked to the crosslinking mechanism, with physically crosslinked DNA networks facilitating faster stress relaxation, while covalently crosslinked GelMA networks partially limit this process. This work underscores the promise of physically crosslinked DNA hydrogels in the development of stress‐relaxing materials, which could better replicate the mechanical properties of the natural ECM and subsequently influence cellular behavior.^[^ ^79^, ^80^ ^]^

In all, with their unique dynamic properties, DNA hydrogels not only resolve the contradictions in traditional hydrogels about mechanical compatibility, injectability, and dynamic responsiveness, but also lay the technical foundation for developing intelligent responsive tissue engineering scaffolds.

#### 3.2.2 Structure Stability—Swelling and Degradation

Despite the remarkable properties of DNA‐based hydrogels under ideal conditions, their practical application in vivo is often constrained by challenges in controlling the temporal changes in their shape after implantation.^[^ ^81^ ^]^ In the water‐rich in vivo environment, DNA‐based hydrogels undergo two distinct phases of volume change: swelling and degradation. Initially, the hydrogel swells as solvent molecules penetrate the matrix and interact with the polymer chains, leading to an expansion in volume. This swelling continues until equilibrium is reached, at which point the hydrogel can perform its intended physiological functions. Over time, intracellular proteins and enzymes begin to degrade the polymer network, resulting in the loss of elasticity and the transition from a gel state to a sol state—a process known as degradation. For DNA‐based hydrogels to exert controlled therapeutic effects in musculoskeletal tissue regeneration, careful design and maintenance of their structural stability are crucial.

##### Swelling

DNA hydrogel networks, with their rough surface and highly porous structure, exhibit a pronounced hydrophilic nature, enabling rapid water absorption and subsequent swelling upon exposure to environmental moisture^[^ ^65^ ^]^ (Figure 7B(a)). This characteristic facilitates the hydrogel's swift absorption of blood and its expansion to fill defect sites, exerting localized pressure on surrounding tissues within confined spaces. Such pressure induces vasoconstriction and slows blood flow, thus promoting effective hemostasis in acute injury scenarios.^[^ ^82^ ^]^ Also, the hydrogel's rapid swelling allows it to adapt quickly to the irregular geometry of tissue defects. The high swelling ratio of DNA hydrogel also enables efficient drug encapsulation and controlled release.^[^ ^83^ ^]^

Despite all the advantages of the high swelling property, it is necessary to control the swelling ratio of DNA‐based hydrogels within an optimal range. For example, in cartilage regeneration, the hydrogel must mimic the properties of articular cartilage, including joint lubrication, swelling, and viscoelasticity, which requires controlling the swelling degree to a certain amount.^[^ ^84^ ^]^ Excessive swelling in clinical settings can undermine the mechanical properties and dimensional stability of the hydrogel, leading to unpredictable outcomes.^[^ ^85^ ^]^ Moreover, swelling‐induced compression of adjacent organs or tissues can significantly impair the regenerative potential of the material.^[^ ^86^ ^]^ Therefore, to ensure long‐term stability within musculoskeletal tissues and preserve the hydrogel's original size and shape, the swelling behavior must reach an equilibrium state, maintaining the hydrogel's initial form and inherent properties in the surrounding tissue fluid.^[^ ^87^ ^]^

Previous studies have shown that incorporating a double‐network structure can modulate the swelling behavior of hydrogels.^[^ ^88^, ^89^ ^]^ For example, Li et al.^[^ ^49^ ^]^ demonstrated that the inclusion of GelMA in a double‐network system prolonged the time required for the hydrogel to reach swelling equilibrium, increasing from 8 h in pure DNA networks to 12 h in the double‐network configuration. Remarkably, despite the high intrinsic swelling capacity of the DNA network, its crosslinking with the similarly hydrophilic GelMA network resulted in a nearly 20% reduction in the swelling rate. This swelling regulation capability is also evident in SF‐DNA double‐network hydrogels^[^ ^51^ ^]^ (Figure 7B(b)). Therefore, it can be inferred that this regulatory effect is mutual, resulting from the physical crosslinking between the two networks.

##### Degradation

The degradation behavior of DNA‐based hydrogels is critical not only for maintaining the material's structural integrity and volume but also for influencing its functional performance. In regenerative medicine, the degradation rate of hydrogels must be carefully calibrated to match the rate of tissue ingrowth, ensuring the creation of an optimal microenvironment for cell migration and tissue remodeling. However, unmodified DNA hydrogel networks often degrade too quickly for applications that require sustained biological effects. For instance, the Y+L DNA hydrogel developed by Athanasiadou et al.^[^ ^64^ ^]^ demonstrated significant degradation within just 16 h in cell culture medium and exhibited a half‐life of only 6.6 days in 10% serum (Figure 7B(c)). In an in vivo bone defect model, the rapid degradation of the DNA hydrogel resulted in transient osteoinductive effects, which were substantially less effective than those of autologous bone controls.

To address these limitations, researchers have explored various strategies to extend the degradation time of DNA‐based hydrogels. For example, Han et al.^[^ ^54^ ^]^ incorporated rabbit actin at a final concentration of 300 µg mL^−1^ during the physical blending of pure DNA hydrogels. After three weeks of incubation in 10% fetal bovine serum (FBS), the hydrogel retained 90% of its original content, making it more suitable for long‐term bone repair applications. In an in vivo bone defect model, histological analysis at 4–8 weeks post‐implantation revealed a hybrid layer of newly formed bone and material interlocked within the scaffold pores, contributing to a stable and durable structure. Additionally, Yang et al.^[^ ^90^ ^]^ introduced a novel L‐DNA supramolecular hydrogel, where the single strands are enantiomeric but share the same sequence as D‐DNA. L‐DNA hydrogels exhibit similar network topology and excellent properties such as shear‐thinning, self‐healing, and thermal responsiveness. The mirror‐image double helix formed by the enantiomeric deoxyribose provides increased resistance to nuclease degradation, with structural stability maintained for up to one month in FBS (Figure 7B(d)). Further, Du et al.^[^ ^91^ ^]^ enhanced the stability of pure DNA hydrogels by incorporating thiol groups, enabling the formation of disulfide bonds in the presence of oxidizing agents. This conversion from a supramolecular to a covalent network significantly enhanced the hydrogel's mechanical strength and stability. Over time, while the self‐healing and shear‐thinning properties diminished, the stability and mechanical properties of the hydrogel improved. In vitro studies demonstrated that 293T cells were distributed in 3D within the hydrogel, with the increased mechanical strength delaying cellular settling, suggesting that this system could serve as a platform for simulating complex in vivo physiological processes, such as bone formation within a hardening extracellular matrix (ECM) environment.

In summary, these strategies highlight the potential for DNA‐based hydrogels to provide necessary structural stability, great adaptability, and prolonged therapeutic effects for tissue regeneration applications. Current research has achieved regulation of the stability properties through the introduction of dual‐network structures. This “adversarial synergy” not only preserves the functional advantages of individual materials but also suppresses the negative effects of volume changes through mutual constraints from network crosslinking.

#### 3.2.3 Stiffness

The ability of cells to sense and respond to the stiffness of the local matrix is crucial for their differentiation and regenerative processes. Stem cells involved in tissue regeneration interpret mechanical signals and characteristics within their microenvironment through mechanosensing and mechanotransduction, which in turn regulate their proliferation, self‐renewal, and differentiation into specific cell types.^[^ ^92^, ^93^ ^]^ Furthermore, the regeneration of musculoskeletal tissues is influenced by the varying mechanical forces and movement states at different anatomical sites, each of which imposes distinct stiffness requirements on biomaterials.^[^ ^94^, ^95^ ^]^ Consequently, precise control over the stiffness of DNA‐based hydrogels is essential for adapting these materials to meet the specific needs of different regenerative contexts.

However, the shear‐thinning characteristics of DNA hydrogels result in relatively low mechanical stiffness, with a storage modulus typically ranging from 10^1^ to 10^3^ Pa.^[^ ^64^ ^]^ This limited stiffness restricts their applicability in environments requiring stiffer materials, such as bone, tendon, and ligament tissues. Thanks to the programmability of DNA molecular design and sequence, DNA‐based hydrogels offer tunable stiffness, enabling them to be finely adjusted for optimal performance in the complex and dynamic environment of musculoskeletal regeneration.

To address this issue, Lachance‐Brais et al.^[^ ^96^ ^]^ introduced a novel approach by utilizing the dA/CA motif, a noncanonical nucleic acid structure formed through the interaction of deoxyadenine (dA) and cyanuric acid (CA), a small and nontoxic molecule. This interaction results in the formation of a parallel DNA triple helix, where the three faces of cyanuric acid mimic thymine, bridging the adenine residues of poly‐dA chains and inducing the formation of a supramolecular polymer. The system was further modularized, with the mechanical modulus being tunable through adjustments to the complementary protruding ends of DNA or the side chains of cyanuric acid. The results demonstrated that this unmodified DNA hydrogel achieved a significantly higher storage modulus of up to 10^5^ Pa at 4.7 wt.%, which is two orders of magnitude greater than typical DNA hydrogels (Figure 7C(a)). Importantly, this modification preserved the hydrogel's inherent editability and rapid self‐healing properties, allowing for integration with other DNA components and facilitating applications in injectable therapeutic drug delivery.

Additionally, by adjusting the synthetic ratio, hybrid DNA hydrogels can modulate the extent of interaction between the hybrid dual‐networks to achieve tunable mechanical stiffness. Jiang et al.^[^ ^94^ ^]^ developed DNA‐crosslinked polyacrylamide gels, where DNA acts as a crosslinker within the polymer matrix. By varying the length of the DNA crosslinker, monomer concentration, and crosslinking density, they were able to modulate the stiffness of the hybrid gels within a range from several hundred Pa to 30 kPa. Furthermore, they conjugated the DNA gel with ECM proteins to promote neuronal cell attachment and observed biologically relevant effects driven by mechanical cues. This research provides evidence of mechanosensitivity in neurogenesis. Similarly, Su et al.^[^ ^51^ ^]^ developed a silk fibroin‐DNA hydrogel system with tunable surface rigidity. Their studies revealed that the DNA supramolecular network induced a conformational shift of SF molecules from irregular coil structures to β‐sheet formations. Additionally, the DNA‐SF hydrogel demonstrated an increase in surface stiffness with higher β‐sheet content, which could be precisely controlled by adjusting the DNA‐to‐SF ratio (Figure 7C(b)). Importantly, DNA‐SF hydrogels directly influenced cellular behavior through mechanical stimulation, with the effects closely correlating to surface stiffness. The results indicated that a moderate DNA content (resulting in a surface stiffness of 30.21 kPa) provided optimal mechanical conditions, supporting cell proliferation and migration, promoting chondrogenic differentiation of BMSCs, and exhibiting sustained biological efficacy during long‐term degradation. This makes the hydrogel particularly well‐suited for the extended healing of cartilage defects.

Through molecular design, DNA hydrogels have achieved tunable stiffness ranging from hundreds of pascals to tens of kilopascals, preliminarily establishing a compatible framework between mechanical compatibility and biological functionality. Future studies could further explore dynamic coupling mechanisms of stiffness parameters with other microenvironmental variables, such as bioactive molecule release and dynamic load variations, to better replicate the regenerative microenvironment of natural tissues.

The three mechanical properties of DNA‐based hydrogels correspond to distinct core advantages:

1. Dynamic properties ensure structural recovery and injectability in dynamic environments through reversible hydrogen bonding, while mitigating inflammation risks caused by mechanical stress.
2. Structural stability balances hydrogel volume changes with the timing of drug sustained‐release or tissue regeneration, preventing secondary damage from material failure.
3. Tunable stiffness directly guides stem cell differentiation through mechanical cues, enabling controlled regenerative outcomes.

While current studies have preliminarily addressed the challenges of mechanical compatibility, future research on ideal DNA‐based hydrogels should focus on adaptive mechanisms. For example, adjusting mechanical properties over time or integrating macroscale mechanical support with microscale cellular mechanical microenvironments. This hierarchical mechanical design could better mimic the natural repair processes of human tissues, thereby accelerating the clinical translation of self‐adaptive biomaterials for real‐world medical applications.

### 3.3 Biocompatibility and Bioactivity

As an implantable biomaterial, the biocompatibility of DNA‐based hydrogels is a critical prerequisite for their clinical application, while their bioactivity directly governs tissue regeneration efficacy. This section addresses the biocompatibility characteristics and bioactivity modulation mechanisms of DNA hydrogels and other DNA components, examining both aspects in detail.

#### 3.3.1 Innate Biocompatibility

As a natural biopolymer, DNA exhibits a high degree of structural homology between its fundamental units, deoxyribonucleotides, and endogenous molecules in the human body, thereby conferring inherent biocompatibility to DNA‐based hydrogels, including both the hydrogel network and DNA components.

**Figure 8:**
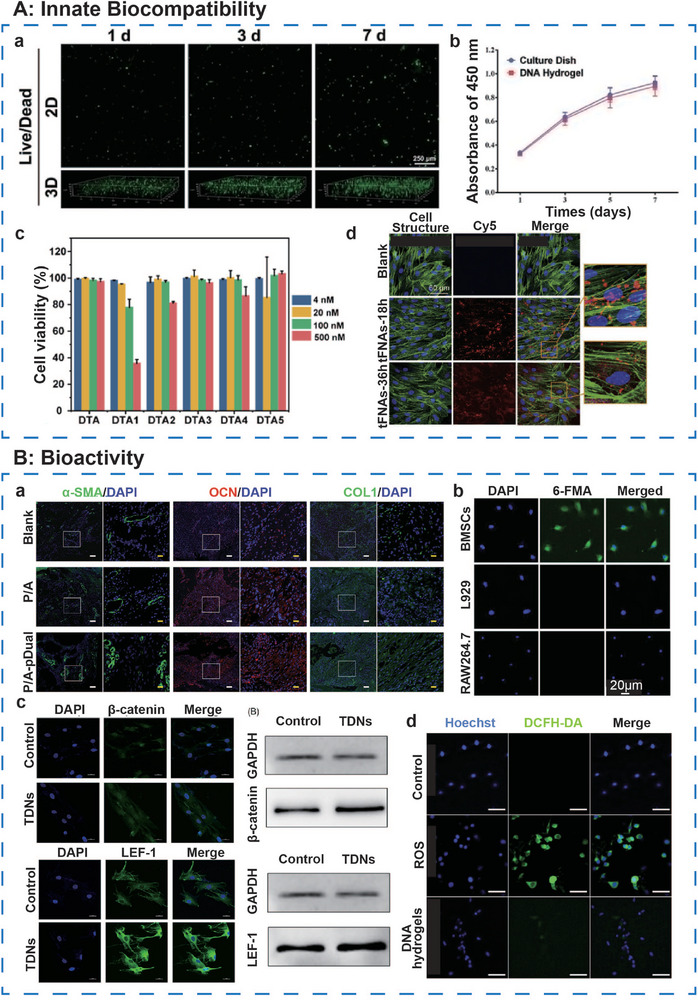
Ideal biocompatibility and bioactivity of DNA‐based hydrogels. A, a) 3D distribution of BMSCs and b) cell viability cultured in DNA hydrogel. Reproduced with permission.^[^ ^76^ ^]^ Copyright 2021, Wiley. c) Cell viability after incubation with DTA aptamers. Reproduced with permission.^[^ ^56^ ^]^ Copyright 2021, ACS. d) Fluorescence images of tFNAs’ cellular uptake. Reproduced with permission.^[^ ^109^ ^]^ Copyright 2025, Elsevier. B, a) Fluorescence images showing the expression of angiogenic markers and osteogenic markers in the defect area. Reproduced with permission.^[^ ^110^ ^]^ Copyright 2022, Wiley. b) Fluorescence images of different cells after incubation with 6‐FAM‐labeled Apt19S, respectively. Adapted under the terms of the CC BY‐NC‐ND license 4.0 from Ref.[50] Copyright 2023, Wiley‐VCH GmbH. c) Fluorescence images of β‐catenin and LEF1 protein. Reproduced with permission.^[^ ^111^ ^]^ Copyright 2019, Wiley. d) Fluorescence images of DCFH‐DA and Hoechst‐stained cells in different groups. Reproduced with permission.^[^ ^106^ ^]^ Copyright 2022, Wiley.

In vitro cellular assays have demonstrated that pure DNA hydrogels facilitate the adhesion and proliferation of BMSCs. After 7 days of culture without external induction, over 99% of encapsulated BMSCs remained viable, maintaining a uniform 3D distribution^[^ ^76^ ^]^ (**Figure** **8**A(a,b)). In subcutaneous implantation models, DNA hydrogels induced no significant inflammatory response within 24‐ and 72‐h post‐surgery, with only minimal inflammatory cell accumulation observed. Similarly, in a rat calvarial defect model, no inflammatory signs were detected over the relatively short‐term period of 28 days following implantation.^[^ ^64^ ^]^ This positive result indicates an absence of acute immune rejection and contributes to the evidence for short‐to‐mid‐term biocompatibility of DNA hydrogel. However, specific data evaluating potential chronic immune reactions or the long‐term immunogenicity profile (>28 days) of this material remain limited and represent a key area recommended for further investigation.

Aptamers, as nanoscale DNA components, are efficiently absorbed into the bloodstream. Routine hematological tests have shown that aptamers do not alter the number or concentration of blood cells in mice (Figure 8A(c)), indicating favorable biocompatibility.^[^ ^56^ ^]^ Tetrahedral DNA frameworks, influenced by caveolin‐mediated endocytosis, exhibit effective membrane penetration capabilities. Upon interacting with the cell membrane and undergoing internalization, tFNAs enter cells without eliciting immune responses, thereby demonstrating superior biocompatibility^[^ ^97^ ^]^ (Figure 8A(d)).

#### 3.3.2 Bioactivity

##### DNA Components

DNA components exhibit diverse bioactive properties within biomedical applications, with their mechanisms of action closely tied to their structural characteristics. Gene delivery vectors, such as plasmid DNA and antisense oligonucleotides, facilitate targeted gene regulation through precise sequence encoding. Plasmid DNA reprograms cellular function by inducing exogenous gene expression,^[^ ^98^ ^]^ (Figure 8B(a)), while antisense oligonucleotides regulate gene expression by complementary pairing with target nucleotides.^[^ ^99^ ^]^ The bioactivity of these components is highly dependent on sequence specificity and chemical modifications. Aptamers, as single‐stranded nucleic acids, recognize target proteins with high affinity through distinct 3D conformations, acting as molecular probes in disease diagnosis and targeted therapy^[^ ^66^ ^]^ (Figure 8B(b)).

In contrast to traditional linear nucleic acid structures, tFNAs, formed through self‐assembly, possess groundbreaking biological functions due to their spatial configuration, enabling interaction with multiple signaling pathways to exert regulatory effects. tFNAs promote cell proliferation and migration by modulating key pathways, such as Wnt/β‐catenin,^[^ ^100^ ^]^ while their anti‐inflammatory and antioxidant activities are mediated through the direct scavenging of reactive oxygen species (ROS) and activation of the Nrf2/HO‐1 signaling pathway.^[^ ^101^ ^]^ In tissue engineering, tFNAs have been shown to induce osteogenic and chondrogenic differentiation in stem cells (Figure 8B(c)), positioning them as a prominent topic in the field of tissue regeneration.^[^ ^102^, ^103^ ^]^

Furthermore, salmon‐derived polydeoxyribonucleotide (PDRN), a polydeoxyribonucleotide complex, functions through a dual mechanism: it enhances VEGF‐mediated angiogenesis via adenosine A2A receptor activation and promotes tissue repair by supplying nucleotides through a “rescue pathway”.^[^ ^104^ ^]^

##### DNA Hydrogels

The biological functions of DNA hydrogels are inherently constrained by their constituent materials. Existing studies have demonstrated that DNA strand breakage exhibits scavenging activity toward hydroxyl radicals (⋅OH).^[^ ^105^ ^]^ Therefore, the intrinsic antioxidant properties of DNA molecules in DNA hydrogels enable effective scavenging of ROS, which has been shown to alleviate oxidative stress in chronic wounds^[^ ^106^ ^]^ (Figure 8B(d)).

Although DNA hydrogels themselves do not often directly serve as the primary bioactive agents, their unique physicochemical properties and degradation characteristics confer a range of biological functions. As multifunctional biomaterials, the bioactivity of DNA hydrogels is derived from their distinctive chemical composition and dynamic degradation properties. While DNA is not the core functional molecule, the negative charge of the phosphate backbone facilitates calcium ion chelation, promoting hydroxyapatite mineralization and providing a biomimetic template for bone regeneration.^[^ ^107^ ^]^ Additionally, the 3D network structure of DNA hydrogels supports cell adhesion and migration,^[^ ^108^ ^]^ and their enzymatic degradation produces functional metabolites, such as phosphate and adenine, which participate in immune modulation and tissue repair.

Despite that DNA hydrogels exhibit limited direct bioactivity, their integration with active DNA components and bioactive nucleotide complexes can significantly expand their application boundaries in regenerative medicine. In summary, DNA‐based hydrogels leverage the biocompatibility of nucleotide units with endogenous molecules and diverse bioactive properties to establish a versatile platform that integrates biosafety with microenvironment‐modulating capabilities.

## 4 Applications of DNA‐Based Hydrogels in Musculoskeletal Tissue Regeneration

Musculoskeletal tissue regeneration is a multifaceted process governed by intricate biological cascades and microenvironmental cues, yet often hindered by structural complexity, pathological disruptions, and intrinsic healing limitations. Bone, cartilage, skeletal muscle, tendons, and ligaments each exhibit unique regenerative pathways shaped by their distinct biomechanical demands and cellular dynamics. These biological intricacies underscore the need for biomaterial strategies that dynamically adapt to tissue‐specific regenerative timelines and molecular signaling. This section delineates the biological foundations of musculoskeletal tissue regeneration, and emphasizes how the molecular design principles of DNA‐based hydrogels align with the physiological and pathological demands, thereby bridging material innovation with regenerative biology.

### 4.1 Bone Regeneration

Bone tissue repair in clinical practice predominantly relies on autologous or allogeneic bone grafting.^[^ ^112^ ^]^ However, the clinical applicability of bone grafts is often constrained by challenges such as donor site morbidity, limited graft availability, and the potential for infection.^[^ ^113^ ^]^ These limitations have driven significant interest in the development of biomaterials that can promote bone regeneration, offering advantages such as low risk, ease of procurement, and efficient osteoinductive properties. This section explores the various design strategies employed in the development of DNA‐based hydrogels and examines their applications in bone tissue engineering and repair.

#### 4.1.1 Biology of Bone Tissue Regeneration

Bone is a dynamic mineralized connective tissue characterized by its hierarchical structure, consisting of cortical (compact) and cancellous (trabecular) bone. Cortical bone provides structural rigidity with a Young's modulus of 17–20 GPa, while cancellous bone exhibits porosity (50–90%) to absorb compressive loads.^[^ ^114^ ^]^ The extracellular matrix (ECM) comprises 60% inorganic hydroxyapatite and 30% collagen type I, enabling both compressive strength and fracture toughness.^[^ ^115^ ^]^

Bone regeneration is a highly orchestrated process involving sequential phases of inflammation, repair, and remodeling. Following injury, the early inflammatory phase (days 0–7) begins. The hematoma forms a provisional matrix rich in fibrin and platelets, which recruits inflammatory cells through chemotactic signals such as C‐X‐C Motif Chemokine Ligand 12 (CXCL12) and TGF‐β1. Meanwhile, macrophages polarized to the M1 phenotype dominate the early inflammatory phase, secreting pro‐inflammatory cytokines (IL‐1β, TNF‐α) to clear debris while activating MSCs via the NF‐κB pathway.^[^ ^116^ ^]^ Then, during the reparative phase (days 7–28), MSCs differentiate into osteoblasts under the guidance of BMP‐2 and Wnt/β‐catenin signaling, depositing a collagen‐rich osteoid matrix that undergoes mineralization through alkaline phosphatase (ALP)‐mediated phosphate release.^[^ ^117^ ^]^ When entering the later stages of fracture repair (after 6 weeks), the regeneration of bone tissue manifests as bone remodeling, and the chemical composition of the bone undergoes continuous turnover through this complex process.^[^ ^118^ ^]^ Osteoclasts, derived from monocyte precursors via RANKL/OPG signaling, resorb necrotic bone while osteocytes orchestrate mechanotransduction through sclerostin inhibition and connexin 43‐mediated intercellular communication.^[^ ^115^ ^]^

However, pathological conditions disrupt this cascade. Chronic conditions like osteoporosis disrupt this balance, as senescent MSCs exhibit reduced β1‐integrin expression, impairing their mechanosensitivity and osteogenic potential.^[^ ^119^ ^]^ Diabetic bone defects present additional challenges due to AGE‐RAGE axis activation, which suppresses autophagy and induces pyroptosis in osteoblasts.^[^ ^120^ ^]^

#### 4.1.2 Osteoinductive Properties of DNA‐Based Hydrogels

This section explores the interplay between DNA's molecular architecture, mineralization guidance, and osteoinductive potential, highlighting recent advances in DNA hydrogel design that bridge biomimetic mechanics and regenerative bioactivity for bone repair.

##### DNA Components: Multifaceted Roles in Mineralization and Osteoinduction

DNA strands exhibit favorable inorganic cation coordination properties, particularly with ions such as Ca^2+^,^[^ ^121^ ^]^ facilitating the templating of inorganic material growth. Also, DNA nanotechnology offers the advantage of precise control over the morphology of nanostructures,^[^ ^122^ ^]^ which provides customized templates for mineralization. Therefore, DNA nanostructures have garnered widespread attention for mimicking the natural process of structural mineralization.^[^ ^123^ ^]^

**Figure 9:**
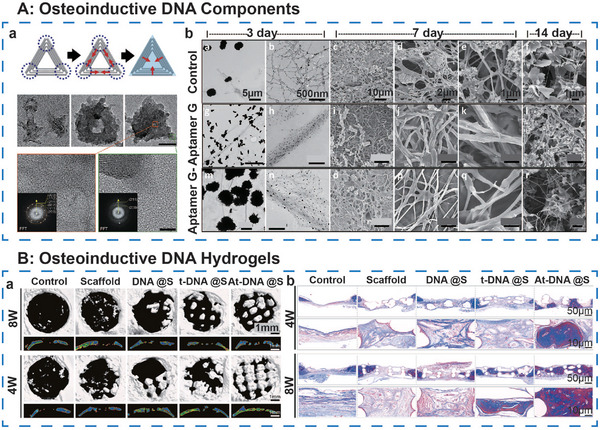
Osteoinductive Properties of DNA‐Based Hydrogels. A, a) TEM and SEM images of calcium phosphate mineralized collagen hydrogels with or without DNA aptamers in 3, 7, and 14 days. Adapted under the terms of the CC BY‐NC‐ND license 4.0 from Ref. [55] Copyright 2024, Elsevier. b) TEM images of triangular DNA origami‐CaP nanocrystals showing the crystallization trend from tips to center (Scale bars= 50 nm‐top; 5 nm‐bottom). Adapted under the terms of the CC BY‐NC‐ND license 4.0 from Ref. [124] Copyright 2020, Elsevier. B, a) Micro‐CT images with cross‐sectional and longitudinal views of cranial defects treated with different hydrogel groups. b) Masson's staining images of cranial defects after implanting with different hydrogel groups for 4 and 8 weeks. Adapted under the terms of the CC BY‐NC‐ND license 4.0 from Ref. [54] Copyright 2024, KeAi Publishing.

Liu et al.^[^ ^124^ ^]^ demonstrated a DNA framework‐based approach to template calcium phosphate crystallization. Their study revealed that electrostatic interactions within the DNA framework direct CaP crystallization, exhibiting versatility across different DNA nanostructures. The crystallization direction of CaP was determined by the geometric features of the DNA structures, rather than the preferred theoretical *c*‐axis direction (**Figure** **9**A(a)). Furthermore, the CaP shell formed around the DNA framework served as a protective barrier, enhancing the intracellular delivery of functional nucleic acids. This observation underscores the ability of DNA frameworks to influence CaP mineralization at the atomic level. Additionally, self‐assembled custom DNA nanostructures present significant potential in guiding mineralization processes.^[^ ^123^ ^]^ For example, double‐stranded DNA (dsDNA) has been shown to direct mineralization via the polymer‐induced liquid precursor (PILP) method, providing scaffold templates that control the growth of minerals at various length scales.^[^ ^125^, ^126^ ^]^

While Liu et al. focused on the role of DNA nanostructure primary sequence in mineralization, Patoine et al.^[^ ^55^ ^]^ highlighted the importance of DNA secondary structure, reporting a DNA aptamer (Aptamer G) that facilitates calcium phosphate mineralization within collagen fibers. Aptamer G, with its low rigidity yet highly adaptable quadruplex secondary structure, engages in a balanced interaction with both collagen and calcium phosphate precursors. This conformation change, triggered by the presence of calcium ions and collagen, slows the mineralization kinetics and promotes intra‐fibrillar mineralization of collagen without disrupting external mineral growth (Figure 9A(b)). The secondary structure of the aptamer thus mediates a balanced interaction that fosters the formation of bone‐like structures under controlled mineralization dynamics.

Furthermore, certain DNA components exhibit osteoinductive properties. For example, osteogenesis‐related plasmids, such as pBMP‐2, can participate in gene delivery to induce osteogenesis and bone formation.^[^ ^57^, ^127^ ^]^ tFNAs have also been shown to upregulate osteogenesis‐related signaling pathways, such as Wnt/β‐catenin^[^ ^111^ ^]^ and Notch,^[^ ^128^ ^]^ to promote stem cell differentiation toward an osteogenic lineage. Moreover, PDRN has been demonstrated to possess bone‐inductive properties,^[^ ^129^ ^]^ including the ability to promote osteoblast homing.^[^ ^130^ ^]^

##### DNA‐Based Hydrogel Structure for Bone Regeneration

The ideal elastic modulus for materials intended for bone regeneration typically ranges between 20 and 30 GPa,^[^ ^131^ ^]^ requiring a careful balance of stiffness and elasticity at the nanoscale,^[^ ^132^ ^]^ along with biomimetic properties that support bone tissue regeneration. Recent studies have shown that 2D DNA nanoporous scaffolds can effectively replicate the elastic modulus of native bone tissue, promoting pre‐osteoblast adhesion, proliferation, and osteogenic differentiation.^[^ ^133^ ^]^ DNA hydrogels, which feature a 3D DNA network, are expected to provide scaffolds that support the survival and growth of osteogenic cells, making them a promising candidate for bone tissue engineering applications.

Athanasiadou et al.^[^ ^64^ ^]^ introduced Y+L‐type DNA hydrogels for bone regeneration. These DNA hydrogels immersed in a mineralizing solution demonstrated the ability to fully cover hydroxyapatite (HAp) crystallites within just 16 h of mineralization, emphasizing their capacity to support mineral growth even in the absence of cells. In the 5‐mm rat calvarial critical‐size bone defects model, the osteoinductive effect of the DNA hydrogel itself was characterized by early osteogenesis, potentially driven by its relatively rapid degradation. However, the adenine released during degradation further promoted cell migration and osteogenic differentiation,^[^ ^134^, ^135^ ^]^ thereby ensuring sustained biological effects. Although the therapeutic efficacy of DNA hydrogels remains less than that of autologous bone grafts, these findings provide crucial insights for further material optimization and highlight their potential.

The integration of DNA components with DNA hydrogel further enhances the potential of these materials for bone tissue regeneration. For instance, Han et al.^[^ ^54^ ^]^ developed a DNA hydrogel loaded with tFNAs, which was further modified with Aptamer02, a peptide aptamer known to promote vascular regeneration, to accelerate the repair of critical‐sized bone defects. As previously discussed, the rapid degradation of DNA hydrogels limits their long‐term efficacy in vivo. To address this issue, Han et al. incorporated polycaprolactone (PCL) as a rigid scaffold, enhancing the structural stability of the hydrogel without compromising its porosity or the ability to support cell adhesion. Their findings revealed that Apt02‐tFNA modulated cell dynamics through multiple signaling pathways, promoting osteogenic differentiation of BMSCs. The aptamer modification of tFNAs also significantly facilitated vascular network formation of human umbilical vein endothelial cells (HUVECs), with higher expression of angiogenesis‐related genes compared to the DNA hydrogel/tFNA/scaffold (tDNA@S) group (*p* < 0.01). In vivo animal experiments using the Sprague Dawley (SD) rat cranial defect model demonstrated that the combination of Apt02‐tFNA and the DNA hydrogel resulted in the most efficient bone formation in 4 and 8 weeks post‐injury compared to the Control group (*p* < 0.01). This was evidenced by the development of a hybrid layer of newly formed bone and scaffold material, which interlock with each other to create a stable, integrated structure (Figure 9B). While direct comparative studies with autologous bone grafts are needed to fully establish clinical translatability, the demonstrated benefits highlight the potential of this hybrid DNA hydrogel system as a promising candidate for bone defect repair.

In summary, DNA‐based hydrogels exemplify a synergistic interplay between molecular design and macroscopic functionality in bone regeneration. At the molecular level, the programmable DNA nanostructures and DNA components enable precise templating of calcium phosphate mineralization and osteoinduction. Therefore, DNA hydrogels establish a dual‐axis regenerative mechanism with these structural and biological cues: mineralized scaffolds provide mechanical support akin to natural bone, while DNA‐driven molecular signals orchestrate cellular processes critical for tissue repair. This molecular‐to‐macroscopic coherence makes DNA hydrogels a paradigm‐shifting platform for structurally and biologically informed bone regeneration.

#### 4.1.3 DNA Hydrogels for Controlled Drug Delivery in Bone Regeneration

Owing to their distinctive physical properties and dynamic covalent chemistry, DNA hydrogels have emerged as a promising platform for drug delivery. In the context of bone regeneration, DNA hydrogels offer three intrinsic advantages: 1) their 3D porous structure and non‐covalent interactions with drugs enable efficient encapsulation and controlled release, minimizing adverse drug effects; 2) the combination of shear‐thinning and self‐healing properties facilitates minimally invasive drug delivery, allowing for rapid adaptation to defect morphology and volume restoration post‐injection; and 3) the structural programmability offers customization of mechanical properties and biological effects to suit diverse bone regeneration scenarios.

##### DNase‐Enabled Temporal Control

While DNA hydrogels have shown promise in achieving sustained local drug release, this approach primarily extends drug activity over time and does not allow for precise spatiotemporal control. Bone tissue regeneration involves complex and interdependent signaling pathways, necessitating more sophisticated drug delivery systems for precise temporal management.

The development of controlled‐release hydrogels typically depends on external stimuli, such as light or heat,^[^ ^136^, ^137^, ^138^ ^]^ to trigger structural changes within the hydrogel. For DNA hydrogels, natural degradation of the DNA chain by nucleases positions them as strong candidates for controlled release carriers. As natural biomolecules, nucleases exhibit high catalytic efficiency, lower systemic toxicity, and improved biocompatibility compared to other external stimuli.^[^ ^139^ ^]^ Gačanin et al.^[^ ^140^ ^]^ developed a DNase‐mediated protein‐DNA hybrid hydrogel for spatiotemporal control over the release of active proteins. In their system, recombinant C3 toxin was incorporated into the hybrid hydrogel, with DNase used to regulate its release. DNase‐controlled spatiotemporally release of C3 toxin inhibited osteoclasts over time, allowing for localized application at sites of bone resorption. This demonstrated the system's efficacy in controlling both the timing and location of drug release. Although the hybrid hydrogel requires precise assembly, the researchers modularized the preparation process, enabling stable incorporation of various DNA‐tagged cargos, including proteins, enzymes, and antibiotics, significantly broadening the potential applications of this system.

##### Optimizing DNA Hydrogel Delivery

Numerous studies have demonstrated the potential of DNA hydrogels to deliver small molecules for efficient drug encapsulation and sustained release.^[^ ^106^, ^141^ ^]^ However, DNA hydrogels also exhibit certain performance limitations. The relatively weak mechanical properties of DNA hydrogels restrict their applicability in the mechanically demanding environment of bone regeneration. Additionally, the rapid degradation rate of DNA hydrogels makes it challenging to meet the prolonged drug release duration required for long‐term bone regeneration. Therefore, novel strategies need to be introduced to address these current limitations while preserving the inherent advantages of DNA hydrogels, such as their drug‐loading capabilities.

For instance, Miao et al.^[^ ^42^ ^]^ reported a halloysite nanotube (HNT)‐reinforced DNA hydrogel delivery system, wherein dexamethasone (Dex) was incorporated to promote in vivo bone regeneration. The interactions between HNTs and the DNA backbone provided additional crosslinking sites, thereby increasing the hydrogel's network strength and mechanical stability. Importantly, this crosslinking did not impair the shear‐thinning ability or injectability of the DNA hydrogel. The double‐walled, hollow tubular structure of HNTs, with its positively charged inner surface, allowed the limited encapsulation of negatively charged Dex within the lumen, thereby facilitating drug entrapment. Compared to direct encapsulation of Dex in DNA hydrogels, the incorporation of HNTs significantly improved the sustained release profile, extending the drug's half‐life from 3 to 12 days. This modification not only enhances suitability for long‐term bone regeneration applications but also mitigates the adverse effects associated with burst drug release,^[^ ^142^ ^]^ allowing for positive regulation of MSC‐mediated osteogenic differentiation.

In contrast, Basu et al.^[^ ^52^ ^]^ focused on hybrid DNA hydrogels to develop a DNA hydrogel network crosslinked by oxidized alginate. The imine bonds were formed between the aldehyde groups of oxidized alginates and the amine groups in DNA's nucleobases. This reversible bonding preserved the shear‐thinning and self‐healing properties of the DNA hydrogel. By adjusting the oxidation degree of OA to interfere with covalent crosslinking, the hydrogel's performance could be fine‐tuned. For example, increasing the oxidative degree of OA (oleic acid) from 21.64% to 53.76% can lead to a nearly 15‐fold increase in yield stress (from 12.59 Pa to 199.5 Pa). The covalent crosslinking also significantly enhanced the mechanical performance of the hybrid hydrogel, improving storage modulus and recovery after high‐strain deformation. The authors observed that the half‐life of the small‐molecule drug simvastatin within the hybrid DNA hydrogel was extended to 5 days without burst release. This suggests that drug release is not diffusion‐controlled,^[^ ^143^ ^]^ emphasizing the critical role of DNA‐mediated weak molecular interactions in governing drug release kinetics.

In summary, through strategic engineering at both molecular and macroscopic scales, these hydrogels overcome intrinsic limitations such as mechanical fragility and rapid degradation while preserving their inherent advantages including precise drug encapsulation, injectability, and biocompatibility. The adaptability of DNA‐based networks allows for tunable mechanical properties and sustained release behavior, ensuring alignment with the biomechanical and biochemical demands of bone repair.

#### 4.1.4 Context‐Driven Design Strategies of DNA‐based Hydrogels in Bone Regeneration

The design strategies of DNA‐based hydrogels for bone regeneration exhibit a clear dependency on the specific context. In normal physiological conditions, the design primarily focuses on the spatiotemporal coordination of angiogenesis and osteogenesis. However, in pathological conditions such as diabetes, the design must integrate multidimensional functions, including inflammation suppression, immune modulation, and infection defense. This differentiation in approach arises from the unique dynamic programmability of DNA molecules—where the reversibility of base complementary pairing endows the hydrogel with environmental responsiveness.

##### Synergistic Interaction between Bone Regeneration and Vascularization

Bone vascularization plays a critical role in bone regeneration. Vascular networks not only ensure the metabolic balance of bone tissue by delivering essential nutrients and clearing metabolic waste but also maintain the local microenvironment's homeostasis.^[^ ^144^ ^]^ Furthermore, the vascular system facilitates essential biological signaling interactions between bone and surrounding tissues, thereby promoting the health of both.^[^ ^145^ ^]^ Consequently, implant materials must incorporate both angiogenic and osteogenic capabilities to establish an effective vascular network at the site of regeneration, preventing local bone necrosis.

**Figure 10:**
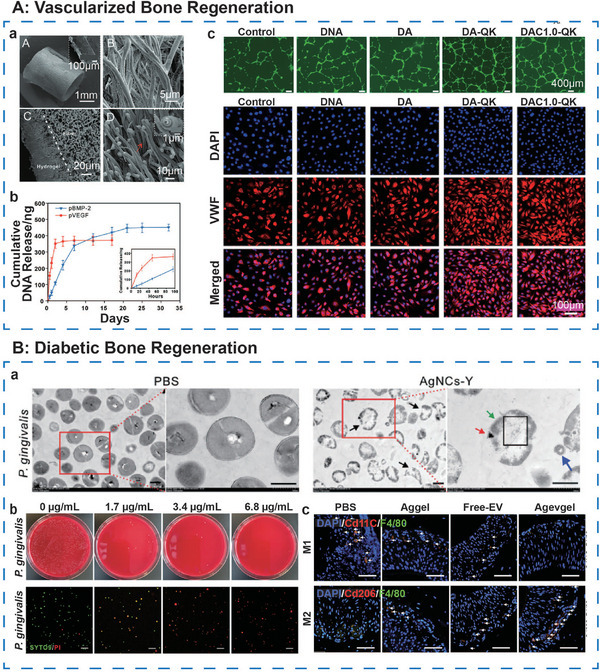
Context‐Driven Design Strategies of DNA‐based Hydrogels in Bone Regeneration. **A**: **a** SEM images of the core–sheath structured fiber‐hydrogel scaffold. **b** The release kinetics of two types of pDNA incorporated in the core–sheath structured composite scaffold. Reproduced with permission.^[^ ^110^ ^]^ Copyright 2022, Wiley. **c** Calcein‐AM staining and immunofluorescence staining images of HUVECs. Reproduced with permission.^[^ ^53^ ^]^ Copyright 2023, ACS. **B**: **a** TEM images of *P. gingivalis* after treating with PBS or AgNCs‐Y (black arrow‐broken cell wall, green arrow‐obscure cell wall, the red arrow ‐the condensed components, blue arrow‐detached cell wall. Scale bar=500 nm). **b** Colony‐forming assay and live/dead images of *P. gingivalis* after treating with AgNCs‐Y in different concentrations. Reproduced with permission.^[^ ^45^ ^]^ Copyright 2024, Wiley. **c** Immunofluorescence staining images showing Agevgel regulated macrophage polarization. Reproduced with permission.^[^ ^141^ ^]^ Copyright 2023, Wiley.

A key strategy in addressing this challenge is the development of dual bioactive molecule delivery systems that facilitate the coordinated promotion of bone regeneration and vascularization, such as dual gene delivery systems. He et al.^[^ ^110^ ^]^ introduced a gene‐activated matrix featuring a core–sheath structure to enable the temporal release of plasmid DNA, effectively mimicking the biological signaling cues associated with natural bone vascularization. In this system, the hydrogel core houses pVEGF, which is released in an initial burst over the course of one week, transitioning into a plateau phase to induce neovascularization. Meanwhile, pBMP2, carried on a PDA‐coated fibrous sheath, is released in a near‐zero‐order fashion for the first 96 h, followed by a sustained release over 21 days to induce long‐term osteogenic signaling (**Figure** **10**A(a,b)). This design replicates the spatiotemporal control of gene release observed in natural bone vascularization, facilitating effective bone tissue regeneration in the critical‐sized segmental femoral defect model in rats.

However, the successful integration of bone regeneration and vascularization requires the coordination of a dynamic microenvironment. Under normal physiological conditions, the native bone ECM provides a dynamic environment essential for bone regeneration. This ECM offers mechanical strength while mediating the delivery of growth factors, mechanical signaling, and cell–cell communication, thus guiding cellular behaviors.^[^ ^79^, ^146^ ^]^ Accordingly, the development of DNA hydrogels that mimic the properties of the native bone ECM offers a promising avenue for achieving synergistic effects in bone vascularization.

In this context, a well‐established design framework has emerged: 1) DNA networks impart dynamic characteristics that enable mechanical signal transmission while mimicking the viscoelasticity of native ECM; 2) Hybrid dual‐network structures introduce tunable mechanical strength to provide essential support during the repair process; 3) The excellent controlled release capabilities of DNA hydrogels facilitate the coordinated delivery of osteogenic and angiogenic signaling factors.

For instance, Yang et al.^[^ ^53^ ^]^ reported a dual‐nanostructured dynamic DNA hydrogel in which the reversible properties of the DNA network effectively mimic the dynamic behavior of native ECM. In addition, supramolecular co‐assembly of amyloid fibrils (AF) with the DNA scaffold enhanced the mechanical strength and stability of the hydrogel, while introducing a fibrous matrix to emulate the ECM's structural features. Moreover, the chemical grafting of the QK peptide (a VEGF mimetic peptide) onto AF stimulated vascular formation and migration of HUVECs (Figure 10A(c)), while the inclusion of clay nanosheets enabled the sustained release of Si^4+^ and Mg^2+^, promoting osteogenic differentiation of BMSCs significantly (the DAC Hydrogel group significantly higher than the control group and other hydrogel groups, *p* < 0.001). This approach effectively achieves the synergistic biological effects of bone regeneration and vascularization.

As DNA hydrogel‐based design strategies continue to evolve, future research holds the potential to refine microenvironmental and functional component control, enabling more effective bone vascularization and regeneration and advancing clinical applications in regenerative medicine.

##### Inflammation and Immune Modulation in Pathological Microenvironments

The hyperglycemic microenvironment associated with diabetic bone regeneration poses multiple pathological challenges, including osteogenesis inhibition driven by chronic inflammation,^[^ ^147^ ^]^ immune dysregulation‐induced vascular degeneration,^[^ ^148^ ^]^ and repair process disruption resulting from heightened infection susceptibility.^[^ ^149^, ^150^ ^]^ By virtue of their dynamic responsiveness and multifunctional integration capabilities, DNA‐based hydrogels are evolving towards a progressive strategy characterized by “pathological signal sensing—immune homeostasis restoration—antibacterial defense synergy”, providing novel solutions to overcome the barriers to bone regeneration in diabetes.

To address the inflammatory microenvironment of diabetes, Jing et al.^[^ ^47^ ^]^ introduced a dynamic delivery system responsive to pathological signals. Their PEG/DNA hybrid hydrogel contains enzyme‐cleavable sites responding to high matrix metalloproteinase‐9 (MMP‐9) concentrations, which dissociate to enable the controlled release of exosomes derived from dental papilla stem cells (SCAP‐Exo). This system leverages a miRNA dual‐axis mechanism to simultaneously inhibit inflammation and activate osteogenic pathways, resulting in a 40% improvement in bone regeneration efficiency in a diabetic rat model. This design paradigm overcomes the limitations of passive release in conventional hydrogels, allowing for the dynamic alignment of therapeutic factor release with disease progression.

DNA hydrogels offer an ideal platform for the delivery of cytokines with immune modulation ability. Li et al.^[^ ^151^ ^]^ developed an IL‐10‐loaded DNA hydrogel (ILGel), a hydrogel that physically crosslinks DNA networks to encapsulate IL‐10. The hydrogel's porous structure sustains the bioactivity of IL‐10 for over seven days. This system induces M2 macrophage polarization, decreasing the expression of pro‐inflammatory cytokines while upregulating osteogenic markers, resulting in a 93.4% healing rate of diabetic alveolar bone (*n* = 5, *p* < 0.001). Notably, the study reveals a positive feedback loop involving M2 macrophages and endogenous IL‐10 secretion, which amplifies anti‐inflammatory effects during later stages of tissue repair. This self‐amplifying mechanism of immune modulation constructs a self‐stabilizing microenvironment.

With an expanding understanding of pathological complexity, the integration of antibacterial and immune‐regulatory functions has become a key trend. Peng et al.^[^ ^45^ ^]^ innovatively co‐loaded DNA‐templated AgNCs and TNF‐α antibodies within DNA hydrogels to create a synergistic system with multiple functions. AgNCs reduce the survival of *P. gingivalis* through bacterial membrane disruption (Figure 10B(a,b)), while the sustained release of TNF‐α antibodies (for >10 days) reduced macrophage infiltration by 2.5 times (*n* = 3, *p* < 0.0001) compared to the control group, leading to a high bone defect healing rate. This “pathogen clearance—immune remodeling” dual strategy effectively addresses the clinical challenge of infection and inflammation.

Recent advancements have taken functional integration to a higher dimension. Peng's team developed Agevgel, a hydrogel that co‐delivers AgNCs and M2 macrophage exosomes (M2EVs) within a DNA network to construct a four‐dimensional functional system: 1) AgNCs provide broad‐spectrum antibacterial activity; 2) M2EVs promote osteogenic differentiation of BMSCs through IL‐10 signaling; 3) The DNA scaffold responds to microenvironmental matrix metalloproteinases (MMPs) for controlled sequential release; and 4) The porous structure mimics the ECM to enhance cell migration^[^ ^141^ ^]^ (Figure 10B‐c). This design achieved high bone regeneration efficiency and regulated inflammation pathways, such as NPR3/IL‐6, to establish an immune homeostasis microenvironment.

By dynamically sensing local inflammatory signals, precisely modulating immune cell polarization, and integrating antibacterial‐osteogenic dual‐functional modules, these intelligent repair systems are increasingly capable of adapting to the complex pathological features of diabetes.

In summary, DNA hydrogels establish spatiotemporal coordination of angiogenesis and osteogenesis through tunable mechanical properties and controlled release of dual bioactive signals. In diabetic or inflammatory microenvironments, DNA hydrogels evolve into multifunctional platforms that restore immune homeostasis and combat infection simultaneously. These hydrogels achieve hierarchical regulation of bone repair processes by incorporating environmental sensing and precise biological targeting. This context‐specific design strategy reveals DNA hydrogels’ capacity of therapeutic precision and microenvironmental adaptation, enabling robust bone regeneration across diverse clinical scenarios.

Above all, among the promising candidates in bone regeneration, DNA‐based hydrogels have emerged as a focal point due to their unique abilities and design methods:

1. Mineralization guidance and osteoinductive properties via programmable DNA nanostructures that template calcium phosphate crystallization and activate osteogenic signaling pathways.
2. Biomimetic mechanical adaptability through tunable stiffness, viscoelasticity, and shear‐thinning injectability.
3. Drug delivery with spatiotemporal control and sustained release, enabled by dynamic DNA networks and enzyme‐responsive degradation.
4. Precise sensing of pathological signals and multifunctional microenvironment regulation, by integrating agents with immune‐modulating and antibacterial activity.

### 4.2 Cartilage Regeneration

Due to the avascular and aneural nature of cartilage tissue, its regenerative potential is limited.^[^ ^152^ ^]^ Existing clinical interventions, including palliative pharmacological treatments and surgical regenerative approaches, are constrained by limited efficacy and unpredictable long‐term outcomes.^[^ ^153^ ^]^ In this context, cartilage tissue engineering has emerged as a promising strategy to overcome the shortcomings of conventional therapies. Among the various biomaterials under investigation, DNA‐based hydrogels offer unique viscoelastic properties that mimic the natural matrix, providing an ideal 3D microenvironment for cartilage regeneration. Furthermore, these materials hold promise as potential therapeutic targets. In this section, we provide a comprehensive overview of the different fabrication strategies for DNA‐based hydrogels in cartilage repair, highlighting their potential to advance the field of cartilage regeneration.

#### 4.2.1 Biology of Cartilage Tissue Regeneration

Cartilage is a specialized form of connective tissue known for its exceptional elasticity and resilience, playing a pivotal role in the structural integrity and maintenance of numerous essential tissues and organs.^[^ ^154^ ^]^ It provides both mechanical support and shock absorption across a variety of anatomical systems. Notably, articular cartilage, which covers the ends of bones in synovial joints, is critical for load distribution and lubrication during joint movement. Articular cartilage is an avascular, aneural tissue with a zonal structure: the superficial zone (10–20% thickness) contains flattened chondrocytes and aligned collagen II fibers for shear resistance, while the deep zone features vertically oriented collagen and proteoglycans for compressive load distribution.^[^ ^155^ ^]^ The compressive modulus of articular cartilage ranges from 0.1 to 1.5 MPa due to its biphasic viscoelastic properties,^[^ ^156^ ^]^ which derive from fluid pressurization within the proteoglycan‐rich matrix.^[^ ^157^ ^]^

Articular cartilage regeneration is inherently limited by its avascular structure and low chondrocyte turnover. However, various factors—including trauma,^[^ ^158^ ^]^ aging,^[^ ^159^ ^]^ and autoimmune disorders^[^ ^160^ ^]^—can disrupt the homeostatic balance, leading to accelerated degradation of the ECM and a diminished capacity for chondrocyte synthesis. Injury or degeneration disrupts the superficial zone's lubricating proteoglycans, increasing shear stress and activating a Disintegrin and Metalloproteinase with Thrombospondin motifs (ADAMTS‐5)/MMP‐13 to degrade aggrecan and collagen II.^[^ ^161^ ^]^ The resulting fibrocartilaginous repair tissue, rich in type I collagen, exhibits quite low compressive modulus, predisposing joints to mechanical failure. In pathological conditions like osteoarthritis, mitochondrial ROS overproduction triggers chondrocyte apoptosis through endoplasmic reticulum (ER) stress and caspase‐3 activation, exacerbated by multiple signaling pathways.^[^ ^162^, ^163^ ^]^ Therefore, advanced therapeutic approaches are urged to enhance chondrogenic potential.

#### 4.2.2 Stem Cell Therapy in Cartilage Regeneration

In recent years, tissue engineering and regenerative strategies utilizing MSCs have shown substantial promise in addressing cartilage repair.^[^ ^164^ ^]^ However, cartilage repairs are often required in load‐bearing joints, such as the knee and hip, where the presence of inflammation creates a challenging environment that impairs effective regeneration.^[^ ^165^ ^]^ Despite MSCs' robust differentiation potential and therapeutic secretory capabilities, their efficacy in cartilage regeneration still depends on the performance of delivery systems and scaffolds, which provide protection and promote adhesion and differentiation. To this end, a variety of DNA‐based hydrogel structures have been engineered as MSC delivery scaffolds, incorporating diverse strategies aimed at enhancing the repair efficacy of MSCs through these “MSC‐scaffold” systems.

##### DNA Nanostructure‐Facilitated MSCs Interacting

**Figure 11:**
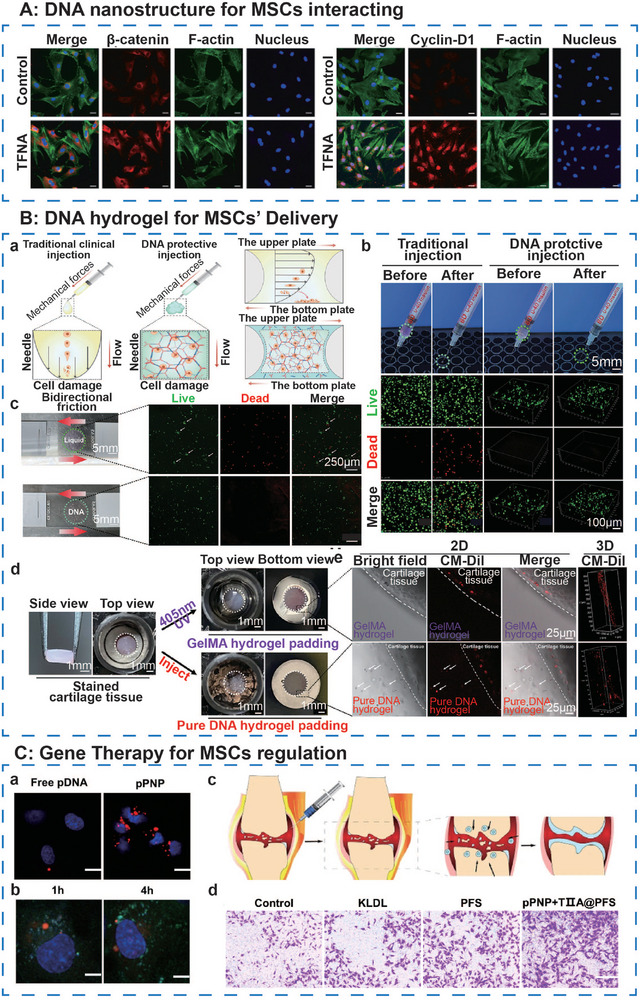
DNA‐based hydrogel‐facilitated stem cell therapy for cartilage repair. A) Immunofluorescence images of Wnt pathway‐related proteins in tFNAs treated SMSCs. Adapted under the terms of the CC BY‐NC‐ND license 4.0 from Ref. [170] Copyright 2021, Elsevier. B, a) Schematic images of cell protection ability in the liquid carrier and DNA supramolecular hydrogel during injection and bidirectional friction. b,c) Viability of BMSCs after b) injection and c) bidirectional friction. d,e) Macroscopic and fluorescence images of fresh rabbit knee cartilage discs cultured in GelMA hydrogel or DNA supramolecular hydrogel for 48 h. Reproduced with permission.^[^ ^76^ ^]^ Copyright 2021, Wiley. C, a) Cellular uptake of free pDNA or pPNP by chondrocytes. b) Images of cellular uptake behavior of pPNPs for 1 and 4 h. c) Schematic image of the recruitment mechanism of BMSCs. d) Transwell images of chondrocytes after culture in different treatment groups for 24h (Scale bar = 500 µm). Adapted under the terms of the CC BY‐NC‐ND license 4.0 from Ref. [180] Copyright 2024, BMC.

Tetrahedral Framework Nucleic Acids (tFNAs) have shown exceptional biocompatibility and the ability to modulate cellular behaviors, particularly in promoting MSC proliferation and differentiation,^[^ ^166^, ^167^ ^]^ while simultaneously alleviating inflammation and oxidative stress in osteoarthritis.^[^ ^168^ ^]^ Therefore, intra‐articular injection of tFNAs presents a promising alternative to regenerative stimuli (e.g., TGF‐β1 and BMP‐2) carried by scaffolds, improving the regenerative microenvironment without inducing adverse pathological changes.^[^ ^169^ ^]^ However, challenges remain in achieving precise MSC targeting, and drug delivery efficiency remains suboptimal. To address these limitations, Li et al.^[^ ^170^ ^]^ developed a chitosan (CS) hydrogel/3D‐printed poly(ε‐caprolactone) (PCL) hybrid scaffold containing synovial MSCs (SMSCs). The scaffold utilizes the electrostatic interaction between the negatively charged tFNAs and positively charged CS to recruit tFNAs effectively. Experimental results showed that tFNAs at a concentration of 250 nm promoted the transition of the cell cycle from the G1 to S phase, and upregulated the Wnt/β‐catenin pathway to enhance SMSC proliferation (**Figure** **11**A). Additionally, tFNAs induced chondrogenic differentiation, improving both the quality and mechanical properties of regenerated cartilage, while also modulating the intra‐articular environment. However, the relatively short degradation period of tFNAs limits their long‐term interaction with SMSCs in vivo, necessitating repeated injections to maintain therapeutic efficacy. Given that the cartilage matrix contains a high concentration of negatively charged proteoglycans,^[^ ^171^ ^]^ tFNAs are repelled by the cartilage matrix after intra‐articular injection but are directionally recruited by the CS hydrogel, ensuring their effective use. However, repeated injections may increase the risk of joint infections.^[^ ^172^ ^]^ Therefore, future research should focus on extending the degradation stability of tFNAs, as well as investigating the balance between their degradation and the regeneration of new tissue in the joint microenvironment to optimize therapeutic outcomes.

In another study, Chen et al.^[^ ^173^ ^]^ utilized in situ electrospinning technology to develop rosette nanotube (RNT)/hydrogel composites embedded with cells, demonstrating their promising application in cartilage injury. RNTs are formed through a hierarchical self‐assembly process, where the basic building block is a G∧C heteroaromatic bicyclic base, mimicking the Watson–Crick hydrogen bonding patterns of guanine and cytosine. These G∧C bases self‐assemble into six‐membered supermacrocycles shaped like rosettes, which then stack into nanotubular structures.^[^ ^174^ ^]^ Moreover, electrospinning effectively simulates the nanostructured environment of natural extracellular matrices, enabling the direct deposition of hydrogel fibers containing live cells onto the target tissue site.^[^ ^175^ ^]^ The results indicated that the nanoscale roughness of the RNTs on submicron hydrogel fibers closely resembled that of natural cartilage tissue, thereby enhancing the viability and adhesion of synovial fibroblasts (SFBs) and chondrocytes, and promoting chondrogenic differentiation of SFBs. Additionally, the incorporation of RNTs improved the adhesion strength of the hydrogel, simulating cleaved collagen and reinforcing the composite's physical properties as a tissue sealant.

##### Dynamic DNA Hydrogel for MSCs Delivery Protection

DNA chains are capable of forming supramolecular networks through base‐pairing interactions, which can be further utilized to create hydrogel scaffold systems. For instance, Yan et al.^[^ ^76^ ^]^ developed a DNA supramolecular hydrogel as a carrier for BMSCs in the treatment of OA (Figure 11B). This DSH is composed of two DNA building blocks: Y‐shaped scaffolds and linear linkers, which spontaneously self‐assemble into a gel, avoiding additional induction processes that could harm the cells.^[^ ^84^ ^]^ Experimental results showed that BMSCs encapsulated in DSH could be successfully extruded from a 29G syringe needle (with an inner diameter much smaller than that of previously reported injectable hydrogel systems), minimizing patient discomfort. The shear‐thinning behavior of DSH allows it to transition from a gel to a liquid under high shear strain, mitigating the shear forces encountered during injection and allowing the material to return to its gel state post‐injection. This property minimizes cellular damage during injection, with over 99% of encapsulated cells surviving and remaining evenly distributed; however, the statistical significance was not achieved (*n* = 3, *p* > 0.05), requiring further verification.​​ In a high‐friction model simulating severe OA, DSH formed a lubricating layer inside the plate, which eliminated lateral shear forces within the joint cavity. Therefore, DSH can effectively prevent both short‐term shear forces during injection and long‐term joint friction, significantly improving the efficacy of OA treatment. RNA sequencing further revealed that BMSCs encapsulated in DSH exhibited stronger anti‐inflammatory and chondrogenic effects compared to those encapsulated in traditional liquid carriers, demonstrating the potential for further clinical translation.

##### Gene Therapy for MSCs Regulation

Gene therapy facilitates the direct expression of nucleotides in target tissue cells through intracellular delivery. In cartilage, the limited blood flow helps maintain a localized concentration of gene therapeutics, reducing the risk of degradation and prolonging the interaction time with target cells. Although this may extend the delivery time of DNA polymers, using smaller plasmid DNA with lower molecular weight can mitigate this challenge and enhance delivery efficiency. Compared to traditional adenovirus vectors, hydrogels offer superior biosafety by avoiding the strong immune responses and side effects commonly associated with viral vectors.^[^ ^176^ ^]^ Furthermore, by fine‐tuning the physical properties of the hydrogel system, plasmid DNA can be released in a controlled, targeted manner, enabling precise regulation of degradation rates. These gene delivery advantages of hydrogels hold particular promise for overcoming critical limitations in MSC‐based cartilage regeneration. By integrating gene‐activated hydrogels with MSC delivery systems, researchers can engineer microenvironments that actively steer stem cell fate, thereby bridging the gap between localized gene therapy and cellular reprogramming strategies.

The introduction of exogenous mesenchymal stem cells (MSCs) to induce cartilage differentiation is a key strategy in cartilage tissue engineering. However, MSCs used in joint cartilage regeneration often exhibit tendencies toward hypertrophy and endochondral ossification, which can hinder effective cartilage repair.^[^ ^177^ ^]^ To overcome this challenge, Gonzalez‐Fernandez et al.^[^ ^178^ ^]^ developed a hydrogel containing plasmid DNA encoding therapeutic factors transforming growth factor‐β3 (TGF‐β3) and BMP‐2, allowing for precise control over MSC phenotype. In this system, calcium ions from hydroxyapatite nanoparticles (nHA) interact with the negatively charged phosphate groups of plasmid DNA, forming nHA‐pDNA complexes that are incorporated into an alginate hydrogel containing MSCs. The study demonstrated that nHA nanoparticles promote sustained plasmid DNA internalization, prevent peripheral degradation, and facilitate effective gene transfection for at least 14 days in culture. When delivered separately, TGF‐β3 or BMP‐2 genes increased type X collagen production, indicating hypertrophy and endochondral ossification of chondrocytes. In contrast, co‐delivery of pTGF‐β3/BMP‐2 suppressed calcification and type X collagen deposition, while enhancing the production of GAGs and type II collagen, thereby promoting a more stable chondrogenic phenotype. Although the precise molecular mechanisms remain to be fully elucidated, this approach can effectively guide MSC phenotypic fate during cartilage regeneration.

Although MSCs are a promising source of chondrogenic cells, their therapeutic efficacy in cartilage repair remains uncertain.^[^ ^179^ ^]^ Endogenous joint‐resident MSCs offer an alternative cell source, with the potential for cartilage repair through stem cell homing to the site of injury. Wang et al.^[^ ^180^ ^]^ developed a hydrogel incorporating a stem cell homing peptide to recruit endogenous BMSCs and promote cartilage regeneration (Figure 11C). The hydrogel contained plasmid DNA (pKlotho)‐loaded peptide nanoparticles (pPNPs) targeting the Klotho gene (KL), and introduced a cartilage‐targeting sequence and nuclear localization signal (NLS) for precise gene delivery. Klotho, an anti‐aging molecule, maintains chondrocyte homeostasis by downregulating the expression of catabolic markers such as ZIP8 and MMP‐13.^[^ ^181^ ^]^ Experimental results showed that the pPNP + TIIA@PFS hydrogel system exhibited excellent lubricating properties, protecting the joints. Furthermore, the stable electrostatic interactions between pPNPs and the hydrogel allowed for sustained release of the therapeutic agents (up to 40 days), with no interference from joint movement. In vivo studies demonstrated that this approach reduced lesion incidence, restored cartilage integrity, and resulted in knee joints from an ACLT rat model of OA that exhibited physiological characteristics similar to those of healthy joints, suggesting the potential rejuvenation of aging joints.

Looking ahead, further development of target pathways and comprehensive long‐term safety assessments could enable successful clinical translation of gene therapy in cartilage repair.

The high tunability of DNA chains allows for the precise adjustment of both mechanical and biological properties, making DNA‐based hydrogels highly versatile for a wide range of MSC delivery applications. The studies discussed herein offer novel insights and innovative strategies for the development of advanced MSC delivery systems in cartilage repair.

#### 4.2.3 Drug Delivery in Cartilage Regeneration

The application of cartilage tissue engineering extends beyond simple traumatic injuries to include a range of pathological conditions, such as inflammatory cartilage damage, synovitis, and subchondral bone sclerosis, all of which are central to diseases such as osteoarthritis and rheumatoid arthritis.^[^ ^182^ ^]^ Therefore, cartilage repair in these complex diseases requires drug delivery platforms to target the underlying causes and also employ tissue regeneration methods.

DNA supramolecular hydrogels represent a promising class of materials with significant potential in drug delivery, particularly for controlled release applications. These materials can reversibly form or break chemical bonds in response to specific stimuli, leading to the generation of new structures. Such a unique property is especially beneficial for controlled drug release.^[^ ^183^ ^]^ Additionally, the high programmability of DNA strands enables the modulation of pore sizes by altering the number of bases in ssDNA, making DSH versatile for the delivery of drugs with varying molecular weights.^[^ ^184^ ^]^ Furthermore, the intrinsic anti‐inflammatory and antioxidant properties of DNA strands can also help alleviate local inflammation, making DSH highly suitable for creating customized drug delivery platforms for inflammatory diseases.^[^ ^185^ ^]^ Building on these characteristics, Zhang et al.^[^ ^62^ ^]^ reported a DSH‐based metformin delivery platform for the treatment of OA induced by anterior cruciate ligament transection (ACLT). Experimental results demonstrated that the synovial macrophages in the knee joint of mice in an ACLT model transitioned to an anti‐inflammatory phenotype, suppressing the secretion of inflammatory cytokines, while chondrocytes exhibited reduced mitochondrial membrane potential and inhibited apoptotic behavior, thus promoting local inflammation resolution and the restoration of cartilage health.

Moreover, the use of nucleic acid aptamers allows for precise drug delivery to specific therapeutic targets, thereby minimizing off‐target effects and enhancing delivery efficiency.^[^ ^68^, ^69^ ^]^

In conclusion, the further structural refinement of DSH and the development of aptamers targeting additional therapeutic sites, either independently or in combination, present promising strategies for advancing cartilage repair and the treatment of inflammatory joint diseases.

Above all, DNA‐based hydrogels represent a transformative platform for cartilage regeneration through their multifunctional integration of biomimetic design and therapeutic precision:

1. Biomimetic matrix recapitulation that mimics the structural and mechanical properties of native cartilage, providing an optimal microenvironment for tissue repair.
2. Delivery protection and spatiotemporal control for stem cells and therapeutic agents, with shear‐thinning injectability and dynamic DNA networks to minimize cellular damage and enhance retention.
3. Stem cell fate regulation via gene delivery and programmable DNA components to suppress hypertrophy and stabilize chondrogenic differentiation.
4. Multifunctional microenvironment modulation that integrates mechanical lubrication, anti‐inflammatory signaling, and ECM‐mimetic nanotopography.

These innovations position DNA‐based hydrogels as a versatile strategy to overcome the dual challenges of mechanical failure and biological dysfunction in cartilage repair.

### 4.3 Skeletal Muscle Regeneration

While DNA‐based hydrogels have garnered significant attention in tissue engineering, their application in skeletal muscle regeneration remains underexplored and faces critical technical and biological barriers. This section aims to dissect the intrinsic challenges hindering the adaptation of DNA hydrogels to muscle regeneration. By analyzing these limitations and emerging preclinical breakthroughs, we further propose actionable directions to harness the untapped potential of DNA‐based systems in overcoming the persistent hurdles of functional muscle recovery.

#### 4.3.1 Biology of Skeletal Muscle Regeneration

Skeletal muscle fibers are multinucleated syncytia surrounded by a basal lamina containing laminin and collagen IV, which exhibits anisotropic tensile strength through highly organized sarcomere arrays.

The skeletal muscle repair process includes three overlapping phases: destruction, repair, and remodeling.^[^ ^186^ ^]^ Skeletal muscle regeneration relies on satellite cells (SCs) activation and precise immune coordination. The destruction phase (0–5 days) involves myofiber rupture, hematoma formation, and neutrophil/macrophage infiltration. Macrophages phagocytose necrotic debris while preserving basal lamina scaffolds for regeneration and secreting survival factors for satellite cells. The repair phase (5–14 days) features dual processes. On the one hand, satellite cells are activated to proliferation into myoblasts, fusion into myotubes, and integration with surviving myofibers; On the other hand, fibrotic scar is formed via fibroblast‐derived ECM (fibronectin, tenascin‐C, collagen I/III). The balanced progression of these two processes is crucial in determining whether the muscle contraction function can be optimally restored. The remodeling phase (14+ days) enhances functional recovery through myofiber maturation and scar condensation via collagen crosslinking. However, unlike the tissue‐matched healing property of bone regeneration, muscle repair inherently forms scar tissue with altered biomechanics. The collagen‐rich scars regain only 60–80% of original tensile strength, risking re‐injury at neo‐myotendinous junctions.^[^ ^187^ ^]^

#### 4.3.2 DNA‐Based Hydrogels in Skeletal Muscle Regeneration

From a physiological perspective, skeletal muscle is a highly mechanical tissue requiring scaffolds to not only support cellular growth but also withstand dynamic mechanical loads. Specifically, native muscle tissues endure cyclic tensile strains up to 20% during contraction‐relaxation cycles^[^ ^188^ ^]^ and exhibit hierarchical anisotropy with aligned myofiber bundles (10–100 µm diameter) along stress transmission axes.^[^ ^189^ ^]^ These dual demands pose unique challenges for DNA‐based hydrogel design: 1) Conventional DNA hydrogels typically exhibit storage moduli in the 0.1‐5 kPa range, insufficient to resist repetitive mechanical stresses without permanent deformation; 2) Achieving structural anisotropy comparable to muscle's extracellular matrix (ECM) requires precise spatial control over DNA crosslinking networks, which remains technically demanding compared to isotropic gelation approaches.^[^ ^190^ ^]^ Consequently, polymer hydrogel loaded with DNA components has emerged as a promising approach in skeletal muscle regeneration, incorporating materials with sufficient mechanical strength as a hydrogel matrix while introducing functional DNA fragments to exert therapeutic effects.

**Figure 12:**
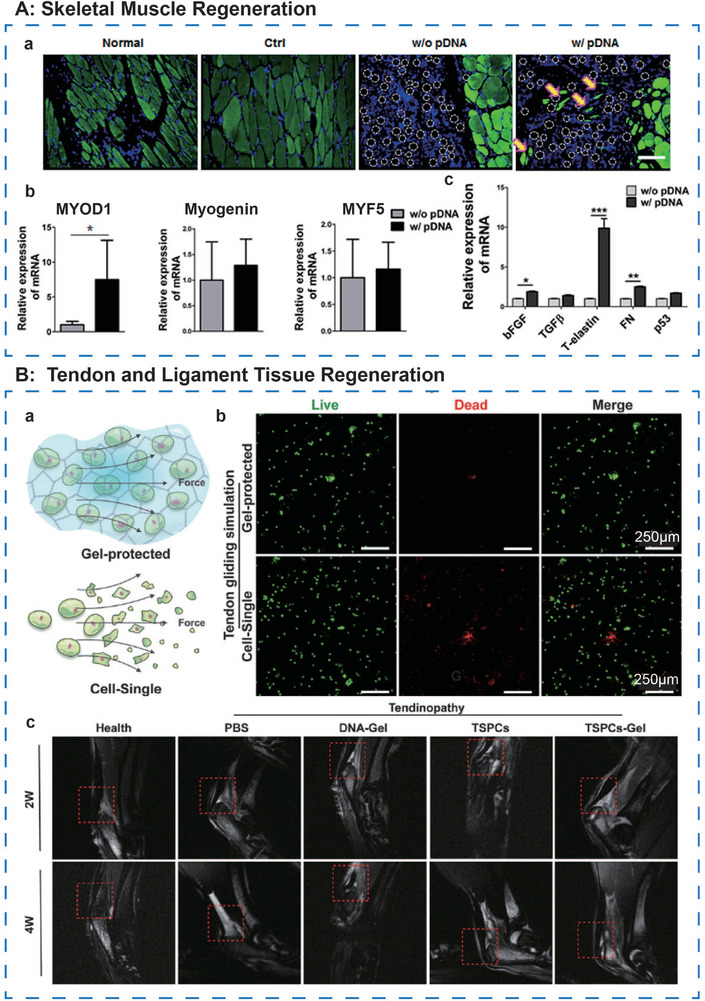
DNA‐based hydrogels in skeletal muscle, tendon, and ligament tissue regeneration. A, a) Fluorescence images of muscle regeneration in VF after injection laryngoplasty (arrow‐regenerated muscle; scale bar=500 µm). b,c) RT‐PCR results of b) muscle transcription factors and c) VF constituents from VF tissue at 4 weeks. Reproduced with permission.^[^ ^191^ ^]^ Copyright 2019, ACS. B, a) Schematic images of DNA hydrogel's cell protective ability. b) Dead/live assay of TSPCs after tendon gliding simulation. c) Images of rat Achilles tendons after 2 and 4 weeks of injection (red dashed box‐swelling). Adapted under the terms of the CC BY‐NC‐ND license 4.0 from Ref. [201] Copyright 2023, Wiley.

For instance, Kim et al.^[^ ^191^ ^]^ developed a plasmid DNA complex encoding basic fibroblast growth factor (bFGF), embedded within a hydrogel matrix dispersed with polycaprolactone (PCL) microspheres, aimed at regenerating atrophied vocal fold (VF) muscle and its lamina propria. This hydrogel encapsulated hyaluronic acid within an alginate (ALG) network, enabling stable anchoring of the pDNA complex within the gel matrix, and ensuring sustained release of the pDNA. PCL microspheres‐maintained stability at the application site and, through physical expansion over 24 weeks, prevented volumetric loss of the paralyzed VF and muscle of rabbits sectioning the recurrent laryngeal nerve (RLN) (**Figure** **12**A). The Myod1 level associated with muscle regeneration was significantly elevated in the experimental group (*p* < 0.05); however, other muscle transcription factors did not show statistically significant increases. This gel matrix provided an ideal transfection environment for pDNA, facilitating ECM remodeling, collagen and HA synthesis, and some degree of intrinsic muscle regeneration in the VF, thus offering a promising injectable material for VF paralysis rehabilitation.

In another study, Cohen et al.^[^ ^58^ ^]^ utilized a polyethylene glycol (PEG)‐fibrinogen hydrogel to control the gene delivery of antisense oligonucleotides (AONs) for exon skipping therapy in Duchenne muscular dystrophy (DMD). AONs were chemically modified to enhance stability and tissue uptake (2OMePs), and their sustained release and high transfection efficiency were achieved through encapsulation within PF microspheres. This approach significantly increased the exon skipping rate in the DMD mouse model and promoted the recovery of dystrophin expression in muscle tissue. Notably, the study demonstrated that intra‐arterial injections were more effective than traditional intra‐muscular injections, reducing the need for repeated injections and broadening the therapeutic potential for whole‐limb correction in patients with muscular dystrophy.

To summarize, integrating DNA‐based hydrogels into skeletal muscle regeneration presents an underexplored frontier in regenerative medicine. Current DNA hydrogel systems still struggle to reconcile the dual demands of dynamic load‐bearing capacity and hierarchical anisotropy required for functional myofiber alignment. Moreover, the biological complexity of scar‐modulated regeneration calls for smarter material systems capable of stage‐specific therapeutic modulation.^[^ ^192^ ^]^ Despite these challenges, DNA‐based hydrogels hold transformative potential for skeletal muscle regeneration through their programmable integration of gene‐editing tools and patient‐specific cellular therapies to address both functional recovery and scar modulation.

### 4.4 Tendon and Ligament Regeneration

As load‐bearing connective tissues with limited intrinsic regenerative capacity, tendons and ligaments present unique challenges in achieving functional repair. This section critically examines the current limitations of DNA hydrogel systems in replicating the mechanical demands and vascular complexities of these tissues, while proposing targeted strategies to unlock their role in advancing regenerative therapies for load‐bearing connective tissues.

#### 4.4.1 Biology of Tendon and Ligament Tissue Regeneration

Tendons and ligaments are fibrous connective tissues that link muscle‐to‐bone and bone‐to‐bone, respectively.^[^ ^193^ ^]^ Tendons primarily transmit muscular contraction forces to bones, while ligaments passively stabilize joints. Structurally, tendons consist of type I collagen (90% dry mass), elastin, and proteoglycans embedded within a highly hydrated matrix.^[^ ^194^ ^]^ Ligaments share similar compositional and organizational features but exhibit ECM heterogeneity depending on mechanical demands—for instance, the patellar tendon blurs the boundary between the tendon and ligament.

Tendon healing progresses through overlapping inflammatory, proliferative, and remodeling phases.^[^ ^195^ ^]^ During the inflammatory phase (0–7 days post‐injury), the initial clot releases TGF‐β, IGF‐I, and PDGF, recruiting neutrophils and monocytes. Macrophages subsequently clear necrotic debris while secreting trophic factors to activate fibroblast recruitment.^[^ ^196^, ^197^ ^]^ In the proliferative phase (7–21 days), fibroblasts migrate to the injury site, depositing disorganized fibrovascular scar tissue rich in type III collagen. By day 14, the remodeling phase initiates, characterized by cellular apoptosis, collagen realignment along stress lines, and gradual replacement of type III collagen with mechanically superior type I collagen via collagenase‐mediated resorption.^[^ ^198^ ^]^ Although tensile strength improves over months to years, healed tendons never fully recapitulate native properties due to persistent collagen cross‐linking deficiencies. Ligament healing mirrors tendon repair but suffers delayed revascularization, particularly in intra‐articular regions like the ACL, where synovial fluid inhibits vascular ingrowth. Both tissues’ regenerative capacity correlates strongly with local vascular density, underscoring angiogenesis as a critical therapeutic target.

#### 4.4.2 DNA‐Based Hydrogels in Tendon and Ligament Tissue Regeneration

DNA‐based hydrogels have emerged as a transformative strategy to overcome the limitations of conventional therapies.^[^ ^199^, ^200^ ^]^ Their unique combination of shear‐resistant mechanical properties and dynamic bioactivity addresses two critical needs: protecting cells during delivery and replicating native ECM microenvironments to sustain regeneration. A pioneering study by Ge et al.^[^ ^201^ ^]^ demonstrated this dual functionality in Achilles tendinopathy treatment. By encapsulating tendon stem/progenitor cells (TSPCs) within a DNA hydrogel matrix, the researchers created a 3D microenvironment that mimicked native tendon architecture (Figure 12B). The hydrogel's rigid double‐stranded DNA scaffold shielded TSPCs from shear forces during injection and tendon gliding, maintaining 96% cell viability over 72 h. In rat models, this approach outperformed free cell injections, achieving a 1.8‐fold increase in load‐to‐failure strength at 8 weeks alongside reduced inflammation and improved collagen I/III ratios. RNA sequencing further revealed enhanced ECM remodeling pathways, highlighting the hydrogel's ability to actively guide regenerative processes.

Despite these advances in tendon repair, DNA hydrogels remain untested in ligament regeneration. Ligaments such as the ACL require dynamic stiffness modulation far exceeding the capabilities of current DNA hydrogels. Additionally, intra‐articular environments demand materials that simultaneously resist synovial shear forces and promote angiogenesis.^[^ ^200^ ^]^

In conclusion, DNA‐based hydrogels represent a paradigm shift in tendon regeneration by recapitulating native ECM microenvironments and enabling precision cell delivery. In the future, DNA hydrogels should leverage their molecular programmability to engineer multifunctional systems. Hybrid networks incorporating synthetic polymers could enhance mechanical resilience for ligament repair, while integrating VEGF‐binding aptamers might stimulate angiogenesis in hypovascular regions. The programmable architecture and mechanical versatility of DNA‐based hydrogels lay the groundwork for potential expansion into ligament repair, though interdisciplinary collaboration remains essential to overcome translational barriers.

### 4.5 Neural Tissue Regeneration

Functional recovery of the musculoskeletal system critically depends on neural regeneration, as motor nerve reinnervation prevents irreversible muscle atrophy,^[^ ^202^ ^]^ while sensory nerve restoration maintains proprioception for joint stability.^[^ ^203^ ^]^ Clinical evidence indicates that nerve defects exceeding 5 cm result in permanent motor dysfunction unless neural regeneration strategies are integrated.^[^ ^204^ ^]^ Therefore, neural regeneration plays a pivotal role in musculoskeletal system restoration. DNA‐based hydrogels have emerged as promising candidates for neural regeneration due to their tunable mechanical properties, biocompatibility, and capacity to mimic ECM microenvironments while enabling precise biochemical functionalization. Their programmable nature allows the integration of neurotrophic factors, cell‐adhesive motifs, and stimuli‐responsive degradation, addressing critical challenges in nerve guidance and axonal regrowth.

**Figure 13:**
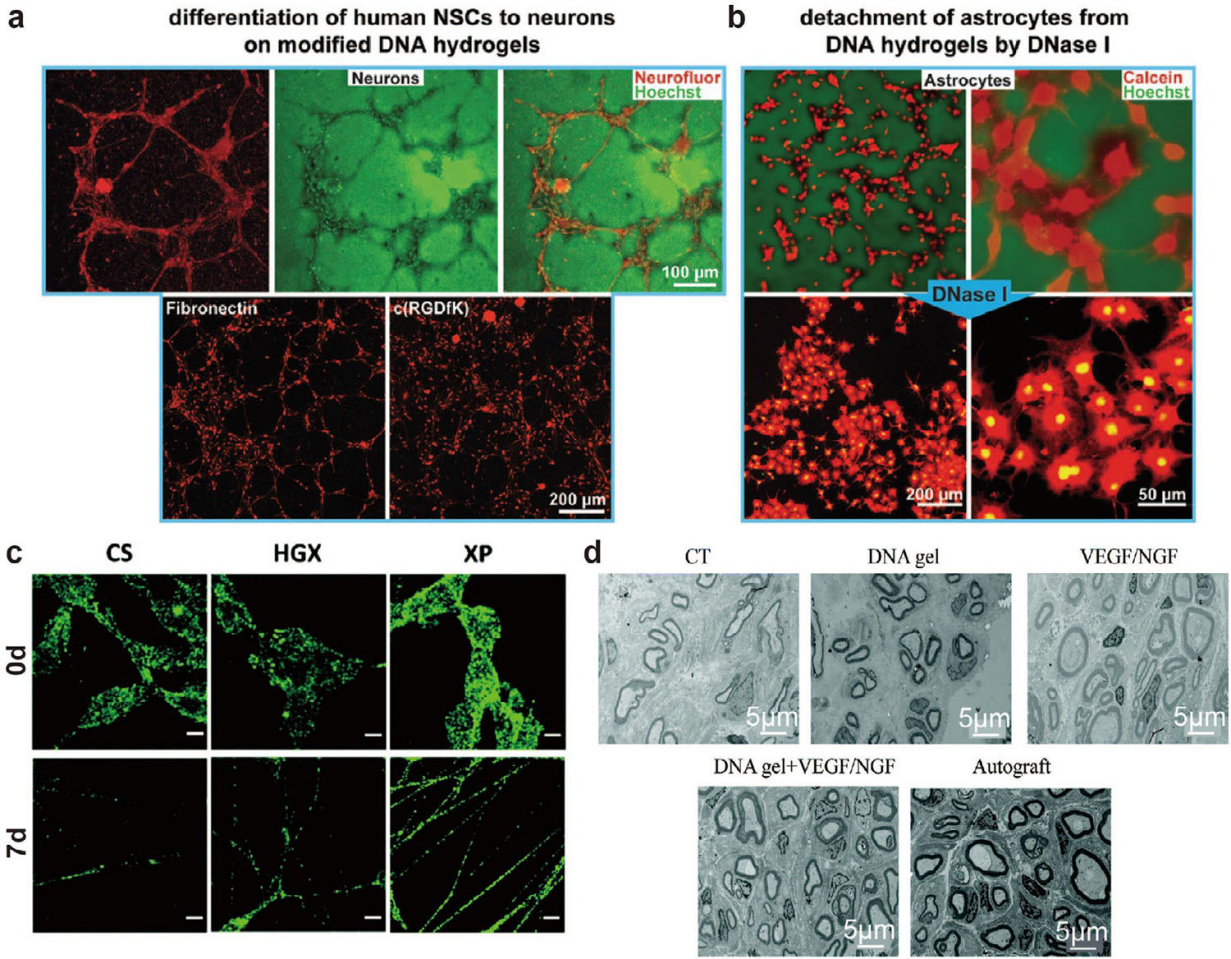
DNA‐based hydrogels in neural tissue regeneration. a,b) NSCs attachment and differentiation on DNase I‐modified DNA hydrogel. Reproduced with permission.^[^ ^205^ ^]^ Copyright 2019, Wiley. c) Immunofluorescence images of vinculin in SH‐SY5Y cells during differentiation. Reproduced with permission.^[^ ^46^ ^]^ Copyright 2022, RSC. d) TEM images of the regenerated sciatic nerves, with statistic analysis of the myelin sheath and myelinated nerve fibers. Reproduced with permission.^[^ ^41^ ^]^ Copyright 2021, RSC.

Recent studies highlight the versatility of DNA hydrogels in neural applications. Finke et al.^[^ ^205^ ^]^ developed a cost‐effective salmon sperm DNA hydrogel crosslinked with ethylene glycol diglycidyl ether (EGDE), enzymatically modified to incorporate cell‐adhesive motifs such as RGD peptides or antibodies. This scaffold supported neural stem cell attachment, survival, and differentiation into neurons under biochemical cues, while enabling gentle cell retrieval via DNase I‐mediated degradation (**Figure** **13**a,b). Its modular design allowed selective capture of specific cell populations underflow, demonstrating the potential for targeted neural repair. In another study, Hivare et al.^[^ ^46^ ^]^ functionalized DNA hydrogels with the laminin‐derived IKVAV peptide, significantly enhancing neuroblastoma cell differentiation, neurite elongation, and cytoskeletal dynamics compared to unmodified hydrogels. Single‐cell RNA sequencing revealed upregulated neural markers and mechanotransduction pathways, emphasizing the role of peptide‐DNA synergy in mimicking neurogenic ECM (Figure 13c).

For peripheral nerve injury (PNI), Liu et al.^[^ ^41^ ^]^ engineered an XT‐type DNA hydrogel loaded with VEGF and NGF, achieving biphasic release kinetics through differential degradation rates of X‐ and T‐type DNA units. In a 10 mm rat sciatic nerve defect model, this system outperformed hollow chitin conduits, showing improved electrophysiological recovery, myelination, and gastrocnemius muscle reinnervation, attributed to sustained neurotrophic signaling and angiogenesis (Figure 13d). Complementing this, Zhou et al.^[^ ^206^ ^]^ explored the univariate effects of hydrogel stiffness and degradation on neural progenitor cell (NPC) fate using enantiomeric D‐ and L‐DNA hydrogels. While stiffness alone (1‐3 kPa) did not alter differentiation, degradable D‐DNA hydrogels promoted cell‐cell interactions and Wnt/β‐catenin signaling via matrix remodeling, whereas non‐degradable L‐DNA hydrogels‐maintained NPC stemness, highlighting degradation‐driven mechanotransduction in neural commitment.

In summary, DNA hydrogels offer unparalleled advantages for neural regeneration, including dynamic stiffness modulation, spatiotemporal control over neurotrophic factor delivery, and customizable cell‐matrix interactions. Current innovations—such as enzyme‐responsive functionalization, peptide‐DNA hybrids, and biphasic release systems—demonstrate their potential to bridge gaps in nerve conduit design. Future efforts should focus on in vivo integration with bioelectronic interfaces and combinatorial delivery of genetic/mechanical cues to address complex neural pathologies.

### 4.6 DNA‐Based Hydrogels for Musculoskeletal Organoid Engineering

Organoids are 3D microstructures derived from stem or progenitor cells via directed differentiation, which are capable of self‐renewal and self‐organization.^[^ ^207^ ^]^ These organoids can replicate organ functions and act as living grafts to repair damaged tissues.^[^ ^208^ ^]^ Critically, musculoskeletal organoids demand precise recapitulation of dynamic mechanochemical microenvironments to guide self‐organization and tissue maturation. DNA‐based hydrogels offer unparalleled programmability to address this need through sequence‐directed control over viscoelasticity, degradation, and biofunctionalization. This section integrates advances in bone and cartilage organoid engineering, highlighting universal design principles enabled by DNA nanotechnology.

**Figure 14:**
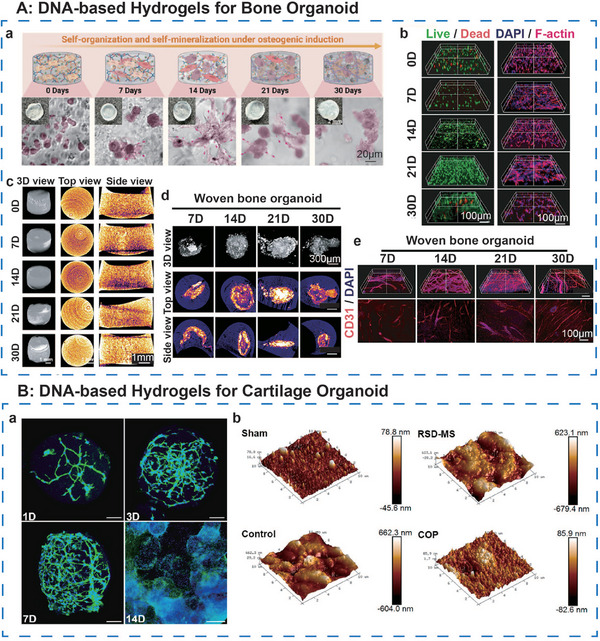
DNA‐based hydrogels for musculoskeletal organoid engineering. A, a) Schematic and macroscopic images of WBO development and morphology at different stages. b) Live/dead and cytoskeleton staining images and c) 3D reconstruction and cross‐sectional Micro‐CT images of organoids. d) 3D reconstruction and cross‐sectional Micro‐CT images of the ectopic bone tissue. e) Fluorescence staining images of the vascular network in the ectopic bone tissue. Reproduced with permission.^[^ ^211^ ^]^ Copyright 2025, Wiley. B, a) Fluorescence images of cocultured with RSD‐MS for 1, 3, 7 days (scale bar = 100 µm) and 14 days (scale bar = 500 µm). b) AFM images of cartilage surface after treatment for 10 weeks. Adapted under the terms of the CC BY‐NC‐ND license 4.0 from Ref. [213] Copyright 2024, KeAi Publishing.

Bone organoids have emerged as transformative tools for recapitulating the complex bone microenvironment, offering various advantages over traditional 2D cultures and animal models.^[^ ^209^ ^]^ By integrating patient‐derived cells, these 3D systems enable personalized disease modeling, drug screening, and mechanistic studies while preserving cell–cell and cell–matrix interactions critical for bone physiology.^[^ ^210^ ^]^ Zhu et al.^[^ ^211^ ^]^ pioneered a breakthrough in bone organoid engineering by developing a DNA‐GelMA dual‐network hydrogel (CGDE) tailored for dynamic bone regeneration (**Figure** **14**A). The hydrogel's dual‐network architecture combines DNA's injectability for minimally invasive delivery with a GelMA‐based scaffold providing load‐bearing capacity, mimicking trabecular bone mechanics while adapting to irregular defects. Key functional additives include EACP nanoparticles (biomimetic mitochondrial calcium phosphate) to amplify osteogenic differentiation and mineralization, and catalase conjugated to scavenge ROS, enhancing cell viability and vascularization. In vitro, BMSCs within CGDE were self‐organized into woven bone organoids (WBOs) within 21 days via intramembranous ossification. The hydrogel also enabled vascular compatibility, accelerating HUVECs migration and tubulogenesis. In vivo, 21‐day‐CGDE‐derived WBOs achieved the most rapid osseointegration in rat calvarial defects compared to other groups, with 45% BV/TV at 12 weeks (n = 6, p<0.001) and adaptive remodeling through DNA network degradation synchronized with osteoclast activity, promoting lamellar bone formation. This platform enables large‐scale organoid fabrication (cm‐scale constructs) and serves as a 4D model to study osteoblast‐osteoclast crosstalk and mineralization. However, challenges persist in long‐term stability and potential immune responses to DNA components, necessitating further optimization for clinical translation.

While bone organoids focus on mineralization and vascular integration, cartilage regeneration presents distinct challenges due to its avascular nature. Cartilage lacks vascular and neural innervation, which limits its regenerative potential.^[^ ^212^ ^]^ Consequently, the development of cartilage organoids has emerged as a promising frontier in cartilage repair, to mimic the 3D architecture and mechanical properties of native cartilage. Currently, Su et al.^[^ ^51^ ^]^ have pioneered the development of cartilage organoid precursors based on silk fibroin‐DNA hydrogels. However, bulk DNA‐SF hydrogels face challenges in solute diffusion efficiency, and are unsuitable for submicron‐scale applications. To address this, Su et al.^[^ ^213^ ^]^ introduced hydrogel microspheres, using microfluidic techniques to precisely control the formation of uniform‐sized SF‐DNA double‐network hydrogel microspheres (SD‐MSs) (Figure 14B). Further surface modification with RGD peptides led to the creation of RGD‐SF‐DNA hydrogel microspheres (RSD‐MSs), which enhanced the effective loading of BMSCs. Experimental results demonstrated that RSD‐MSs, by creating a high‐density 3D cell microenvironment similar to cartilage tissue, promoted intercellular material exchange and signal transmission. Transcriptomic analysis revealed that RSD‐MSs modulated integrin‐mediated cell adhesion and focal adhesion pathways, thereby promoting the biosynthesis of glycosaminoglycans (GAGs) and effectively inducing chondrogenic differentiation of BMSCs. In their study, hydrogel microspheres loaded with BMSCs and induced for 14 days to undergo chondrogenesis were defined as cartilage organoid precursors (COPs). In vivo experiments showed that these COPs exhibited superior cartilage repair capabilities and demonstrated the potential for long‐term cultivation of cartilage organoids.

These advances in tissue‐specific organoid engineering underscore a broader need, which requires a unified material platform capable of dynamically adapting to diverse musculoskeletal mechanobiology. This gap is addressed by programmable DNA matrice. Three‐dimensional cell and organoid cultures rely on mechanical support from viscoelastic matrices. However, conventional materials like Matrigel suffer from poor tunability and significant batch‐to‐batch variations.^[^ ^214^ ^]^ Capitalizing on the programmable advantages of DNA‐based hydrogels, Peng et al.^[^ ^78^ ^]^ developed a novel dynamic DNA‐crosslinked matrix (DyNAtrix) with precisely controllable properties as an organoid culture substrate. Beyond exhibiting intrinsic self‐healing properties common to DNA hydrogels, DyNAtrix uniquely enables independent tuning of stress relaxation and stiffness. By leveraging Combinatorial Crosslinker Libraries (CCLs), researchers can precisely modulate crosslinking efficiency, stiffness, and degradation behavior without altering DNA concentration or chemical composition, solely through adjustments in sequence library complexity. Furthermore, varying the length of overlap domains (nucleotide count) allows customization of stress relaxation dynamics, mimicking tissue‐specific mechanical properties, such as brain‐like fast relaxation and cartilage‐like slow relaxation. Supplementation with actin (50 µg mL^−1^) effectively inhibits DNase I in serum, maintaining matrix stability for over 48 hours. Experimental validation confirms DyNAtrix supports robust 3D culture of human mesenchymal stem cells (MSCs), human induced pluripotent stem cells (hiPSCs), and trophoblast organoids, sustaining long‐term development for up to 21 days. This platform establishes a precision biomimetic microenvironment for organoid engineering.

Future efforts should focus on multi‐tissue interfaces and patient‐specific genetic editing to advance precision medicine. Building on DyNAtrix's programmable mechanics and Su/Zhu's tissue‐specific designs, innovations in smart biomaterials may bridge these gaps by enabling real‐time monitoring of osteochondral gradients. As the field evolves, these DNA‐engineered platforms are poised to revolutionize our understanding of musculoskeletal pathologies.

## 5 Challenges and Limitations in Clinical Translation

While DNA‐based hydrogels demonstrate remarkable potential in musculoskeletal tissue regeneration, several critical challenges must be addressed to advance their clinical translation:

1. Imbalanced Mechanical Performances: A fundamental limitation lies in balancing dynamic responsiveness with structural stability. While physically crosslinked DNA hydrogels excel in shear‐thinning and self‐healing abilities through reversible interactions, their low mechanical strength restricts load‐bearing applications. In contrast, chemically crosslinked DNA hydrogels achieve higher stiffness but sacrifice adaptability. Although hybrid systems attempt to merge covalent and non‐covalent networks, unpredictable interactions between components occur, which highlights the need for innovative molecular design strategies.
2. Temporal Mismatch in Regeneration Dynamics: Most DNA hydrogels lack the dynamic adaptability to mirror phased healing processes. Their degradation kinetics rarely align with tissue regeneration timelines. Bone remodeling requires gradual stiffness reduction over months,^[^ ^215^ ^]^ while tendon repair demands initial mechanical support followed by controlled softening to prevent stress shielding.^[^ ^216^ ^]^ However, the rapid degradation behavior of pure DNA hydrogels may undermine bone defect repair, requiring months of mechanical support. Also, current static systems cannot autonomously adjust degradation rates or mechanical properties in response to microenvironmental cues, resulting in suboptimal tissue integration.
3. Safety Concerns: The clinical translation of DNA‐based hydrogels faces several significant safety concerns beyond their demonstrated short‐term biocompatibility. Genomic integration risks arise from potential incorporation of degraded DNA fragments into host genomes.^[^ ^217^ ^]^ Degradation byproducts introduce systemic toxicity uncertainties, including metabolic disturbances from nucleotide release and heavy metal accumulation in nanocomposite systems.^[^ ^218^ ^]^ Immune responses also remain a multifaceted challenge, as low selectivity for targeting tissue may cause severe systemic immunotoxicity,^[^ ^218^ ^]^ and unpredictable long‐term risks remain for using immunity‐adjustable nucleic acid drugs.^[^ ^219^ ^]^ Long‐term stability failures manifest through mechanical property degradation under physiological loads, bioactivity loss due to nuclease erosion, and unpredictable volume changes across implantation sites.^[^ ^220^ ^]^ While material engineering strategies show promise in mitigating these risks, critical knowledge gaps persist in chronic toxicity profiles and standardized safety assessment protocols. Comprehensive long‐term studies remain essential to address these barriers.
4. Scalability and Manufacturing Problems: Current synthesis methods limit practical implementation. Sequence‐specific architectures like Y+L/XT‐type hydrogels rely on costly solid‐phase oligonucleotide synthesis, restricting production to milligram scales. Hybrid systems incorporating nanomaterials face batch‐to‐batch variability, complicating quality control. Furthermore, for biomedical usage, sterilization faces challenges, as conventional gamma irradiation may disrupt delicate DNA secondary structures.
5. Standardization Deficits: The absence of unified evaluation protocols hinders clinical translation. For instance, essential parameters like payload encapsulation efficiency (EE%) and batch‐to‐batch reproducibility are frequently unreported. For experimental details, in vitro studies exhibit inconsistent methodologies, such as degradation metrics that vary across non‐comparable media, and mechanical tests lack uniform loading conditions. In vivo assessments rely on disparate animal models with non‐standardized endpoints, impeding cross‐study efficacy comparisons.
6. Unclear Failure Mechanisms in Pathological Microenvironments: Disease‐specific pathological microenvironments can significantly accelerate the failure mechanisms of DNA‐based hydrogels. For instance, inflammatory environments are characterized by heightened levels of ROS and upregulated MMP expression,^[^ ^221^, ^222^ ^]^ which can dramatically increase the degradation rate of hydrogel matrices incorporating protease‐sensitive components. Pathological conditions often involve alterations in pH, temperature, and immune signaling molecules,^[^ ^223^, ^224^, ^225^ ^]^ which collectively modulate the activity of nucleases, which critically impact the degradation kinetics of the DNA network within the hydrogel matrix. While these context‐specific failure risks are evident, the precise mechanisms governing hydrogel degradation across diverse pathological microenvironments remain largely unexplored, and effective strategies to counteract these limitations remain underexplored.

In summary, to address these challenges, future research must make interdisciplinary innovations to bridge the gap between laboratory promise and clinical reality.

## 6 Conclusion and Outlook

In conclusion, DNA‐based hydrogels represent a transformative frontier in musculoskeletal tissue regeneration, uniquely merging dynamic responsiveness, biofunctionality, and structural programmability. These materials transcend the limitations of conventional hydrogels, offering spatiotemporal control over cellular recruitment, differentiation, and extracellular matrix remodeling. The design strategies vary from pure DNA networks with shear‐thinning and self‐healing properties to hybrid systems combining synthetic/natural materials or DNA components, which enable tailored mechanical reinforcement, immunomodulation, and multi‐therapeutic delivery. Preclinical studies have demonstrated their potential in addressing critical challenges such as irregular defect filling, load‐bearing requirements, and chronic inflammation in musculoskeletal repair.

Looking ahead, their future development should combine advanced material innovations with clinical translation challenges. Future directions may focus on three key areas:

1. A promising direction involves leveraging DNA's programmability to advance personalized medicine, which tailors hydrogel compositions to patient‐specific genetic profiles, disease stages, or biomechanical demands.^[^ ^226^ ^]^
2. The integration of dynamic DNA networks with emerging technologies such as 3D bioprinting, wearable biosensors, and artificial intelligence could enable real‐time adaptive therapies,^[^ ^227^ ^]^ where hydrogels autonomously adjust stiffness, drug release kinetics, or immunomodulatory signals in response to changing tissue microenvironments.
3. Further exploration of hybrid systems combining synthetic DNA components may expand their applications in various fields like targeted gene editing.
4. Precision biomimetic structural engineering facilitates functional regeneration of the musculoskeletal system. For example, by mimicking the hierarchical “collagen‐hydroxyapatite” structure of natural bone tissue, researchers can design DNA framework‐guided gradient mineralization systems to achieve precise biomimetic mineralization.
5. For complex scenarios involving damage to multiple tissues, bone‐cartilage or tendon‐bone interfacing DNA‐based hydrogels can be developed. These hydrogels use precise spatial encoding of DNA strands and functional DNA components to guide the co‐cultivation of heterogeneous cell types, thereby reconstructing the ECM composition and biomechanical gradients in transitional zones.
6. Deep integration of gene editing technologies with DNA‐based hydrogels holds transformative potential. This includes combining CRISPR delivery systems to construct spatiotemporally controllable platforms for precise programming of tissue regeneration processes, as well as engineering hydrogels with epigenetic memory functionality to achieve sustained regulation of stem cell fate determination.

To fully realize this potential, efforts are needed to establish standardized quality control protocols for clinical‐grade DNA‐based hydrogel production, clarify long‐term biosafety profiles, and develop regulatory frameworks addressing the unique characteristics of DNA‐based biomaterials. As these challenges are overcome, DNA‐based hydrogels are poised to evolve from static implants to intelligent, adaptive partners in the body's regenerative processes, ushering in an era of truly smart and personalized musculoskeletal medicine.

## Conflict of Interest

The authors declare no conflict of interest.

## Author Contributions

R.S. was associated with validation, methodology, investigation, and visualization, and wrote the original draft. H.Z. was associated with validation, methodology, and conceptualization, and wrote the original draft. S.J. was associated with supervision, project administration, methodology, and reviewed and edited the final manuscript. K.L. and C.Y. were associated with supervision, project administration, methodology, funding acquisition, and conceptualization, and reviewed and edited the final manuscript.

## References

[1] A. M. Briggs , A. D. Woolf , K. Dreinhöfer , N. Homb , D. G. Hoy , D. Kopansky‐Giles , K. Åkesson , L. March , Bull. World Health Organ. 2018, 96, 366. 29875522 10.2471/BLT.17.204891 PMC5985424

[2] S. Safiri , A.‐A. Kolahi , M. Cross , C. Hill , E. Smith , K. Carson‐Chahhoud , M. A. Mansournia , A. Almasi‐Hashiani , A. Ashrafi‐Asgarabad , J. Kaufman , M. Sepidarkish , S. K. Shakouri , D. Hoy , A D. Woolf , L. March , G. Collins , R. Buchbinder , Arthritis Rheumatol. 2021, 73, 702. 33150702 10.1002/art.41571

[3] S. Haeusner , A. Jaukovic , E. Kupczyk , M. Herrmann , Am. J. Physiol. Cell Physiol. 2023, 324, C517. 36622067 10.1152/ajpcell.00482.2022

[4] I. Erezuma , T. Eufrasio‐da‐Silva , N. Golafshan , K. Deo , Y. K. Mishra , M. Castilho , A K. Gaharwar , S. Leeuwenburgh , A. Dolatshahi‐Pirouz , G. Orive , Adv. Healthcare Mater. 2021, 10, 2100217. 10.1002/adhm.202100217 34185438

[5] L. Roseti , V. Parisi , M. Petretta , C. Cavallo , G. Desando , I. Bartolotti , B. Grigolo , Mater. Sci. Eng. C Mater. Biol. Appl. 2017, 78, 1246. 28575964 10.1016/j.msec.2017.05.017

[6] T. E. Mroz , M. J. Joyce , M. P. Steinmetz , I. H. Lieberman , J. C. Wang , J. Am. Acad. Orthop. Surg. 2008, 16, 559. 18832599 10.5435/00124635-200810000-00001

[7] D. Ma , Y. Wang , W. Dai , Mater. Sci. Eng. C Mater. Biol. Appl. 2018, 89, 456. 29752118 10.1016/j.msec.2018.04.062

[8] B. Lv , Li Lu , L. Hu , P. Cheng , Y. Hu , X. Xie , G. Dai , B. Mi , X. Liu , G. Liu , Theranostics 2023, 13, 2015. 37064871 10.7150/thno.80615 PMC10091878

[9] R. Shi , Y. Zhu , Y. Chen , Y. Lin , S. Shi , J. Control Release 2024, 375, 155. 39242033 10.1016/j.jconrel.2024.09.004

[10] Y. Zhu , R. Shi , W. Lu , S. Shi , Y. Chen , Nanoscale 2024, 16, 7363. 38411498 10.1039/d3nr05844a

[11] F. Mo , K. Jiang , Di Zhao , Y. Wang , J. Song , W. Tan , Adv. Drug Deliv. Rev. 2021, 168, 79. 32712197 10.1016/j.addr.2020.07.018

[12] D. Wu , L. Wang , W. Li , X. Xu , W. Jiang , Int. J. Pharm. 2017, 533, 169. 28923770 10.1016/j.ijpharm.2017.09.032

[13] Bo Yang , Z. Zhao , Y. Pan , J. Xie , B. Zhou , Y. Li , Y. Dong , D. Liu , ACS Appl. Mater. Interfaces 2021, 13, 48414. 34633793 10.1021/acsami.1c15494

[14] S. Tanaka , K. Wakabayashi , K. Fukushima , S. Yukami , R. Maezawa , Y. Takeda , K. Tatsumi , Y. Ohya , A. Kuzuya , Chem. Asian J. 2017, 12, 2388. 28777486 10.1002/asia.201701066 PMC5639371

[15] E. Gilboa , A. Berezhnoy , B. Schrand , Cancer Immunol. Res. 2015, 3, 1195. 26541880 10.1158/2326-6066.CIR-15-0194

[16] X. Jian , X. Feng , Y. Luo , F. Li , J. Tan , Y. Yin , Y. Liu , Front. Bioeng. Biotechnol. 2021, 9, 661409. 34150729 10.3389/fbioe.2021.661409 PMC8206814

[17] D. Cao , Y. Xie , J. Song , Macromol. Rapid Commun. 2022, 43, 2200281. 10.1002/marc.202200281 35575627

[18] F. Li , J. Tang , J. Geng , D. Luo , D. Yang , Prog. Polym. Sci. 2019, 98, 101163.

[19] J. S. Kahn , Y. Hu , I. Willner , Acc. Chem. Res. 2017, 50, 680. 28248486 10.1021/acs.accounts.6b00542

[20] S. Nagahara , T. Matsuda , Polym. Gels Networks 1996, 4, 111.

[21] J. M. Isner , A. Pieczek , R. Schainfeld , R. Blair , L. Haley , T. Asahara , K. Rosenfield , S. Razvi , K. Walsh , J. F. Symes , Lancet 1996, 348, 370. 8709735 10.1016/s0140-6736(96)03361-2

[22] S Ho Um , J. B. Lee , N. Park , S. Y. Kwon , C C. Umbach , D. Luo , Nat. Mater. 2006, 5, 797. 16998469 10.1038/nmat1741

[23] J. B. Lee , S. Peng , D. Yang , Y. H. Roh , H. Funabashi , N. Park , E J. Rice , L. Chen , R. Long , M. Wu , D. Luo , Nat. Nanotechnol. 2012, 7, 816. 23202472 10.1038/nnano.2012.211

[24] T. Nöll , H. Schönherr , D. Wesner , M. Schopferer , T. Paululat , G. Nöll , Angew. Chem., Int. Ed. 2014, 53, 8328. 10.1002/anie.201402497 24965950

[25] Y. Katsumata , H. Kajiya , K. Okabe , T. Fukushima , T. Ikebe , Biochem. Biophys. Res. Commun. 2015, 468, 622. 26551467 10.1016/j.bbrc.2015.10.172

[26] H. Yang , H. Liu , H. Kang , W. Tan , J. Am. Chem. Soc. 2008, 130, 6320. 18444626 10.1021/ja801339w PMC2757630

[27] J. Yan , Z. Zhang , X. Zhan , K. Chen , Y. Pu , Y. Liang , B. He , Nanoscale 2021, 13, 9577. 33998643 10.1039/d1nr01155c

[28] S. Basu , A. R. Alkiswani , S. Pacelli , A. Paul , ACS Appl. Mater. Interfaces 2019, 11, 34621. 31483598 10.1021/acsami.9b10074 PMC7291362

[29] D. Wang , J. Cui , M. Gan , Z. Xue , J. Wang , P. Liu , Y. Hu , Y. Pardo , S. Hamada , D. Yang , D. Luo , J. Am. Chem. Soc. 2020, 142, 10114. 32392407 10.1021/jacs.0c02438

[30] N. Park , S. H. Um , H. Funabashi , J. Xu , D. Luo , Nat. Mater. 2009, 8, 432. 19329993 10.1038/nmat2419

[31] J. Song , M. Lee , T. Kim , J. Na , Y. Jung , G. Y. Jung , S. Kim , N. Park , Nat. Commun. 2018, 9, 4331. 30337586 10.1038/s41467-018-06864-0 PMC6193956

[32] M. Nishikawa , Y. Mizuno , K. Mohri , N. Matsuoka , S. Rattanakiat , Y. Takahashi , H. Funabashi , D. Luo , Y. Takakura , Biomaterials 2011, 32, 488. 20932569 10.1016/j.biomaterials.2010.09.013

[33] Y. Xing , E. Cheng , Y. Yang , P. Chen , T. Zhang , Y. Sun , Z. Yang , D. Liu , Adv. Mater. 2011, 23, 1117. 21181766 10.1002/adma.201003343

[34] S. Basu , S. Pacelli , Yi Feng , Q. Lu , J. Wang , A. Paul , ACS Nano 2018, 12, 9866. 30189128 10.1021/acsnano.8b02434 PMC6563937

[35] K. Zhang , V. W. Yam , Chem. Sci. 2020, 11, 3241. 34122831 10.1039/c9sc05910e PMC8157491

[36] R. Kurapati , U. V. Reddy , A. M. Raichur , N. Suryaprakash , J. Chem. Sci. 2016, 128, 325.

[37] C. Yao , R. Zhang , J. Tang , D. Yang , Nat. Protoc. 2021, 16, 5460. 34716450 10.1038/s41596-021-00621-2

[38] Y. Huang , W. Xu , G. Liu , L. Tian , Chem. Commun. 2017, 53, 3038. 10.1039/c7cc00636e 28239729

[39] J. Geng , C. Yao , X. Kou , J. Tang , D. Luo , D. Yang , Adv. Healthcare Mater. 2018, 7, 10.1002/adhm.201700998 29280301

[40] L. Zhou , W. Pi , S. Cheng , Z. Gu , K. Zhang , T. Min , W. Zhang , H. Du , P. Zhang , Y. Wen , Adv. Funct. Mater. 2021, 31, 2106167.

[41] S. Liu , Y. Liu , L. Zhou , Ci Li , M. Zhang , F. Zhang , Z. Ding , Y. Wen , P. Zhang , Biomater. Sci. 2021, 9, 8221. 34739533 10.1039/d1bm01377g

[42] Y. Miao , T. Lu , S. Cui , Z. Xu , X. Liu , Yu Zhang , J. Orthop. Translat. 2024, 49, 218. 39507323 10.1016/j.jot.2024.09.010 PMC11538604

[43] T. Noll , S. Wenderhold‐Reeb , H. Schonherr , G. Noll , Angew. Chem., Int. Ed. 2017, 56, 12004. 10.1002/anie.201705001 28597958

[44] Y. Miao , Y. Chen , J. Luo , X. Liu , Q. Yang , X. Shi , Y. Wang , Bioact. Mater. 2023, 21, 97. 36093326 10.1016/j.bioactmat.2022.08.005 PMC9417961

[45] L. Peng , W. Li , Ge Peng , D. Wei , L. Gou , Ye Zhou , Y. Zhou , X. Chen , L. Wu , W. Zhang , L. Hu , Qi Cao , C. Wang , Y. Zhang , Macromol. Rapid Commun. 2024, 45, 2300559. 10.1002/marc.202300559 38014713

[46] P. Hivare , A. Gangrade , G. Swarup , K. Bhavsar , A. Singh , R. Gupta , P. Thareja , S. Gupta , D. Bhatia , Nanoscale 2022, 14, 8611. 35687044 10.1039/d1nr07187d

[47] X. Jing , Si Wang , H. Tang , D. Li , F. Zhou , L. Xin , Q. He , S. Hu , T. Zhang , T. Chen , J. Song , ACS Appl. Mater. Interfaces 2022, 14, 16082. 35344325 10.1021/acsami.2c02278

[48] G. Creusen , C. O. Akintayo , K. Schumann , A. Walther , J. Am. Chem. Soc. 2020, 142, 16610. 32902960 10.1021/jacs.0c05488 PMC7612451

[49] G. Li , F. Gao , D. Yang , L. Lin , W. Yu , J. Tang , R. Yang , M. Jin , Y. Gu , P. Wang , E. Lu , Bioact. Mater. 2024, 42, 241. 39285909 10.1016/j.bioactmat.2024.08.035 PMC11404060

[50] Y. Miao , X. Liu , J. Luo , Q. Yang , Y. Chen , Y. Wang , Adv. Sci. 2024, 11, 2303637. 10.1002/advs.202303637 PMC10767401 37949678

[51] Z. Zhou , P. Song , Y. Wu , M. Wang , C. Shen , Z. Ma , X. Ren , X. Wang , X. Chen , Y. Hu , Z. Li , Q. Zhang , M. Li , Z. Geng , J. Su , Mater. Horiz. 2024, 11, 1465. 38221872 10.1039/d3mh01581e

[52] S. Basu , S. Pacelli , A. Paul , Acta Biomater. 2020, 105, 159. 31972367 10.1016/j.actbio.2020.01.021

[53] Q. Yang , Y. Miao , J. Luo , Y. Chen , Y. Wang , ACS Nano 2023, 17, 17131. 37585498 10.1021/acsnano.3c04816

[54] Y. Han , Y. Wu , F. Wang , G. Li , J. Wang , X. Wu , A. Deng , X. Ren , X. Wang , J. Gao , Z. Shi , L. Bai , J. Su , Bioact. Mater. 2024, 35, 1. 38298451 10.1016/j.bioactmat.2024.01.009 PMC10828543

[55] K. Patoine , K. Ta , A. Gilbert , M. Percuoco , A. E. Gerdon , Acta Biomater. 2024, 179, 234. 38554888 10.1016/j.actbio.2024.03.018

[56] J. Cao , J. Su , M. An , Y. Yang , Yi Zhang , J. Zuo , N. Zhang , Y. Zhao , Mol. Pharm. 2021, 18, 305. 33253580 10.1021/acs.molpharmaceut.0c00954

[57] M. Khvorostina , A. Mironov , I. Nedorubova , T. Bukharova , A. Vasilyev , D. Goldshtein , V. Komlev , V. Popov , Gels 2023, 9, 315. 37102926 10.3390/gels9040315 PMC10137500

[58] S. A. Cohen , O. Bar‐Am , C. Fuoco , G. Saar , C. Gargioli , D. Seliktar , Cell Death Dis. 2022, 13, 779. 36085138 10.1038/s41419-022-05166-0 PMC9463190

[59] S. Scalzo , A. K. Santos , H As Ferreira , P. A. Costa , P. H. Prazeres , N Ja da Silva , L. C. Guimarães , M De M e Silva , M Tr Rodrigues Alves , C Tr Viana , I Cg Jesus , A. P. Rodrigues , A. Birbrair , A. O. Lobo , F. Frézard , M. J. Mitchell , S. Guatimosim , P. P. G. Guimaraes , Int. J. Nanomed. 2022, 17, 2865. 10.2147/IJN.S366962 PMC9252585 35795081

[60] F. M. Fumasi , T. MacCulloch , J. Bernal‐Chanchavac , N. Stephanopoulos , J. L. Holloway , Biomater. Adv. 2024, 157, 213726. 38096646 10.1016/j.bioadv.2023.213726 PMC10842892

[61] G. Liu , Q. Lin , S. Jin , C. Gao , Mol. Cell 2022, 82, 333. 34968414 10.1016/j.molcel.2021.12.002

[62] C. Zhang , H. Huang , J. Chen , T. Zuo , Q. Ou , G. Ruan , J. He , C. Ding , ACS Appl. Mater. Interfaces 2023, 15, 16369. 36945078 10.1021/acsami.2c20496

[63] Yi He , En Ren , Z. Lu , H. Chen , Z. Qin , J. Wang , M. He , G. Liu , Li Zheng , J. Zhao , Nanomedicine 2020, 28, 102210. 32334102 10.1016/j.nano.2020.102210

[64] D. Athanasiadou , N. Meshry , N G. Monteiro , A C. Ervolino‐Silva , R. L. Chan , C A. McCulloch , R. Okamoto , K M. M. Carneiro , Proc. Natl. Acad. Sci. U. S. A. 2023, 120, 2220565120. 10.1073/pnas.2220565120 PMC10151614 37071684

[65] R. Ye , Z. Zhu , T. Gu , D. Cao , K. Jiang , Q. Dai , K. Xing , Y. Jiang , S. Zhou , P. Cai , D. T. Leong , M. Yu , J. Song , Nat. Commun. 2024, 15, 5557. 38956415 10.1038/s41467-024-49933-3 PMC11219873

[66] L. Wu , Y. Wang , X. Xu , Y. Liu , B. Lin , M. Zhang , J. Zhang , S. Wan , C. Yang , W. Tan , Chem. Rev. 2021, 121, 12035. 33667075 10.1021/acs.chemrev.0c01140

[67] C. Ji , J. Wei , L. Zhang , X. Hou , J. Tan , Q. Yuan , W. Tan , Chem. Rev. 2023, 123, 12471. 37931070 10.1021/acs.chemrev.3c00377

[68] J. Zhou , J. Rossi , Nat. Rev. Drug Discov. 2017, 16, 181. 27807347 10.1038/nrd.2016.199 PMC5700751

[69] N. Mor‐Vaknin , A. Saha , M. Legendre , C. Carmona‐Rivera , M. A. Amin , B J. Rabquer , M J. Gonzales‐Hernandez , J. Jorns , S. Mohan , S. Yalavarthi , D A. Pai , K. Angevine , S J. Almburg , J S. Knight , B S. Adams , A E. Koch , D A. Fox , D R. Engelke , M J. Kaplan , D M. Markovitz , Nat. Commun. 2017, 8, 14252. 28165452 10.1038/ncomms14252 PMC5303823

[70] R. Khandpur , C. Carmona‐Rivera , A. Vivekanandan‐Giri , A. Gizinski , S. Yalavarthi , J S. Knight , S. Friday , S. Li , R M. Patel , V. Subramanian , P. Thompson , P. Chen , D A. Fox , S. Pennathur , M J. Kaplan , Sci. Transl. Med. 2013, 5, 178ra140. 10.1126/scitranslmed.3005580 PMC3727661 23536012

[71] X. Wang , X. Zheng , Y. Duan , L. Ma , C. Gao , ACS Appl. Mater. Interfaces 2019, 11, 15170. 30942571 10.1021/acsami.9b03333

[72] D. Y. Zhang , G. Seelig , Nat. Chem. 2011, 3, 103. 21258382 10.1038/nchem.957

[73] C. Li , P. Chen , Yu Shao , Xu Zhou , Y. Wu , Z. Yang , Z. Li , T. Weil , D. Liu , Small 2015, 11, 1138. 25155469 10.1002/smll.201401906

[74] Y. Wang , Yu Shao , X. Ma , B. Zhou , A. Faulkner‐Jones , W. Shu , D. Liu , ACS Appl. Mater. Interfaces 2017, 9, 12311. 28300395 10.1021/acsami.7b01604

[75] X. Ding , Lu Fan , Li Wang , M. Zhou , Y. Wang , Y. Zhao , Mater. Horiz. 2023, 10, 3929. 37577809 10.1039/d3mh00891f

[76] X. Yan , Bo Yang , Y. Chen , Y. Song , J. Ye , Y. Pan , B. Zhou , Y. Wang , F. Mao , Y. Dong , D. Liu , J. Yu , Adv. Mater. 2021, 33, 2104758. 10.1002/adma.202104758 34657320

[77] Z. Tong , L. Jin , J. M. Oliveira , et al., Bioact. Mater. 2021, 6, 1375. 33210030 10.1016/j.bioactmat.2020.10.029 PMC7658331

[78] Yu‐H Peng , S‐Ku Hsiao , K. Gupta , A. Ruland , G K. Auernhammer , M F. Maitz , S. Boye , J. Lattner , C. Gerri , A. Honigmann , C. Werner , E. Krieg , Nat. Nanotechnol. 2023, 18, 1463. 37550574 10.1038/s41565-023-01483-3 PMC10716043

[79] O. Chaudhuri , L. Gu , D. Klumpers , M. Darnell , S A. Bencherif , J C. Weaver , N. Huebsch , H.‐P. Lee , E. Lippens , G N. Duda , D J. Mooney , Nat. Mater. 2016, 15, 326. 26618884 10.1038/nmat4489 PMC4767627

[80] O. Chaudhuri , J. Cooper‐White , P. A. Janmey , D. J. Mooney , V. B. Shenoy , Nature 2020, 584, 535. 32848221 10.1038/s41586-020-2612-2 PMC7676152

[81] H. Kamata , X. Li , U. I. Chung , T. Sakai , Adv. Healthcare Mater. 2015, 4, 2360. 10.1002/adhm.201500076 25939809

[82] C. Ghobril , M. W. Grinstaff , Chem. Soc. Rev. 2015, 44, 1820. 25649260 10.1039/c4cs00332b

[83] S. W. Kim , Y. H. Bae , T. Okano , Pharm. Res. 1992, 9, 283. 1614957 10.1023/a:1015887213431

[84] J. Xu , Q. Feng , S. Lin , W. Yuan , R. Li , J. Li , K. Wei , X. Chen , K. Zhang , Y. Yang , T. Wu , B. Wang , M. Zhu , R. Guo , G. Li , L. Bian , Biomaterials 2019, 210, 51. 31075723 10.1016/j.biomaterials.2019.04.031

[85] H. Kamata , Y. Akagi , Y. Kayasuga‐Kariya , U. I. Chung , T. Sakai , Science 2014, 343, 873. 24558157 10.1126/science.1247811

[86] H. Kamata , K. Kushiro , M. Takai , U. I. Chung , T. Sakai , Angew. Chem., Int. Ed. 2016, 55, 9282. 10.1002/anie.201602610 27320060

[87] W. Feng , Z. Wang , Adv. Sci. 2023, 10, 2303326. 10.1002/advs.202303326 PMC10558674 37544909

[88] X. Xu , V. V. Jerca , R. Hoogenboom , Mater. Horiz. 2021, 8, 1173. 34821910 10.1039/d0mh01514h

[89] J. Kim , J. Park , G. Choe , S‐In Jeong , H.‐S. Kim , J. Y. Lee , Adv. Healthcare Mater. 2024, 13, 2400142. 10.1002/adhm.202400142 38566357

[90] B. Yang , B. Zhou , C. Li , et al., Angew. Chem., Int. Ed. 2022, 61, 202202520.

[91] X. Du , Y. Xing , Y. Li , M. Cao , J. Wu , G. Dong , Z. Shi , X. Wei , M. Qiu , J. Gao , Y. Xu , H. Xu , D. Liu , Y. Dong , Macromol. Rapid Commun. 2024, 45, 2400177. 10.1002/marc.202400177 38636558

[92] K. H. Vining , D. J. Mooney , Nat. Rev. Mol. Cell Biol. 2017, 18, 728. 29115301 10.1038/nrm.2017.108 PMC5803560

[93] D. E. Discher , P. Janmey , Y. L. Wang , Science 2005, 310, 1139. 16293750 10.1126/science.1116995

[94] F. X. Jiang , B. Yurke , B. L. Firestein , N. A. Langrana , Ann. Biomed. Eng. 2008, 36, 1565. 18618260 10.1007/s10439-008-9530-z

[95] C. M. Madl , S. C. Heilshorn , H. M. Blau , Nature 2018, 557, 335. 29769665 10.1038/s41586-018-0089-z PMC6773426

[96] C. Lachance‐Brais , M. Rammal , J. Asohan , A. Katolik , X. Luo , D. Saliba , A. Jonderian , M J. Damha , M J. Harrington , H F. Sleiman , Adv. Sci. 2023, 10, 2205713. 10.1002/advs.202205713 PMC10131789 36752390

[97] T. Zhang , T. Tian , R. Zhou , S. Li , W. Ma , Y. Zhang , N. Liu , S. Shi , Q. Li , X. Xie , Y. Ge , M. Liu , Qi Zhang , S. Lin , X. Cai , Y. Lin , Nat. Protoc. 2020, 15, 2728. 32669637 10.1038/s41596-020-0355-z

[98] D. Costa , A. J. Valente , M. G. Miguel , J. Queiroz , Adv. Colloid Interface Sci. 2014, 205, 257. 24011472 10.1016/j.cis.2013.08.002

[99] C. F. Bennett , Annu. Rev. Med. 2019, 70, 307. 30691367 10.1146/annurev-med-041217-010829

[100] Z. Wang , H. Lu , T. Tang , L. Liu , B. Pan , J. Chen , D. Cheng , X. Cai , Yu Sun , F. Zhu , S. Zhu , Cell Prolif. 2022, 55, 13316. 10.1111/cpr.13316 PMC9628242 35869570

[101] S. Lin , Qi Zhang , S. Li , T. Zhang , L. Wang , X. Qin , M. Zhang , S. Shi , X. Cai , ACS Appl. Mater. Interfaces 2020, 12, 11397. 32083455 10.1021/acsami.0c00874

[102] Y. Zhao , S. Li , M. Feng , M. Zhang , Z. Liu , Y. Yao , T. Zhang , Y. Jiang , Y. Lin , X. Cai , Small 2023, 19, 2302326. 10.1002/smll.202302326 37317020

[103] L. Fu , P. Li , J. Zhu , Z. Liao , C. Gao , H. Li , Z. Yang , T. Zhao , W. Chen , Y. Peng , F. Cao , C. Ning , X. Sui , Q. Guo , Y. Lin , S. Liu , Bioact. Mater. 2022, 9, 411. 34820580 10.1016/j.bioactmat.2021.07.028 PMC8586787

[104] F. Squadrito , A. Bitto , N. Irrera , G. Pizzino , G. Pallio , L. Minutoli , D. Altavilla , Front. Pharmacol. 2017, 8, 224. 28491036 10.3389/fphar.2017.00224 PMC5405115

[105] B. Balasubramanian , W. K. Pogozelski , T. D. Tullius , Proc. Natl. Acad. Sci. U. S. A. 1998, 95, 9738. 9707545 10.1073/pnas.95.17.9738 PMC21406

[106] Z. Wang , W. Li , L. Gou , Ye Zhou , Ge Peng , J. Zhang , J. Liu , R. Li , H. Ni , W. Zhang , T. Cao , Qi Cao , H. Su , Y.‐P. Han , N. Tong , X. Fu , E. Ilegems , Y. Lu , P.‐O. Berggren , X. Zheng , C. Wang , Adv. Healthcare Mater. 2022, 11, 2200782. 10.1002/adhm.202200782 36101484

[107] N. Korolev , A. P. Lyubartsev , A. Rupprecht , L. Nordenskiold , Biophys. J. 1999, 77, 2736. 10545373 10.1016/s0006-3495(99)77107-9 PMC1300547

[108] J. Tang , J. Wang , J. Ou , Z. Cui , C. Yao , D. Yang , Small Methods 2024, 8, 2301236. 10.1002/smtd.202301236 38351479

[109] R. Shi , Y. Zhu , W. Lu , Y. Shao , Y. Chen , Mi Zhou , Y. Lin , S. Shi , Chin. Chem. Lett. 2025, 36, 110241.

[110] S. He , J. Fang , C. Zhong , M. Wang , F. Ren , Adv. Healthcare Mater. 2022, 11, 2201096. 10.1002/adhm.202201096 35971854

[111] Mi Zhou , N. Liu , Qi Zhang , T. Tian , Q. Ma , T. Zhang , X. Cai , Cell Prolif. 2019, 52, 12566. 10.1111/cpr.12566 PMC6536416 30883969

[112] J. Hernigou , P. Vertongen , J. Rasschaert , P. Hernigou , Int. J. Mol. Sci. 2021, 22, 3844. 33917689 10.3390/ijms22083844 PMC8068069

[113] W. Wang , K. W. K. Yeung , Bioact. Mater. 2017, 2, 224. 29744432 10.1016/j.bioactmat.2017.05.007 PMC5935655

[114] N. Kumar , K. G. Lopez , S. Alathur Ramakrishnan , J. T. P. D. Hallinan , J. Y. H. Fuh , N. Pandita , S. Madhu , A. Kumar , L M. Benneker , B A. Vellayappan , Radiother. Oncol. 2021, 163, 93. 34419506 10.1016/j.radonc.2021.08.007

[115] R. Florencio‐Silva , G. R. Sasso , E. Sasso‐Cerri , M. J. Simoes , P. S. Cerri , Biomed. Res. Int. 2015, 2015, 421746. 26247020 10.1155/2015/421746 PMC4515490

[116] Y. Xiong , B. B. Mi , Z. Lin , Y. Q. Hu , L. Yu , K. K. Zha , A. C. Panayi , T. Yu , L. Chen , Z. P. Liu , A. Patel , Q. Feng , S. H. Zhou , G. H. Liu , Mil. Med. Res. 2022, 9, 65. 36401295 10.1186/s40779-022-00426-8 PMC9675067

[117] A. Salhotra , H. N. Shah , B. Levi , M. T. Longaker , Nat. Rev. Mol. Cell Biol. 2020, 21, 696. 32901139 10.1038/s41580-020-00279-w PMC7699981

[118] L. J. Raggatt , N. C. Partridge , J. Biol. Chem. 2010, 285, 25103. 20501658 10.1074/jbc.R109.041087 PMC2919071

[119] He Hu , Z. Li , M. Lu , X. Yun , W. Li , C. Liu , Ai Guo , Biomed. Pharmacother. 2018, 105, 66. 29843046 10.1016/j.biopha.2018.05.051

[120] W. Mu , G. Liang , Y. Feng , Y. Jiang , F. Qu , Pharmaceuticals 2022, 15, 1274. 36297386 10.3390/ph15101274 PMC9611301

[121] J. Duguid , V. A. Bloomfield , J. Benevides , G. J. Thomas Jr. , Biophys. J. 1993, 65, 1916. 8298021 10.1016/S0006-3495(93)81263-3 PMC1225927

[122] N. C. Seeman , Annu. Rev. Biophys. Biomol. Struct. 1998, 27, 225. 9646868 10.1146/annurev.biophys.27.1.225

[123] D. Athanasiadou , K. M. M. Carneiro , Nat. Rev. Chem. 2021, 5, 93. 37117611 10.1038/s41570-020-00242-5

[124] X. Liu , X. Jing , Pi Liu , M. Pan , Z. Liu , X. Dai , J. Lin , Q. Li , F. Wang , S. Yang , L. Wang , C. Fan , Chem 2020, 6, 472.

[125] Y. Xu , K. C. H. Tijssen , P. H. H. Bomans , A. Akiva , H. Friedrich , A P. M. Kentgens , N. A. J. M. Sommerdijk , Nat. Commun. 2018, 9, 2582. 29968713 10.1038/s41467-018-05006-w PMC6030133

[126] F. Kim , T. Chen , T. Burgess , P. Rasie , T. L. Selinger , A. Greschner , G. Rizis , K. Carneiro , Chem. Sci. 2019, 10, 10537. 32055376 10.1039/c9sc02811k PMC6988742

[127] F. Wegman , R. E. Geuze , Y. J. van der Helm , F. Cumhur Öner , W. J. A. Dhert , J. Alblas , J. Tissue Eng. Regen. Med. 2014, 8, 763. 22888035 10.1002/term.1571

[128] M. Zhou , N.‐X. Liu , S.‐R. Shi , Y. Li , Q. Zhang , Q.‐Q. Ma , T.‐R. Tian , W.‐J. Ma , X.‐X. Cai , Y.‐F. Lin , Nanomedicine 2018, 14, 1227. 29458214 10.1016/j.nano.2018.02.004

[129] H. Lee , S. Hwa , S. Cho , J. H. Kim , H. J. Song , Y. Ko , J. B. Park , Medicina (Kaunas) 2024, 60, 10.3390/medicina60101610 PMC11509416 39459397

[130] A. Sato , H. Kajiya , N. Mori , H. Sato , T. Fukushima , H. Kido , J. Ohno , PLoS One 2017, 12, 0169522. 10.1371/journal.pone.0169522 PMC5218488 28060874

[131] J. Park , H. Elmlund , P. Ercius , J. M. Yuk , D T. Limmer , Q. Chen , K. Kim , S. H. Han , D. A. Weitz , A. Zettl , A. P. Alivisatos , Science 2015, 349, 290. 26185247 10.1126/science.aab1343

[132] T. Lu , X. Liu , S. Qian , H. Cao , Y. Qiao , Y. Mei , P. K. Chu , C. Ding , Biomaterials 2014, 35, 5731. 24767786 10.1016/j.biomaterials.2014.04.003

[133] M. Baig , W. L. Dissanayaka , C. Zhang , Int. J. Biol. Macromol. 2021, 188, 657. 34371047 10.1016/j.ijbiomac.2021.07.198

[134] I. D'Alimonte , E. Nargi , A. Lannutti , M. Marchisio , L. Pierdomenico , G. Costanzo , P Di Iorio , P. Ballerini , P. Giuliani , F. Caciagli , R. Ciccarelli , Stem Cell Res. 2013, 11, 611. 23651584 10.1016/j.scr.2013.04.002

[135] B. A. Evans , C. Elford , A. Pexa , K. Francis , A. C. Hughes , A. Deussen , J. Ham , J. Bone Miner. Res. 2006, 21, 228. 16418778 10.1359/JBMR.051021

[136] P. Chen , Q. Ruan , R. Nasseri , H. Zhang , X. Xi , H. Xia , G. Xu , Q. Xie , C. Yi , Z. Sun , H. Shahsavan , W. Zhang , Adv. Sci. 2022, 9, 2204730. 10.1002/advs.202204730 PMC9731706 36253140

[137] Y. Li , R. Fu , Z. Duan , C. Zhu , D. Fan , ACS Nano 2022, 16, 7486. 35533294 10.1021/acsnano.1c10575

[138] M. N. Moghadam , V. Kolesov , A. Vogel , H. A. Klok , D. P. Pioletti , Biomaterials 2014, 35, 450. 24112806 10.1016/j.biomaterials.2013.09.065

[139] A. J. Johnson , P. R. Goger , W. S. Tillett , J. Clin. Invest. 1954, 33, 1670. 13211824 10.1172/JCI103048 PMC1072599

[140] J. Gacanin , A. Kovtun , S. Fischer , V. Schwager , J. Quambusch , S. L. Kuan , W. Liu , F. Boldt , C. Li , Z. Yang , D. Liu , Y. Wu , T. Weil , H. Barth , A. Ignatius , Adv. Healthcare Mater. 2017, 6, 1700392. 10.1002/adhm.201700392 28758712

[141] G. Peng , W. Li , L. Peng , R. Li , Z. Wang , Y. Zhou , L. Gou , X. Zhu , Q. Xie , X. Zhang , S. Shen , L. Wu , L. Hu , C. Wang , X. Zheng , N. Tong , Small 2024, 20, 2305594. 10.1002/smll.202305594 37919857

[142] R. S. Weinstein , Endocrinol. Metab. Clin. North Am. 2012, 41, 595. 22877431 10.1016/j.ecl.2012.04.004 PMC3417039

[143] J. Li , D. J. Mooney , Nat. Rev. Mater. 2016, 1, 16071. 29657852 10.1038/natrevmats.2016.71 PMC5898614

[144] S. M. Chim , J. Tickner , S To Chow , V. Kuek , B. Guo , Ge Zhang , V. Rosen , W. Erber , J. Xu , Cytokine Growth Factor Rev. 2013, 24, 297. 23611723 10.1016/j.cytogfr.2013.03.008

[145] M. L. Brandi , P. Collin‐Osdoby , J. Bone Miner. Res. 2006, 21, 183. 16418774 10.1359/JBMR.050917

[146] J. Whitehead , K H. Griffin , M. Gionet‐Gonzales , C E. Vorwald , S E. Cinque , J. K Leach , Biomaterials 2021, 269, 120607. 33385687 10.1016/j.biomaterials.2020.120607 PMC7870573

[147] S. Henderson , I. Ibe , S. Cahill , Y. H. Chung , F. Y. Lee , J. Bone Joint Surg. Am. 2019, 101, 1399. 31393433 10.2106/JBJS.18.01297

[148] V. V. Shanbhogue , S. Hansen , M. Frost , K. Brixen , A. P. Hermann , Lancet Diabetes Endocrinol. 2017, 5, 827. 28546096 10.1016/S2213-8587(17)30134-1

[149] L. Gilbert , X. He , P. Farmer , S. Boden , M. Kozlowski , J. Rubin , M S. Nanes , Endocrinology 2000, 141, 3956. 11089525 10.1210/endo.141.11.7739

[150] F Q. Bui , C. L. C. Almeida‐da‐Silva , B. Huynh , A. Trinh , J. Liu , J. Woodward , H. Asadi , D M. Ojcius , Biomed. J. 2019, 42, 27. 30987702 10.1016/j.bj.2018.12.001 PMC6468093

[151] W. Li , C. Wang , Z. Wang , L. Gou , Ye Zhou , Ge Peng , M. Zhu , J. Zhang , R. Li , H. Ni , L. Wu , W. Zhang , J. Liu , Y. Tian , Z. Chen , Y.‐P. Han , N. Tong , X. Fu , X. Zheng , P.‐O. Berggren , ACS Appl. Mater. Interfaces 2022, 14, 25173. 35638566 10.1021/acsami.2c04769

[152] H. Muir , BioEssays 1995, 17, 1039. 8634065 10.1002/bies.950171208

[153] W. Wei , Y. Ma , X. Yao , W. Zhou , X. Wang , C. Li , J. Lin , Q. He , S. Leptihn , H. Ouyang , Bioact. Mater. 2021, 6, 998. 33102942 10.1016/j.bioactmat.2020.09.030 PMC7557878

[154] Y. Krishnan , A. J. Grodzinsky , Matrix Biol. 2018, 71‐72, 51. 10.1016/j.matbio.2018.05.005 PMC6146013 29803938

[155] A. R. Armiento , M. J. Stoddart , M. Alini , D. Eglin , Acta Biomater. 2018, 65, 1. 29128537 10.1016/j.actbio.2017.11.021

[156] E. C. Beck , M. Barragan , M. H. Tadros , S. H. Gehrke , M. S. Detamore , Acta Biomater. 2016, 38, 94. 27090590 10.1016/j.actbio.2016.04.019 PMC4903909

[157] S. Batool , B. J. Roth , Y. Xia , Materials 2024, 17, 238. 38204091 10.3390/ma17010238 PMC10779946

[158] J. A. Buckwalter , J. Orthop. Sports Phys. Ther. 1998, 28, 192. 9785255 10.2519/jospt.1998.28.4.192

[159] W. S. Toh , M. Brittberg , J. Farr , C. B. Foldager , A. H. Gomoll , J H Po Hui , J. B. Richardson , S. Roberts , M. Spector , Acta Orthop. 2016, 87, 6. 27658487 10.1080/17453674.2016.1235087 PMC5389431

[160] M. Li , H. Yin , Z. Yan , H. Li , J. Wu , Y. Wang , Fu Wei , G. Tian , C. Ning , H. Li , C. Gao , L. Fu , S. Jiang , M. Chen , X. Sui , S. Liu , Z. Chen , Q. Guo , Acta Biomater. 2022, 140, 23. 34896634 10.1016/j.actbio.2021.12.006

[161] R. F. Loeser , S. R. Goldring , C. R. Scanzello , M. B. Goldring , Arthritis Rheum. 2012, 64, 1697. 22392533 10.1002/art.34453 PMC3366018

[162] L. Liu , W. Zhang , T. Liu , Y. Tan , C. Chen , J. Zhao , H. Geng , C. Ma , Redox Biol. 2023, 62, 102663. 36924682 10.1016/j.redox.2023.102663 PMC10026041

[163] H. Li , Y. Yuan , L. Zhang , C. Xu , H. Xu , Z. Chen , Adv. Sci. 2024, 11, 2305363. 10.1002/advs.202305363 PMC10916582 38093659

[164] I. Matai , G. Kaur , A. Seyedsalehi , A. McClinton , C. T. Laurencin , Biomaterials 2020, 226, 119536. 31648135 10.1016/j.biomaterials.2019.119536

[165] S. Jahn , J. Seror , J. Klein , Annu. Rev. Biomed. Eng. 2016, 18, 235. 27420572 10.1146/annurev-bioeng-081514-123305

[166] S. Shi , Q. Peng , X. Shao , J. Xie , S. Lin , T. Zhang , Q. Li , X. Li , Y. Lin , ACS Appl. Mater. Interfaces 2016, 8, 19353. 27403707 10.1021/acsami.6b06528

[167] W. Ma , X. Shao , D. Zhao , Q. Li , M. Liu , T. Zhou , X. Xie , C. Mao , Y. Zhang , Y. Lin , ACS Appl. Mater. Interfaces 2018, 10, 7892. 29424522 10.1021/acsami.8b00833

[168] S. Sirong , C. Yang , T. Taoran , Li Songhang , L. Shiyu , Z. Yuxin , S. Xiaoru , Z. Tao , L. Yunfeng , C. Xiaoxiao , Bone Res. 2020, 8, 6. 32047705 10.1038/s41413-019-0077-4 PMC7010777

[169] X. Gao , Z. Xu , G. Liu , J. Wu , Acta Biomater. 2021, 119, 57. 33166714 10.1016/j.actbio.2020.11.004

[170] P. Li , L. Fu , Z. Liao , Yu Peng , C. Ning , C. Gao , D. Zhang , X. Sui , Y. Lin , S. Liu , C. Hao , Q. Guo , Biomaterials 2021, 278, 121131. 34543785 10.1016/j.biomaterials.2021.121131

[171] D R. Chery , B. Han , Y. Zhou , C. Wang , S M. Adams , P. Chandrasekaran , B. Kwok , Su‐J Heo , M. Enomoto‐Iwamoto , X. L Lu , D. Kong , R V. Iozzo , D E. Birk , R L. Mauck , L. Han , Matrix Biol. 2021, 96, 1. 33246102 10.1016/j.matbio.2020.11.002 PMC7902451

[172] N H. Varady , T B. Amen , P F. Abraham , A. Chopra , D M. Freccero , E L. Smith , S D. Martin , Am. J. Sports Med. 2021, 49, 2482. 34161174 10.1177/03635465211022798

[173] Y. Chen , B. Bilgen , R A. Pareta , A J. Myles , H. Fenniri , D M. Ciombor , R K. Aaron , T J. Webster , Tissue Eng. Part C Methods 2010, 16, 1233. 20184414 10.1089/ten.TEC.2009.0400

[174] A. L. Chun , J. G. Moralez , T. J. Webster , H. Fenniri , Biomaterials 2005, 26, 7304. 16023193 10.1016/j.biomaterials.2005.05.080

[175] C. H. Kim , M. S. Khil , H. Y. Kim , H. U. Lee , K. Y. Jahng , J. Biomed. Mater. Res. B Appl. Biomater. 2006, 78, 283. 16362963 10.1002/jbm.b.30484

[176] Y. Peng , S. Liang , Q.‐F. Meng , D. Liu , K. Ma , M. Zhou , K. Yun , L. Rao , Z. Wang , Adv. Mater. 2024, 36, 2313188. 10.1002/adma.202313188 38362813

[177] T. Vinardell , E. J. Sheehy , C. T. Buckley , D. J. Kelly , Tissue Eng. Part A 2012, 18, 1161. 22429262 10.1089/ten.tea.2011.0544 PMC3360504

[178] T. Gonzalez‐Fernandez , E. G. Tierney , G. M. Cunniffe , F. J. O'Brien , D. J. Kelly , Tissue Eng. Part A 2016, 22, 776. 27079852 10.1089/ten.TEA.2015.0576

[179] S. H. Kim , Y. P. Djaja , Y.‐B. Park , J.‐G. Park , Y.‐B. Ko , C.‐W. Ha , Am. J. Sports Med. 2020, 48, 2839. 31874044 10.1177/0363546519892278

[180] P. Wang , Z. Zhao , Z. Li , X. Li , B. Huang , X. Lu , S. Dai , S. Li , Z. Man , W. Li , J. Nanobiotechnology 2024, 22, 325. 38858695 10.1186/s12951-024-02608-z PMC11163801

[181] A. Defois , N. Bon , S. Sourice , M. Mével , M. Georget , B. Bodic , O. Adjali , Y. Maugars , J. Guicheux , C. Vinatier , Osteoarthritis Cartilage 2021, 29, S205.

[182] D. J. Hunter , S. Bierma‐Zeinstra , Lancet 2019, 393, 1745. 31034380 10.1016/S0140-6736(19)30417-9

[183] W. Ji , X. Li , M. Xiao , Y. Sun , W. Lai , H. Zhang , H. Pei , L. Li , Chemistry 2021, 27, 8745. 33778987 10.1002/chem.202100149

[184] Y. Shao , H. Jia , T. Cao , D. Liu , Acc. Chem. Res. 2017, 50, 659. 28299927 10.1021/acs.accounts.6b00524

[185] X. Dai , Z. Zhu , Y. Li , B. Yang , J. F. Xu , Y. Dong , X. Zhou , L. T. Yan , D. Liu , J. Am. Chem. Soc. 2022, 144, 19017. 36197334 10.1021/jacs.2c07830

[186] X. Valle , E. Alentorn‐Geli , J L. Tol , B. Hamilton , W E. Garrett , R. Pruna , L. Til , J. A. Gutierrez , X. Alomar , R. Balius , N. Malliaropoulos , J. C. Monllau , R. Whiteley , E. Witvrouw , K. Samuelsson , G. Rodas , Sports Med. 2017, 47, 1241. 27878524 10.1007/s40279-016-0647-1

[187] T. A. Jarvinen , T. L. Jarvinen , M. Kaariainen , H. Kalimo , M. Jarvinen , Am. J. Sports Med. 2005, 33, 745. 15851777 10.1177/0363546505274714

[188] A. J. Engler , S. Sen , H. L. Sweeney , D. E. Discher , Cell 2006, 126, 677. 16923388 10.1016/j.cell.2006.06.044

[189] C. Philips , L. Terrie , L. Thorrez , Biomaterials 2022, 283, 121436. 35248912 10.1016/j.biomaterials.2022.121436

[190] J. Li , W. Zhang , A. Liu , Y. Lu , L. Yu , X. Liu , L. Sun , B. Zhao , X. Tong , T. Liu , Y. Liu , Adv. Funct. Mater. 2024, 34, 2401564.

[191] In G Kim , Mi Ri Park , Y. H. Choi , Ji S Choi , H.‐J. Ahn , S. K. Kwon , J Ho Lee , ACS Biomater. Sci. Eng. 2019, 5, 1497. 33405624 10.1021/acsbiomaterials.8b01541

[192] B. Gharaibeh , Y. Chun‐Lansinger , T. Hagen , S. J. M. Ingham , V. Wright , F. Fu , J. Huard , Birth Defects Res. C Embryo Today 2012, 96, 82. 22457179 10.1002/bdrc.21005 PMC4360899

[193] D. Jaiswal , L. Yousman , M. Neary , E. Fernschild , B. Zolnoski , S. Katebifar , S. Rudraiah , A. D. Mazzocca , S. G. Kumbar , Biomed. Mater. 2020, 15, 052001. 32235051 10.1088/1748-605X/ab852f

[194] N. L. Leong , J. L. Kator , T. L. Clemens , A. James , M. Enamoto‐Iwamoto , J. Jiang , J. Orthop. Res. 2020, 38, 7. 31529731 10.1002/jor.24475 PMC7307866

[195] T. Molloy , Y. Wang , G. Murrell , Sports Med. 2003, 33, 381. 12696985 10.2165/00007256-200333050-00004

[196] G. Yang , B. B. Rothrauff , R. S. Tuan , Birth Defects Res. C Embryo Today 2013, 99, 203. 24078497 10.1002/bdrc.21041 PMC4041869

[197] A. Bedi , T. Maak , C. Walsh , S A. Rodeo , D. Grande , D M. Dines , J S. Dines , J. Shoulder Elbow Surg. 2012, 21, 218. 22244065 10.1016/j.jse.2011.09.020

[198] M. Hope , T. S. Saxby , Foot Ankle Clin. 2007, 12, 553. 17996614 10.1016/j.fcl.2007.07.003

[199] C. Darrieutort‐Laffite , F. Blanchard , L. J. Soslowsky , B. Le Goff , Joint Bone Spine 2024, 91, 105696. 38307405 10.1016/j.jbspin.2024.105696

[200] Y. Sun , R. Sheng , Z. Cao , C. Liu , J. Li , Po Zhang , Y. Du , Q. Mo , Q. Yao , J. Chen , W. Zhang , Sci. Adv. 2024, 10, adm7164. 10.1126/sciadv.adm7164 PMC11042749 38657071

[201] Z. Ge , W. Li , R. Zhao , W. Xiong , D. Wang , Y. Tang , Q. Fang , X. Deng , Z. Zhang , Y. Zhou , X. Chen , Y. Li , Y. Lu , C. Wang , G. Wang , Small 2023, 19, 2207231. 10.1002/smll.202207231 37066733

[202] M. Siemionow , G. Brzezicki , Int. Rev. Neurobiol. 2009, 87, 141. 19682637 10.1016/S0074-7742(09)87008-6

[203] T. Gordon , J. Physiol. 2016, 594, 3517. 27365158 10.1113/JP270898 PMC4929322

[204] D. Grinsell , C. P. Keating , Biomed Res. Int. 2014, 2014, 698256. 25276813 10.1155/2014/698256 PMC4167952

[205] A. Finke , A. K. Schneider , A. S. Spreng , et al., Adv. Healthcare Mater. 2019, 8, 1900080.

[206] B. Zhou , Bo Yang , Q. Liu , Lu Jin , Yu Shao , T. Yuan , Ya‐N Zhang , C. Wang , Z. Shi , X. Li , Y. Pan , N. Qiao , J.‐F. Xu , Y. R. Yang , Y. Dong , L. Xu , S. Gui , D. Liu , J. Am. Chem. Soc. 2023, 145, 8954. 37029734 10.1021/jacs.2c13373

[207] M. A. Lancaster , J. A. Knoblich , Science 2014, 345, 1247125. 25035496 10.1126/science.1247125

[208] T. Takebe , J. M. Wells , Science 2019, 364, 956. 31171692 10.1126/science.aaw7567 PMC8212787

[209] S. Chen , X. Chen , Z. Geng , J. Su , Bioact. Mater. 2022, 18, 15. 35387160 10.1016/j.bioactmat.2022.01.048 PMC8961298

[210] D. Janagama , S. K. Hui , Materials 2020, 13, 5609. 33316977 10.3390/ma13245609 PMC7763362

[211] M. Zhu , H. Zhang , Q. Zhou , S. Sheng , Q. Gao , Z. Geng , X. Chen , Y. Lai , Y. Jing , Ke Xu , L. Bai , G. Wang , J. Wang , Y. Jiang , J. Su , Adv. Mater. 2025, 37, 2501254. 10.1002/adma.202501254 40123197

[212] C. A. Vilela , C. Correia , J. M. Oliveira , R. A. Sousa , J. Espregueira‐Mendes , R. L. Reis , ACS Biomater. Sci. Eng. 2015, 1, 726. 33445249 10.1021/acsbiomaterials.5b00245

[213] C. Shen , J. Wang , G. Li , S. Hao , Y. Wu , P. Song , Y. Han , M. Li , G. Wang , K. Xu , H. Zhang , X. Ren , Y. Jing , R. Yang , Z. Geng , J. Su , Bioact. Mater. 2024, 35, 429. 38390528 10.1016/j.bioactmat.2024.02.016 PMC10881360

[214] E. A. Aisenbrey , W. L. Murphy , Nat. Rev. Mater. 2020, 5, 539. 32953138 10.1038/s41578-020-0199-8 PMC7500703

[215] X. Feng , J. M. McDonald , Annu. Rev. Pathol. 2011, 6, 121. 20936937 10.1146/annurev-pathol-011110-130203 PMC3571087

[216] M. Stanczak , B. Kacprzak , P. Gawda , Cell. Physiol. Biochem. 2024, 58, 677. 39568406 10.33594/000000743

[217] S. Patel , R. C. Ryals , K. K. Weller , M. E. Pennesi , G. Sahay , J. Control Release 2019, 303, 91. 30986436 10.1016/j.jconrel.2019.04.015 PMC6579630

[218] K. Morihiro , H. Osumi , S. Morita , T. Hattori , M. Baba , N. Harada , R. Ohashi , A. Okamoto , J. Am. Chem. Soc. 2023, 145, 135. 36538570 10.1021/jacs.2c08974

[219] Y. Li , S. Gao , S. Shi , D. Xiao , S. Peng , Y. Gao , Y. Zhu , Y. Li , Nanomicro Lett. 2021, 13, 86. 34138319 10.1007/s40820-021-00614-6 PMC8006527

[220] M. Lu , H. Xing , A. Zheng , Y. Huang , X. J. Liang , Acc. Chem. Res. 2023, 56, 224. 36624086 10.1021/acs.accounts.2c00464

[221] Y. Zheng , Lu Sun , Z. Zhai , F. Cao , T. Zhang , Q. Jiao , K. Xu , W. Zhong , Int. J. Biol. Macromol. 2024, 259, 129133. 38171439 10.1016/j.ijbiomac.2023.129133

[222] N. Didwischus , A. Guduru , S. F. Badylak , M. Modo , Acta Biomater. 2024, 174, 104. 38081445 10.1016/j.actbio.2023.12.003 PMC10775082

[223] T. Urbaitis , G. Gasiunas , J. K. Young , Z. Hou , S. Paulraj , E. Godliauskaite , M. M. Juskeviciene , M. Stitilyte , M. Jasnauskaite , M. Mabuchi , G. B. Robb , V. Siksnys , EMBO Rep. 2022, 23, 55481. 10.15252/embr.202255481 PMC9724661 36268581

[224] Xu Feng , R. Xu , J. Liao , J. Zhao , B. Zhang , X. Xu , P. Zhao , X. Wang , J. Yao , P. Wang , X. Wang , W. Han , Q. She , Nat. Commun. 2024, 15, 3464. 38658536 10.1038/s41467-024-47697-4 PMC11043419

[225] B. Husťáková , M. Trundová , K. Adámková , Tomáš Koval , J. Dusková , J. Dohnálek , FEBS Lett. 2023, 597, 2103. 37309731 10.1002/1873-3468.14683

[226] L. H. Goetz , N. J. Schork , Fertil. Steril. 2018, 109, 952. 29935653 10.1016/j.fertnstert.2018.05.006 PMC6366451

[227] T. Steinsberger , M. Donetti , M. Lis , L. Volz , M. Wolf , M. Durante , C. Graeff , Int. J. Radiat. Oncol. Biol. Phys. 2023, 115, 1257. 36462690 10.1016/j.ijrobp.2022.11.034

